# Fiddler Crabs (Crustacea: Decapoda: Ocypodidae) From Coastal Ecuador and the Galápagos Islands: Species Descriptions and DNA Barcodes

**DOI:** 10.1002/ece3.70646

**Published:** 2025-01-27

**Authors:** Carl L. Thurman, John C. McNamara, Hsi‐Te Shih, Mariana V. Capparelli

**Affiliations:** ^1^ Department of Biology University of Northern Iowa Cedar Falls Iowa USA; ^2^ Departamento de Biologia, FFCLRP Universidade de São Paulo São Paulo Brazil; ^3^ Department of Life Science and Global Change Biology Research Center National Chung Hsing University Taichung Taiwan; ^4^ Universidad Regional Amazónica Ikiam Tena Ecuador; ^5^ Instituto de Ciencias del Mar y Limnología Universidad Autónoma de México, Isla del Carmen Campeche Mexico

**Keywords:** cytochrome c oxidase‐subunit 1, diversity, fiddler crabs, morphology, neighbor‐joining tree

## Abstract

Neotropical regions near the equator are recognized as speciation “hot spots” reflecting their abundant biodiversity. In western South America, the coasts of Panama, Colombia, Ecuador, the Galápagos Archipelago, and northern Peru form the Tropical Eastern Pacific biome. This area has the greatest heterogeneity of sympatric fiddler crab species of any portion of the planet. Since the coastal fauna has not been assessed for almost 50 years, we studied fiddler crab species diversity in Ecuador and on the Galápagos Archipelago. Preserved collecting records for various species were examined at the U.S. National Museum of Natural History, Washington, DC, the American Museum of Natural History, New York, and the Naturalis Biodiversity Center, Leiden, the Netherlands. During a field study, 51 locations were collected resulting in over 870 preserved specimens (120 lots) along the 2237‐km (1390 mi) coast of Ecuador and on three Galápagos Islands. A neighbor‐joining tree was constructed using the Kimura 2‐parameter model with a partial DNA sequence of the cytochrome oxidase‐subunit 1 gene (COI) for a barcoding study. Twenty‐five taxa were collected during the surveys, while two more were noted from the literature and museum collections. Five published species are new to Ecuador. The species assemblage was divided among four genera: *Uca*, *Leptuca*, *Minuca,* and *Petruca*. Morphological definitions and photographic images are given for 27 species. COI sequences were obtained for 27 operational taxonomic units from Ecuador, with three morphologically indistinguishable cryptic or pseudocryptic taxa also revealed. Based on species distributions, it appears that the area between Cabo San Lorenzo and Punta Santa Elena serves as a weak barrier separating some “northern” from “southern” taxa. Since coastal Ecuador is undergoing rapid economic development, the construction of maricultural facilities and the deforestation of mangroves promote wholesale habitat destruction. As habitat diversity is reduced, it is expected that there will be, in general, a local decline in fiddler crab species diversity with some taxa becoming rare or extinct.

## Graphical abstract

Twenty‐five taxa were collected during the surveys, while two more were noted from the literature and museum collections; five are new to Ecuador. DNA sequences for cytochrome c oxidase–subunit 1 were obtained for 27 operational taxonomic units with three morphologically indistinguishable cryptic or pseudo‐cryptic taxa were also revealed.

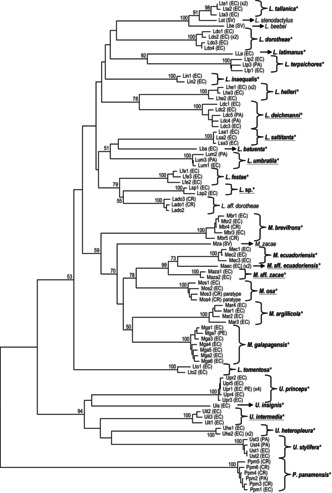

## 1 Introduction

From the perspective of global biodiversity, the Pacific Equatorial region of western South America constitutes the sixteenth world‐ranked biodiversity hot spot based on species abundance and endemism (Mittermeier et al. 1998; Brooks et al. 2002). In stark contrast, Ecuador suffers the highest annual rate of deforestation in South America (−21.5% between 1990 and 2005, Dodson and Gentry 1991; Tapia‐Armijos et al. 2015) while coastal habitats are being devastated at an alarming rate (Figueroa‐Pico et al. 2016; Morocho et al. 2022; Navarrete‐Forero et al. 2023; Jaramillo et al. 2023). Agriculture and mariculture have also encouraged habitat destruction (Merecí‐Guamán et al. 2021; Gouveia et al. 2023). Consequently, such habitat losses place Ecuador among the top 11 countries suffering tropical environmental destruction (Bendix and Beck 2009).

In Ecuador, marine communities are distributed along the 1600‐km long Pacific coast and throughout the Galápagos Archipelago. Near the Equator, along the coast of the eastern Pacific Ocean, the northern Panamanian and the southern Peruvian/Humboldt surface currents converge and flow westwards as the South Equatorial Current to and through the Galápagos Archipelago. The continental coast exhibits a great diversity of marine ecosystems where bays, beaches, estuaries, coastal lagoons, mangroves, cliffs, and rocky coasts are common (Cruz et al. 2003). The shores of the Galápagos Islands lie 1200 km west of Ecuador and consist predominantly of volcanic rocks with few brackish water spaces. Muddy or sandy substrates are uncommon on the remote and rugged Archipelago coasts (Bustamante, Collins, and Bensted‐Smith 2000; Hickman 2009). However, littoral and estuarine communities along the continental coast of Ecuador are interconnected with adjacent populations in Panama, Colombia, and Peru by near shore currents (Cruz et al. 2003; Aerts et al. 2004). Most likely, remoteness from the continental coast and low habitat diversity are the main factors that have led to a reduction in the richness of littoral species on the Galápagos Archipelago. However, despite fewer species, the level of littoral endemism is much greater on the islands than along the continental Ecuadorian shore (Peck 1994; Cruz et al. 2003; Hickman 2009).

On most beaches and estuaries in tropical and temperate regions, fiddler crabs (Ocypodidae Rafinesque, 1815) are a common component of the littoral fauna (Crane 1975). Currently, 108 extant species are recognized (Shih et al. 2016; Shih and Chan 2022; Thurman et al. 2023). Fiddler crabs were originally classified taxonomically in the genus *Gelasimus* Latreille, 1817. In 1897, Rathbun revised the nomenclature of the genus, re‐naming it *Uca* Leach, 1814. In a further revision, Crane (1975) altered *Uca* systematics substantially. While still recognizing just a single genus, Crane proposed nine subgenera including 92 taxa, many with subspecies designation. Since fiddler crabs are paraphyletic belonging to two subfamilies, the Gelasiminae and the Ocypodinae, Shih et al. (2016), based on molecular phylogenetics, revised the single‐genus concept and elevated all the subgenera to full genus status. Currently, 11 genera are used to organize the 108 fiddler crab species. Besides the fiddler crabs, there are two other genera, *Ocypode* in the Ocypodinae and *Ucides* in the Ucidinae Števčić, 2005, in the family Ocypodidae.

In her monograph, Crane (1975) also analyzed the geographic distributions of the new subgenera and subspecies. She recognized five to six distinct zoogeographical regions: (1) the west coast of Africa, (2) east Africa and the western Indian Ocean, (3) the eastern Indian Ocean and Southeast Asia, (4) the western Pacific and Australia, (5) the eastern Pacific, and (6) the western Atlantic coasts of the Americas (Crane 1975, 431–433, Map 1). The region of highest species diversity occurs along the eastern Pacific shores between El Salvador and Ecuador, containing four subgenera and 29 species (Crane 1975). Areas to the north or south show lower fiddler crab diversity with just eight species or less. Consequently, the coast of Ecuador appears to constitute a diversity hot spot for fiddler crabs.

The first comprehensive check list of fiddler crabs for Ecuador (Table 1) recognized 10 species (Rathbun 1918) that, using current nomenclature, were: *Uca insignis* (H. Milne Edwards, 1852), *U. princeps* (Smith, 1870), *U. stylifera* (H. Milne Edwards, 1852), *Leptuca festae* (Nobili, 1902), *L. helleri* (Rathbun, 1902), *L. latimanus* (Rathbun, 1897), *L. stenodactylus* (H. Milne Edwards and Lucas, 1843), *Minuca galapagensis* (Rathbun, 1902), and *Petruca panamensis* (Stimpson, 1859). Later, Rathbun included two more species: *Uca guayaquilensis* Rathbun, 1935 and *Uca inaequalis* Rathbun, 1935. However, *U. guayaquilensis* was found to be a junior synonym of *L. festae* (Crane 1975, 270; Shih et al. 2016, 153). From northern Peru, von Hagen (1968) expanded the species list to include *L. batuenta* (Crane, 1941), *L. beebei* (Crane, 1941), *L. dorotheae* (von Hagen, 1968), *L. tallanica* (von Hagen, 1968), *L. tenuipedis* (Crane, 1941), *L. tomentosa* (Crane, 1941), *L*. *terpsichores* (Crane, 1941), *U. heteropleura* (Smith, 1870), and *U. ornata* (Smith, 1870). However, Crane considered three of the species described by von Hagen (1968) to be synonyms of older taxa: *Uca pizarri* von Hagen, 1968 was recognized as *U. ornata* (Crane 1975, 153), *Uca lanigera* von Hagen, 1968 as *M. ecuadoriensis* (Crane 1975, 167), and *Uca mertensi* Bott, 1954 as *L. tomentosa* (Crane 1975, 263). von Prahl (1982) reported four additional species from a study of fiddler crabs along the Pacific coast of Colombia: *M. pygmaea* (Crane, 1941), *M. argillicola* (Crane, 1941), *L. umbratila* (Crane, 1941), and *L. deichmanni* (Rathbun, 1935). Three years later, von Prahl and Toro (1985) described *Uca intermedia*, a species new to the Pacific coast. In 2010, Landstorfer and Schubart reported *M. argillicola* from Puerto Morro, Guayas. Thus, the Pacific shoreline of northern South America appears to host approximately 27 species of fiddler crabs: 15 *Leptuca*, five *Minuca*, six *Uca*, and one *Petruca* (Table 1). Based on Crane's (1975) analysis, ≈93% of the fiddler crab species from the Pacific coast of El Salvador, Costa Rica, Panama, Colombia, and Peru can be found in Ecuador. On a global scale, 26% of all fiddler crab species occurred along the Pacific coast of Central and northern South America.

**TABLE 1:** Historic records of fiddler crabs in Ecuador and northwest South America.

Genus		Source	Current known range^a^
*Uca*	*insignis*	Rathbun (1918)	El Salvador to northern Peru
	*princeps*	Rathbun (1918)	USA to Chile
	*stylifera*	Rathbun (1918)	El Salvador to northern Peru
	*heteropleura*	von Hagen (1968)	El Salvador to northern Peru
	*ornata*	von Hagen (1968)	El Salvador to northern Peru
	*intermedia*	von Prahl and Toro (1985)	Panama to north Colombia
*Leptuca*	*festae*	Rathbun (1918)	El Salvador to Ecuador
	*helleri*	Rathbun (1918)	Galápagos Archipelago
	*latimanus*	Rathbun (1918)	Mexico to northern Peru
	*stenodactylus*	Rathbun (1918)	El Salvador to Chile
	*inaequalis*	Rathbun (1935)	Honduras to northern Peru
	*tenuipedis*	von Hagen (1968)	Honduras to Peru
	*batuenta*	von Hagen (1968)	Honduras to northern Peru
	*tallanica*	von Hagen (1968)	southern Ecuador to northern Peru
	*tomentosa*	von Hagen (1968)	El Salvador to northern Peru
	*terpichores*	von Hagen (1968)	El Salvador to northern Peru
	*dorotheae*	von Hagen (1968)	Costa Rica to northern Peru
	*beebi*	von Hagen (1968)	Honduras to northern Peru
	*saltitanta*	Crane (1975)	El Salvador to northern Colombia
	*umbratila*	von Prahl (1982)	El Salvador to Colombia
	*deichmanni*	von Prahl (1982)	Honduras to northern Colombia
*Minuca*	*galapagensis*	Rathbun (1918)	Costa Rica to Chile, Galápagos Arch.
	*herradurensis*	Rathbun (1918)	El Salvador to northern Peru
	*ecuadoriensis*	Maccagno (1928)	Mexico to northern Peru
	*pygmeae*	Prahal (1983)	Costa Rica to northern Colombia
	*argillicola*	Schubart (2009)	Costa Rica to Ecuador
*Petruca*	*panamensis*	Rathbun (1918)	El Salvador to northern Peru

^a^ Data taken from Rosenberg, M. S. *Fiddler Crabs*. www.fiddlercrab.info.

A comprehensive survey of fiddler crabs from the Pacific coast of South America has not been undertaken for more than 40 years (Crane 1975; von Prahl 1982). Focusing on newly recorded taxa, the current investigation revisits Ecuador and the Galápagos Archipelago to recover fiddler crab species from habitats located along the eastern Pacific shore between Colombia and Peru. The results of our investigation provide an accurate, contemporary checklist of species, employing DNA barcoding to verify geographic records. This study also establishes a convenient backdrop for ecological, morphologic, physiologic, and genetic investigations, as well as providing a platform for conservation efforts in Ecuador and the Galápagos Archipelago.

## 2 Materials and Methods

### 2.1 Fiddler Crab Collections

**FIGURE 1:**
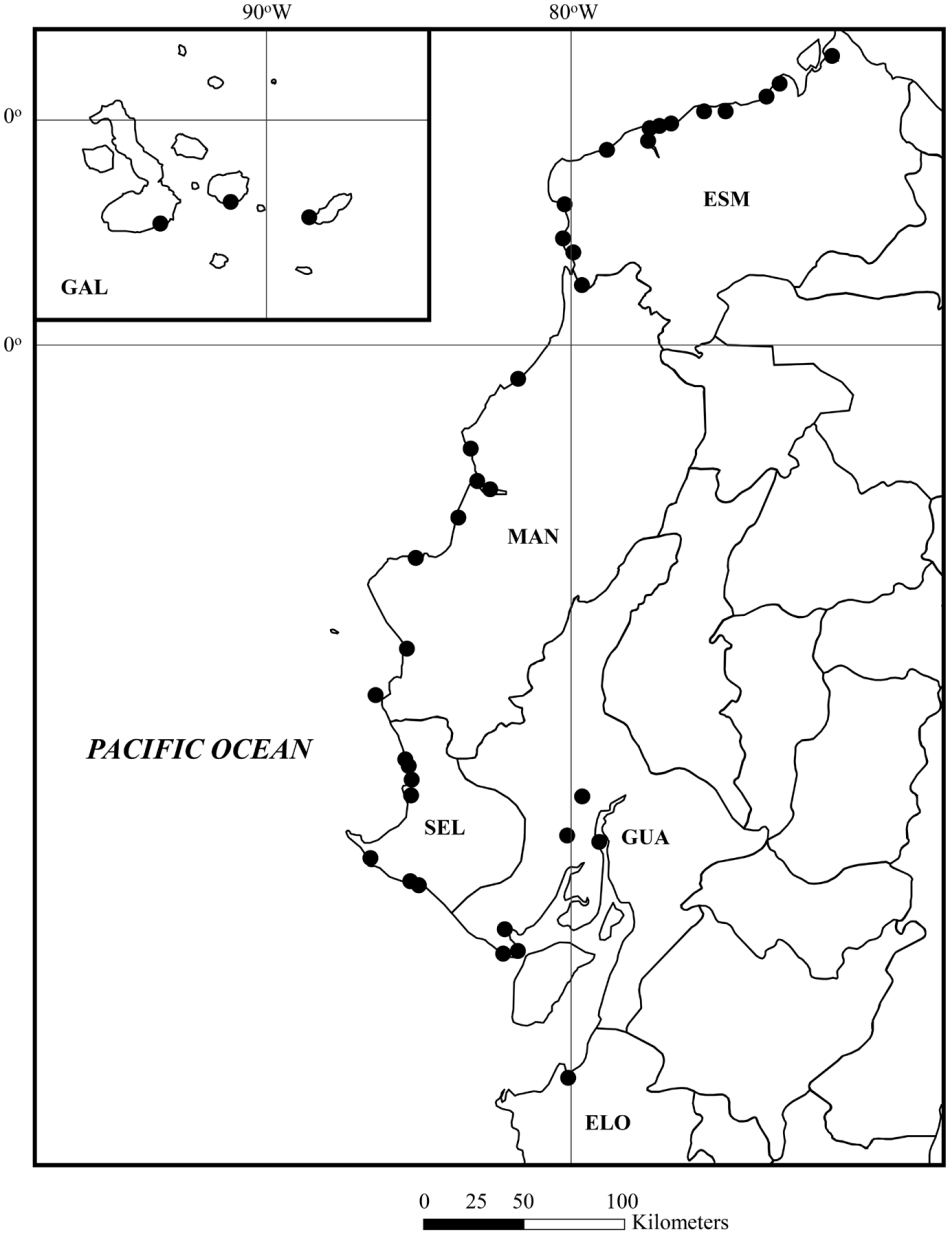
Collecting locations. Solid dot (•) marks field sites for collection of fiddler crabs in Ecuador and the Galapagos Archipelago during this study. See text. Provinces indicated by large letters: El Oro–ELO, Esmeraldas—ESM, Galapagos Archipelago—GAL, Guayas—GUA, Manabi—MAN, and St Elena—SEL.

Field collections of fiddler crabs were authorized by the Ministerio del Ambiente de Ecuador, Dirección Nacional de Biodiversidad, Unidad de Acceso a Recursos Genéticos, Quito (contract number MAE‐DNB‐CM‐2017‐0062; September 24, 2018 until November 10, 2021). The collections were conducted at 42 localities along the Pacific coast of Ecuador, and at nine locations on Isla Santa Cruz, Isla San Cristóbal, and Isla Isabela in the Galápagos Archipelago (Figure 1, Table S1). References to provincial permits for the transport and export of specimens are given in the Acknowledgements section.

Between November 2018 and November 2019, four expeditions were undertaken to collect fiddler crabs from: (1) southern Ecuador (November 16–27, 2018); (2) the state of Esmeraldas (March 16–23, 2019); (3) the Galápagos Islands Archipelago (June 30–July 6, 2019); and (4) the Provinces of St. Elena and southwest Guayas (November 25–30, 2019). The collection sites chosen (Figure 1) provided convenient access to coastal habitats by road, track vehicle, or boat. Collections at each location were conducted at random to assure adequate species diversity.

The following abbreviations are used here and throughout for the Ecuadorian Provinces (Figure 1): El Oro—ELO, Esmeraldas—ESM, Galápagos Archipelago—GAL, Guayas—GUA, Manabí—MAN, and Santa Elena—SEL.

### 2.2 Species Identification and Measurements

For this investigation, we employed the recent taxonomy of the Ocypodidae proposed by Shih et al. (2016). The Ocypodidae consists of three subfamilies: the Ocypodinae Rafinesque, 1815; the Gelasiminae Miers, 1886; and the Ucidinae Števčić, 2005. In the Americas, four fiddler crab genera are recognized: (1) genus *Uca* Leach, 1814; (2) genus *Petruca* Shih, Ng and Christy, 2015; (3) genus *Leptuca* Bott, 1973; and (4) genus *Minuca* Bott, 1954. The “narrow‐fronted” species in the genus *Uca* are members of the subfamily Ocypodinae, while those in the “broad‐fronted” genera *Leptuca*, *Minuca*, and *Petruca* are from the subfamily Gelasiminae.

To identify the fiddler crab species, several authoritative sources were consulted: Rathbun (1918, 1935), Crane (1941, 1975), von Hagen (1968), and von Prahl and Toro (1985). In addition to our recent field collections, preserved specimens from several major collections were examined and photographed (Table 2, Table S2). Crabs from the collection of Frank H. Barnwell, University of Minnesota, Twin Cities, were examined and delivered to the American Museum of Natural History (AMNH), NYC in August 2022. AMNH_IZC specimens can be located online at https://emu‐prod.amnh.org/imulive/iz/iz.html. Specimens in the collection at the US National Museum of Natural History (USNM), Washington, DC are listed at http://collections.nmnh.si.edu/search/iz. To confirm their definitions, type and voucher specimens were examined and photographed on June 3–5, 2019 (Table 2). In addition, examples of *Uca insignis* (USNM 138577), *U. ornata* (USNM 138615), *Leptuca tallanica* (USNM 138838) and *L. tenuipedis* (USNM 137410) were borrowed for additional, detailed photographs. Preserved specimens from the eastern Pacific Ocean at the Naturalis Biodiversity Center (NBC) in Leiden, the Netherlands, were examined on January 3–5, 2024 (https://bioportal.naturalis.nl). Among the specimens, the types for *Minuca osa* (RMNH.CRUS.D53098), *U. intermedia* (RMNH.CRUS.D23063), *L. dorotheae* (RMNH.CRUS.D23054 and RMNH.CRUS.D23055), and *L. tallanica* (RMNH.CRUS.D23046 and RMNH.CRUS.D23047) were examined and photographed. A single lot of eight specimens of *M. zacae* (ZMA.CRUS.D100.365) from the Zoological Museum of Amsterdam (ZMA) was examined and photographed. The ZMA specimens are located at the NBC but not currently cataloged. Crab images were captured using a Nikon D40 digital camera equipped with either a 105 mm NIKKOR AF‐S macro lens or an 18–55 mm Nikon DX AF‐S zoom lens. Final images were prepared using Adobe Photoshop CC 2018.

**TABLE 2:** Reference specimens for fiddler crabs from northwest South America. Examined at USNM—U.S. National Museum of Natural History, Washington DC, and RMNH.CRUS.D—Naturalis Biodiversity Center, Leiden, Netherlands.

Specimen number	Species	Collecting location
Genus *Uca*		
USNM 138577	*Uca insignis*	Puerto Bolivar, Ecuador; Lima, Puerto Pizarro, Peru
1521395		
RMNH, CRUS.D023063		Puerto Pizarro (formerly *U. pizarri*)
RMNH.CRUS.D35788	*Uca intermedia*	Bahia de Buenavista, Colombia
USNM 138550	*Uca heteropleura*	Puerto Bolivar, Ecuador
USNM 138615	*Uca ornata*	Rio Abajo, Old City, Panama
134616		
USNM 138633	*Uca princeps*	Puerto Bolivar, Ecuador
138634		
USNM 138837	*Uca stylifera*	Puerto Bolivar, Ecuador (decomposed)
32325		
Genus *Leptuca*		
USNM 13404	*Leptuca batuenta*	Puntarenas, Costa Rica; Puerto Bolivar Ecuador
137405, 137406		
381136		
RMNH.CRUS.D023045	*Leptuca batuenta*	Puerto Pizarro, Peru
USNM 138413	*Leptuca beebei*	Balboa Island, Panama; Puerto Bolivar, Ecuador
138474, 138480		
USNM 70832	*Leptuca deichmanni*	Canal Zone, Panama
138525		
USNM 138534	*Leptuca dorotheae*	Puerto Bolivar, Ecuador
RMNH.CRUS.D023055	*Leptuca dorotheae*	Puerto Pizarro, Peru
USNM 70870	*Leptuca festae*	Gulf of Guayaquil, Ecuador
USNM 24829	*Leptuca helleri*	Narborough Island, Galápagos Archipelago
USNM 70833	*Leptuca inaequalis*	Salado mangroves, Guayaquil, Ecuador
USNM 17500	*Leptuca latimanus*	Balboa Island, Panama; Puerto Bolivar Ecuador (decomposed)
138565, 138566		
USNM 79401	*Leptuca limicola*	Golfito, Gulf of Dulce, Costa Rica (damaged)
137415, 137416		
USNM 138822	*Leptuca saltitanta*	Balboa Island, Panama
USNM 138838	*Leptuca tallanica*	Puerto Bolivar, Ecuador
RMNH.CRUS.D 023044	*Leptuca tallanica*	Puerto Pizarro, Peru
USNM 79404	*Leptuca tenuipedis*	Ballena Bay, Costa Rica
137409, 137409		
USNM 137417	*Leptuca terpsichores*	Canal Zone, Balboa, Panama
137418		
USNM 79406	*Leptuca tomentosa*	Puntarenas and Golfito, Costa Rica; Gulf of Panama, Panama City
137411, 13412		
138839, 1521275		
RMNH.C RUS.D023051		Puerto Pizarro, Ecuador (formerly *U. mertensi* )
USNM 134826	*Leptuca stenodactylus*	Corinto, Nicaragua; Costa Rica, Bella Vista, Panama; Puerto Bolivar, Ecuador
138830, 138132		
138833		
USNM 79407	*Leptuca umbratila*	Puntarenas, Nicoya, Costa Rica; Canal Zone, Panama
18133		
Genus *Minuca*		
USNM 19441	*Minuca argillicola*	Santo Domingo and Golfito, Gulf of Dulce, Costa Rica
137400, 137401		
USNM 138484	*Minuca brevifrons*	Negritos Island, Costa Rica
USNM 70867	*Minuca ecuadoriensis*	San Blas, Nayarit, Mexico; Mangroves, Salado, Guayaquil, Ecuador
97756		
RMNH.CRUS.D023049	*Minuca ecuadoriensis*	Puerto Pizarro, Peru (formerly *U. lanigra*)
RMNH.CRUS.D023050	*Minuca ecuadoriensis*	Puerto Pizarro, Peru (formerly *U. lanigra*)
USNM 22319	*Minuca galapagensis*	Isla Santa Cruz, Galápagos Archipelago; Guayaquil, Ecuador
98046, 138538		
USNM 123790	*Minuca herradurensis*	Mata de Limon, Costa Rica; Panama City, Panama
138542		
RMNH.CRUS.D53098	*Minuca osa*	Gulf of Dulce, Osa peninsula, Costa Rica
53126		
USNM 79402	*Minuca pygmaea*	Golfito, Costa Rica; Buenaventura, Colombia Specimens decomposed or missing
137419, 137420		
18646		
USNM 137426	*Minuca zacae*	Corinto, Nicaragua; Golfito, Gulf of Dulce, Costa Rica; Type specimens poor
137427, 138646		
1521269		
Genus *Petruca*		
USNM 1294205	*Petruca panamensis*	Neotype, Culebra Island, Panama; Costa Rica; Gorgonilla Island, Colombia
138625, 138629		

In general, at least five individual specimens were examined using a stereoscopic binocular microscope for detailed morphology. For measurements accurate to 0.01 mm, a digital caliper was used. For very small specimens, a small drafting divider was used to estimate a dimension, and the distance between the tips was then measured with a digital caliper. Since specimens varied considerably in size, measurements were converted to allometric percentage relationships between structures (e.g., carapace length vs. width, interocular [frontal] width vs. carapace width, merus width vs. length). Individual percentages were averaged (*n* = 5) and found to vary no more than 3.0%. These values were rounded to the nearest percent.

### 2.3 Molecular Methods

Fiddler crab pereiopods were obtained by autonomy and fresh samples of muscle tissue were preserved in 95% ethanol for future analysis of DNA sequences. Some crabs were then returned live to their collecting sites. Over 870 whole adult crabs were preserved in 80% ethanol as voucher specimens after freezing at −5°C for 1 h (Table S2). Since the following species were not located during the field collections, tissues from *Uca insignis* (USNM 138577) and *U. ornata* (USNM 138615) were donated by the Smithsonian Institution (USNM) for DNA analysis. Tissue samples from *Leptuca tallanica* (USNM 138838) and *L. tenuipedis* (USNM 137410) also were analyzed to verify identification. Unfortunately, none of the four USNM samples produced usable DNA sequences.

Genomic DNA was isolated from the pereiopod muscle tissues using extraction kits (see Shih et al. 2016 for details). A partial sequence of the cytochrome c oxidase‐subunit 1 gene (COI) was amplified by polymerase chain reaction (PCR) using the primers LCO1490, HCO2198 (Folmer et al. 1994), COL14 (Roman and Palumbi 2004), COH6 (Schubart 2009), LOB, HCOex3 (Shih et al. 2022), LCOex3 (Shih, Naruse, and Schubart 2023), HCOex0 (Shih, Hsu, and Li 2023), and COH900 (Schubart et al. 2023). PCR conditions consisted of pre‐denaturation for 2 min at 94°C; 40 cycles of denaturation for 50 s each at 94°C, annealing for 70 s at 45°C–47°C, extension for 60 s at 72°C, followed by a final extension period of 10 min at 72°C. DNA sequences were obtained by automated sequencing (Applied Biosystems 3730, ThermoFisher Scientific) and deposited in the NCBI GenBank (Table 3).

**TABLE 3:** Haplotypes of COI marker for fiddler crab species from Ecuador and related outgroups. Abbreviations for repositories are detailed in Materials AND Methods. NCHUZOOL—the Zoological Collection of the Department of Life Sciences, National Hsing University, Taichung, Taiwan; ZRC—the Zoological Reference collection of the Lee Kong Chian Natural History Museum, National University of Singapore. The abbreviations for generic names: *L.—Leptuca, M.—Minuca, U.—Uca*, and *P.—Petruca*.

Species	Locality	Catalog no.	Sample size	Haplotype	Access. no.
*Leptuca*					
*L. batuenta*	Ecuador: El Rompido, Esmeraldas	UNI 861	1	Uba	PQ524227
*L. beebei*	El Salvador: Usulutan	SMF 48165	1	Lbe	PQ524226
*L. deichmanni*	Ecuador: Playa Achilube, Esmeraldas	UNI P0050	1	Ldc1	PQ524228
	Ecuador: Mompiche, Esmeraldas	UNI 792	1	Ldc2	PQ524229
	Ecuador: San Lorenzo, Esmeraldas	UNI 767	1	Ldc3	PQ524230
	Panama: Culebra Island	NCHUZOOL 13583	1	Ldc4	AB813676
	Panama: Culebra Island	NCHUZOOL 13583	1	Ldc5	PQ524231
*L. dorotheae*	Ecuador: Chamanga, Esmeraldas	UNI P0269	1	Ldo1	PQ524232
	Ecuador: Punta Carnero, South Salinas, St. Elena	UNI P0271; UNI 773	2	Ldo2	PQ524233; PQ524234
	Ecuador: Bahia de Caraquez, Manabi	UNI P0306	1	Ldo3	PQ524235
	Ecuador: San Jose de Chamanga, Esmeraldas	UNI 771	1	Ldo4	PQ524236
*L*. aff. *dorotheae*	Costa Rica: Tempisque R.	ZRC	1	Lado1	LC087961
	Costa Rica: Tempisque R.	ZRC	1	Lado2	PQ524237
	Costa Rica: Tempisque R.	ZRC	1	Lado3	PQ524238
*L. festae*	Ecuador: Los Lojas, Guayaquil, Guayas	UNI P0212	1	Lfe1	PQ524239
	Ecuador: Isla Santay, Guayas	UNI 751	1	Lfe2	PQ524240
	Ecuador: Sta Marta, Esmeraldas	UNI 774	1	Lfe3	PQ524241
*L. helleri*	Ecuador: Santa Cruz Island, Galápagos.	UNI P0102; UNI P0103	2	Lhe1	PQ524242; PQ524243
	Ecuador: Santa Cruz Island, Galápagos.	UNI 778	1	Lhe2	PQ524244
	Ecuador: Santa Cruz Island, Galápagos.	UNI 777	1	Lhe3	PQ524245
*L. inaequalis*	Ecuador: San Lorenzo, Esmeraldas	UNI P0273	1	Lin1	PQ524246
	Ecuador: San Lorenzo, Esmeraldas	UNI P0274	1	Lin2	PQ524247
*L. latimanus*	Ecuador: El Real, St. Elena	UNI P0152	1	LLa	PQ524248
*L. saltitanta*	Ecuador: El Rompido, Esmeraldas	UNI P0282	1	Lsa1	PQ524249
	Ecuador: El Rompido, Esmeraldas Ecuador, Mompiche, Esmeraldas	UNI 795 UNI 866	1 1	Lsa2	PQ524250
	Ecuador: San Lorenzo, Esmeraldas	UNI 869	1	Lsa3	PQ524251
*L. stenodactylus*	El Salvador	SMF 2357	1	Lst	LC150749
*L. tallanica*	Ecuador: Machala, El Oro; Posorja, Guayas	UNI P279; UNI P277	2	Lta1	PQ524252; PQ524253
	Ecuador: Machala, El Oro	UNI P0280	1	Lta2	PQ524254
	Ecuador: Puerto del Morro, Guayas	UNI P0117	1	Lta3	PQ524255
*L. terpsichores*	Ecuador: San Lorenzo, Esmeraldas	UNI 798	1	Ltp1	PQ524256
	Ecuador: Mompiche, Esmeraldas	UNI 797	1	Ltp2	PQ524257
	Panama: Culebra Island	NCHUZOOL 13582	1	Ltp3	AB813677
*L. tomentosa*	Ecuador, Data deVillamil, Guayas	UNI P0217	1	Lto1	PQ524258
	Ecuador, Data deVillamil, Guayas	UNI P0218	1	Lto2	PQ524259
*L. umbratila*	Ecuador: Rio Verde, Esmeraldas	UNI P0027	1	Lum1	PQ524260
	Panama: Diablo Heights	NCHUZOOL 13579	1	Lum2	FN430708
	Panama: Diablo Heights	NCHUZOOL 13579	1	Lum3	AB813679
*L*. sp.	Ecuador: Esmeraldas	UNI P0289	1	Lsp1	PQ524261
	Ecuador: Esmeraldas	UNI P0290	1	Lsp2	PQ524262
*Minuca*					
*M. argillicola*	Ecuador: La Tola, Esmeraldas	UNI P0016	1	Mar1	PQ524263
	Ecuador: Punta Portete, Mompiche, Esmeraldas	UNI P0299	1	Mar2	PQ524264
	Ecuador: Punta Portete, Mompiche, Esmeraldas	UNI P0300	1	Mar3	PQ524265
	Ecuador: Puerto Morro	SMF 34737	1	Mar4	FN430701
*M. brevifrons*	Ecuador: La Tola, Esmeraldas	UNI P0009	1	Mbr1	PQ524266
	Ecuador: La Propicia, Esmeraldas	UNI 832	1	Mbr2	PQ524267
	Ecuador: Daule, Esmeraldas	UNI P0302	1	Mbr3	PQ524268
	Costa Rica: Playa San Juanillo	ZRC 2012.0126	1	Mbr4	LC087949
	Costa Rica: Bahia Caña Blanca	SMF 34738	1	Mbr5	FN430702
*M. ecuadoriensis*	Ecuador: Palmar, Puente Sitio Nuevo, St. Elena	UNI P0132	1	Mec1	PQ524269
	Ecuador: Esmeraldas, La Tola	UNI 811	1	Mec2	PQ524270
	Ecuador: Puerto Morro	SMF 34740	1	Mec3	FN430704
*M*. aff. *ecuadoriensis*	Ecuador: Rio Guayas, Guayaquil, Guayas Ecuador: Isla Santay, Guayas	UNI P0224, UNI 746	2	Maec	PQ524271; PQ524272
*M. galapagensis*	Ecuador: Santa Cruz, Galápagos	UNI P0068	1	Mga1	PQ524273
	Ecuador: Simon Bolivar, St. Elena	UNI P0168	1	Mga2	PQ524274
	Ecuador: Simon Bolivar, St. Elena	UNI P0133	1	Mga3	PQ524275
	Ecuador: San Clemente, Manabi	UNI P0209	1	Mga4	PQ524276
	Ecuador: San Clemente, Manabi	UNI P0208	1	Mga5	PQ524277
	Ecuador; Puerto Morro	SMF 34741	1	Mga6	FN430705
	Peru: Tumbes	SMF 13151	1	Mga7	PQ524278
*M. osa*	Ecuador: Simon Bolivar, St. Elena	UNI P0123	1	Mos1	PQ524279
	Ecuador: La Tola, Esmeraldas	UNI P0303	1	Mos2	PQ524280
	Costa Rica: Golfo Dulce	(paratype)	1	Mos3	FN430711
	Costa Rica: Golfo Dulce	(paratype)	1	Mos4	FN430712
*M. zacae*	El Salvador	SMF 2104a	1	Mza	FN430710
*M*. aff. *zacae*	Ecuador: Rio Verde, Esmeraldas	UNI P0040	1	Maza1	PQ524281
	Ecuador: Rio Verde, Esmeraldas	UNI P0041	1	Maza2	PQ524282
*Petruca panamensis*	Ecuador: Playa Camerones, Esmeraldas	UNI P0293	1	Ppm1	PQ524283
	Panama: Culebra Island	NCHUZOOL 13581	1	Ppm2	LC087944
	Costa Rica	ZRC	1	Ppm3	PQ524284
	Costa Rica	ZRC	1	Ppm4	PQ524285
	Costa Rica: San Juanillo, Ostional	NCHUZOOL 14753	1	Ppm5	LC087945
	Costa Rica: Playa San Juanillo	ZRC 2012.0126	1	Ppm6	LC087948
*Uca*					
*U. heteropleura*	Ecuador: Salinas, Posorja, Guayas	UNI P0240	1	Uhe1	PQ524286
	Ecuador: Posorja, Guayas	UNI 743	2	Uhe2	PQ524287; PQ524288
*U. insignis*	Ecuador: Puerto Morro	SMF 34743	1	Uis	FN430707
*U. intermedia*	Ecuador: El Rompido, Esmeraldas	UNI 754	1	Uit1	PQ524289
	Ecuador: El Rampido, Esmeraldas	UNI 754	1	Uit2	PQ524290
	Ecuador: El Rampido, Esmeraldas	UNI 881	1	Uit3	PQ524291
*U. princeps*	Ecuador: Puenta Carnero, South Salinas, St. Elena; Bahia de Caraquez, Manabi; Posorja, Guayas Peru: Tumbez	UNI P0197; UNI P0252; UNI 741 SMF 13164	4	Upr1	PQ524292; PQ524293; PQ524294; LC150448
	Ecuador: Salinas, Posorja, Guayas	UNI P0241	1	Upr2	PQ524295
	Ecuador: Puerto del Morro, Guayas	UNI P0199	1	Upr3	PQ524296
	Ecuador: San Clemente, Manabi	UNI 745	1	Upr4	PQ524297
	Ecuador: Puerto del Morro, Guayas	UNI P0195	1	Upr5	PQ524298
*U. stylifera*	Ecuador: Bahia de Caraquez, Manabi	UNI P0260	1	Ust1	PQ524299
	Ecuador: Punta Portete, Mompiche, Esmeraldas	UNI P0286	1	Ust2	PQ524300
	Panama: Rodman	NCHUZOOL 13578	1	Ust3	LC053379
	Panama: Rodman	NCHUZOOL 13578	1	Ust4	PQ524301
Total			97		

Additional COI sequences for species from adjacent geographic regions or from related species were downloaded from NCBI GenBank to aid in confirming species identification. As a neighbor‐joining (NJ) tree with the Kimura (1980) 2‐parameter (K2P) model is typically applied in barcode studies (Hebert, Penton et al. 2004; Hebert, Stoeckle et al. 2004; Hebert and Gregory 2005), the tree was generated with MEGA (vers. 11, Tamura, Stecher, and Kumar 2021) with the complete deletion option and 2000 bootstrap reiterations in our study. K2P distances among specimens, nucleotide composition, variable and parsimoniously informative positions were also calculated in MEGA using the pairwise deletion option.

## 3 Results

From 51 locations along the coast of Ecuador and the Galápagos Archipelago (Figure 1, Table S1), we collected more than 870 specimens representing 27 taxa of fiddler crabs along with about 300 muscle tissue samples. Five of these species are new to Ecuador. Two additional species, *Uca insignis* and *U. ornata* , have been documented in Ecuador, but we did not recover any examples. For these, voucher museum specimens were examined. Initially, the identity of each taxon was assessed using morphology and then verified by molecular analysis of COI sequences. For genetically distinct but morphologically identical taxa, the notation of “species affinis” or “aff.” is used and listed with the named species, including *M*. aff. *ecuadoriensis* and *M*. aff. *zacae*. Since additional research is required, these as well as one unidentified species (*L*. sp.) will be described in the future. The 27 published Ecuadorian species were sorted using the systematics of Shih et al. (2016) and the World Register of Marine Species (WoRMS 2024). Anatomical nomenclature is from Crane (1975: Figures 1, 2, 3) and the type locations from the original descriptions. Geographic ranges are from the fiddler crab information website (https://fiddlercrab.info/) or recent publications.

Phylum Arthropoda

Subphylum Crustacea

Class Malacostra

Order Decapoda

Family Ocypodidae Rafinesque, 1815

Subfamily Ocypodinae Rafinesque, 1815

Genus *Uca* Leach, 1814

*Uca heteropleura* (Smith, 1870)

*Uca insignis* (H. Milne Edwards, 1852)

*Uca intermedia* von Prahl and Toro, 1985

*Uca ornata* (Smith, 1870)

*Uca princeps* (Smith, 1870)

*Uca stylifera* (H. Milne Edwards, 1852)

Subfamily Gelasiminae Miers, 1886

Genus *Petruca* Shih, Ng and Christy, 2015

*Petruca panamensis* (Stimpson, 1859)

Genus *Minuca* Bott, 1954

*Minuca argillicola* (Crane, 1941)

*Minuca brevifrons* (Stimpson, 1860)

*Minuca ecuadoriensis* (Maccagno, 1928)

*Minuca galapagensis* (Rathbun, 1902)

*Minuca osa* (Landstorfer and Schubart, 2010)

Genus *Leptuca* Bott, 1973

*Leptuca batuenta* (Crane, 1941)

*Leptuca beebei* (Crane, 1941)

*Leptuca deichmanni* (Rathbun, 1935)

*Leptuca dorotheae* (von Hagen, 1968)

*Leptuca festae* (Nobili, 1901)

*Leptuca helleri* (Rathbun, 1902)

*Leptuca inaequalis* (Rathbun, 1935)

*Leptuca latimanus* (Rathbun, 1897)

*Leptuca saltitanta* (Crane, 1941)

*Leptuca stenodactylus* (H. Milnes Edwards and Lucas, 1843)

*Leptuca tallanica* (von Hagen, 1968)

*Leptuca tenuipedis* (Crane, 1941)

*Leptuca terpsichores* (Crane, 1941)

*Leptuca tomentosa* (Crane, 1941)

*Leptuca umbratila* (Crane, 1941)

### 3.1 Morphological Definitions

Subfamily Ocypodinae Rafinesque, 1815

Genus *Uca* Leach, 1814

Six species—Frontal region very narrow, much < 15% of carapace width. Eyestalks exceptionally long. Lower margin of the orbital with large denticles. Pollex and dactyl of major cheliped broad and flattened. Tip of dactyl never curving over pollex. Palm of the major cheliped with an oblique ridge. Intersection of the anterior and lateral margins of the carapace forming a sharp, outward‐turned spine.

**FIGURE 2:**
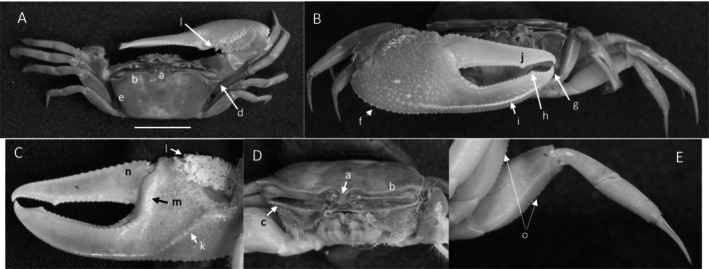
*Uca heteropleura* (Smith, 1870) (UNI 743) collected Posorja, GUA. (A) Dorsal view of male. Bar = 10 mm scale. (B) Front view. (C) Inner cheliped. (D) Ocular view. (E) Third ambulatory. a—interocular space, b—upper orbital, c—suborbital dentations, d—dorsal lateral angle, e—lateral margin, f—subchelar keel, g—pollex terminal tooth, h—preterminal tooth, i—pollex groove with pubescence, j—dactyl preterminal tooth, k‐ oblique ridge, l—carinal tooth, m—pollex‐gap line of tubercles, n—serrated dorsal edge of pollex, and o—serrations on posteroventral merus.

***Uca heteropleura*** (Smith, 1870) (Figure 2A–E). Moderate‐sized, narrow‐fronted species. Maximum carapace width approximately 24 mm. Frontal region (Figure 2A.a,D.a) about 5% carapace width. Upper orbital margin (Figure 2A.b,D.b) curving, sinusoid form. No eyebrows from a dorsal view. Eyestalk on the cheliped side in adults may have a short stylet. Suborbital margin (Figure 2D.c) with large square dentations. Carapace length about 62% width; surface finely granular. Antero‐lateral angle sharp and pointing outward (Figure 2A.d); lateral margins (Figure 2A.e) long with large distinct tubercle beads. Outer surface of major cheliped (Figure 2B) with numerous large tubercles spread over the surface; lower margin of manus with a row of larger tubercles (Figure 2B.f), “keel” formed by large tubercles. Manus and pollex of major cheliped approximately equal in length. When fingers are closed, gap almost same width as dactyl base (Figure 2B,C); outer surface of dactyl and pollex rugose with moderate‐sized tubercles. Large subterminal tooth on distal dactyl (Figure 2B.j). Below gap, a row of large tubercles along dorsal pollex margin; large triangular tooth near terminus (Figure 2B.h). End of pollex and dactyl with spinous hook (Figure 2B.g). Lower pollex margin with a row of large tubercles forming a groove, lined with pile (Figure 2B.i). Palm of manus with an oblique ridge (Figure 2C.k) terminating in carpal cavity, not forming a carina. Terminal tubercle forming a prominent central spine at the cavity's lower edge; upper edge of cavity with a single large tubercle and pile (Figure 2A.l,C.l). Inner surface of dactyl and pollex smooth and concave. On the upper edge of pollex, a row of large tubercles beginning on articulation cuff and extending to the distal tip of pollex (Figure 2C.m). Dorsal edge of dactyl serrated (Figure 2C.n). Walking legs (Figure 2E) smooth with a few setae and little pile; posteroventral edge of merus with small spinous tubercles (Figure 2E.o). Type location: Gulf of Fonseca, Salvador. Range: Honduras to northern Peru.

**FIGURE 3:**
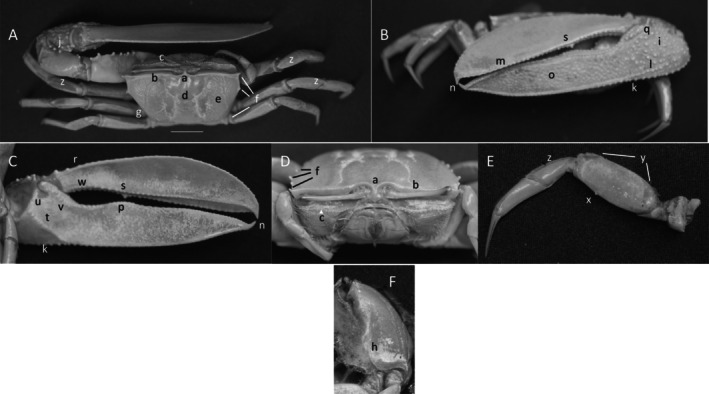
*Uca insignis* (H. Milne Edwards, 1952) (USNM 138577), Puerto Bolivar, ELO. (A) Dorsal view of male. Bar = 10 mm scale. (B) Front view. (C) Inner cheliped. (D) Ocular view. (E) Third ambulatory. (F) Lareal surface. a—frontal region, b—anterior margin, c—lower orbital margin, d—H‐depression, e—venation, f—lateral margin spines, g—venation origin, h—lower lateral carapace margin, i—manus tubracles, j—upper manus spines, k—submanus keel, l—external oblique tubercle row, m—overlap region, n—termianl spine, o—pits, p—ridge of dorsal pollex, q—articulation junction, r—dorsal dactyl ridge, s—dactyl tooth, t—oblique inner manus ridge, u—tubercles on anterior edge of carpal cavity, v—line of tubercles, w—line of larger dactyl tubercles, x—ventral merus spike, y—setae and pubescence, and z—pile on dorsal carpus.

***Uca insignis*** (H. Milne Edwards, 1852) (Figure 3A–F) (USNM 138577). Large species. Carapace width up to 46 mm. Frontal region (Figure 3A.a,D.a) approximately 4.2% carapace width. Anterior margin smooth and gently arching with sharp spine at anterior lateral angle (Figure 3A.b,D.b). No eyebrow from dorsal view. Eyestalks long with no terminal stylet. Lower orbital margin with large beads (Figure 3A.c,D.c). Carapace length about 74% of carapace width; center of carapace shiny but granular. H‐depression prominent (Figure 3A.d) with pubescence in suture junctions. Large swellings on mid‐line: anterior just behind frontal region, another posterior to H‐depression, third near posterior edge of carapace. Surface of brachial chambers with prominent venation (Figure 3A.e). Lateral carapace margin distinct with three to five spines (Figure 3A.f,D.f): one anterior, second slightly posterior and third on distal end of posterior‐lateral margin. Posterior lateral margin becoming weak and curving inward to midline. Origin of venation on posterior lateral line between medial and posterior spine (Figure 3A.g). Posterior margin of carapace raised and smoothly rounded, lateral end becoming tuberculated, turning upward (Figure 3F.h). Manus of larger propodus with large irregularly spaced tubercles (Figure 3B.i); inner dorsal margin of manus of cheliped with 3–6 large tooth‐like tubercles and long; thin setae (Figure 3A.j). Submarginal tubracated keel extending from manus heel to distal tip of dactyl (Figure 3B.k,C.k). A shorter tubercle ridge extending obliquely across lower manus intersecting ventral keel (Figure 3B.l). Pollex and dactyl long and blade‐like composing 73% of major cheliped and overlap distally (Figure 3B.m). Gap between dactyl and pollex small, widest near dactyl articulation. Dorsal and ventral edge of pollex lined with row of large tubercles terminating distally in a sharp spike (Figure 3B.n,C.n). Outer pollex covered with large circular depressions or pits (Figure 3B.o). Upper edge of pollex with row of low tubercles extending full length, proximal third forming a ridge of largest teeth (Figure 3C.p). Dactyl articulation (Figure 3B.q) almost horizontal, covered sparsely with pile at junction. Dorsal edge of dactyl with large, evenly spaced tubercles and ridge (Figure 3C.r). Lower margin of dactyl lined with tubercles and spine at distal end (Figure 3B.n,C.n). One large dactyl tooth aligned with elevated ridge on pollex (Figure 3C.s). Outer surface of dactyl smooth; inner surface of manus smooth with oblique ridge of large tubercles extending from carpal cavity to keel (Figure 3C.t). Junction of manus and pollex forming shallow sulcus. Anterior and ventral edge of carpal cavity without carina. Two large tubercles on anterior carpal margin (Figure 3C.u). Prominent line of tubercles extending distally along upper edge of pollex (Figure 3C.v). Inner surface of dactyl smooth with line of prominent tubercles along gape, largest tubercles proximal (Figure 3C.w). Merus of walking legs much longer than wide (Figure 3E); ventral surfaces smooth with one or two spikes on second or third ambulatory leg (Figure 3E.x). Proximal and distal merus with setae/pile and ridge of pile, respectively (Figure 3E.y). Dorsal surface of carpus with pubescence (Figure 3A.z,E.z). Type location: “Chili,” Gulf of Fonseca, Salvador. Range: El Salvador to northern Peru.

**FIGURE 4:**
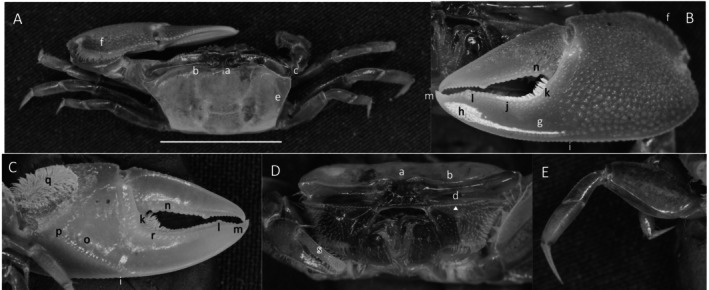
*Uca intermedia* von Prahl and Toro, 1985 (UNI 754) collected El Rompido, ESM. (A) Dorsal view of male. Bar = 10 mm scale. (B) Front view of large cheliped. (C) Inner cheliped. (D) Ocular view. (E) Third ambulatory. a—interocular space, b—frontal margin, c—dorsal lateral angle, d—suborbital dentations, e—dorsolateral marigin, f—large bristles, g—submarginal grove, h—terminal pubescence, I—subchela keel, j—row of tubercles, k—proximal gape bristles, l—subterminal pollex tooth, m—terminal tooth, n—proximal dactyl tooth, o—oblique ridge, p—carinal tooth, q—bristles of upper carpal cavity, r—pollex‐gap line of tubercles, and s—“saw‐tooth” dentations of minor chela.

***Uca intermedia*** von Prahl and Toro, 1985 (Figure 4A–E). Small to moderate‐sized species. Up to 18 mm in carapace width. Frontal region (Figure 4A.a,D.a) about 8% of carapace width. Upper orbital margin smooth, raised, sinusoid‐shaped line (Figure 4A.b). Frontal margin ending in small, outward‐pointing antero‐lateral angle (Figure 4A.c). Anterior lateral margins short, forming an obtuse angle with the beaded dorsolateral margin. Eyestalks long, without stylets in males but with short stylets in females. Very small eyebrows. Lower orbital margin formed by small, oblong tubercles (Figure 4D.d). Carapace length 64% of carapace width (Figure 4A), surface smooth. H‐depression is clearly defined, with light pubescence; hepatic and branchial regions inflated. Dorsolateral margin long (Figure 4D.e). No striae. Manus much longer than the pollex and dactyl (Figure 4B) which are flattened. Manus surface covered uniformly with large, distinct tubercles. Posterior portion of dorsal manus with a band of large bristles (Figure 4A.f,C.f). Submarginal line of tubercles on lower edge of manus extending almost to the tip of pollex (Figure 4B.g). Pubescence forming a large oblong patch at the terminus (Figure 4B.h). Line of large tubercles forming the ventral keel of manus and pollex (Figure 4B.i,C.i). Distinct row of large tubercles on pollex edge of gape become smaller toward the terminus (Figure 4B.j,C.j). Proximal pollex heavy, with large bristles (Figure 4B.k,C.k); dorsal surface of pollex with distinct tubercle teeth in gape. Terminus a single sharp tooth (Figure 4B.m,C.m). Outer surface of dactyl with large tubercles proximally (Figure 4B.n). Dactyl gape edge with large tubercles, largest proximal to the largest tooth on pollex (Figure 4B.l,C.l). Tip of dactyl sharp and downwardly pointing. Inner surface of pollex and dactyl smooth and shiny (Figure 4C). Oblique ridge not distinct (Figure 4C.o). Single large tubercle on anterior edge of lower carpal cavity (Figure 4C.p), no carina lining the anterior edge of carpal cavity. Roof of carpal cavity with heavy setae bristles (Figure 4C.q). Propodus‐dactyl articulation prominent; row of large tubercles following the gape from articulation junction to near the tip of pollex (Figure 4C.r). Row of smaller tubercles extends to the dactyl terminus. Minor cheliped with very large, distinctly “saw‐tooth” teeth in gape (Figure 4D.s). Merus and propodus segments of walking legs smooth, no pubescence (Figure 4E). Merus moderately wide compared to length (43%); numerous short setae, little pubescence. Type location: Punta Soldado, Buenaventura Bay, Colombia. Previous range: Panama to Colombia, new to Ecuador.

**FIGURE 5:**
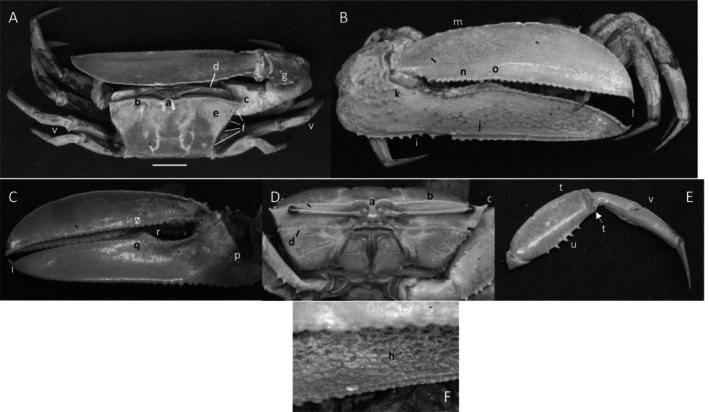
*Uca ornata* (Smith, 1870) (USNM 138615), Panama, Gulf of Panama, Panama Bay, Panama Viejo. (A) Dorsal view of male. Bar = 10 mm scale. (B) Front view. (C) Inner cheliped. (D) Ocular view. (E) Third ambulatory. (F) Setose pits on outer pollex. a—frontal region, b—anterior margin, c—anterior‐lateral angle, d—suborbital margin, e—venation, f—lateral margin spines, g‐ knobs on manus, h—setose pits, i—keel, j—row of tubercles, k—tubercles before articulation junction, l‐ terminal spines of dactyl and pollex, m—dorsal ridge of tubercles, n‐ row of teeth on lower dactyl, o—pit, p—large tubercle on edge of carpal cavity, q—line of tubercles on pollex, r—pollex ridge, s—line of dactyl teeth, t—pubescence and setae on merus, u—ventral merus spines, and v—carpal and propodal pubescence.

***Uca ornata*** (Smith, 1870) (Figure 5A–F) (USNM 138615). Large species, up to 50 mm in carapace width. Frontal region (Figure 5A.a,D.a) approximately 6% of carapace width. Upper orbital margin smooth but slightly beaded in appearance (Figure 5A.b,D.b). Frontal margin ending with a short, sharp spine at the anterolateral angle (Figure 5A.c,D.c). Anterior lateral margins diverging. Eyestalks long without terminal stylet. Little or no eyebrow. Lower orbital margin with low rectangular dentation (Figure 5A.d,D.d). Carapace length 65% of width (Figure 5A); surface shiny but finely granular. Area of branchial chamber etched with “dandelion‐shaped” venation originating in the posterior H‐depression sulcus (Figure 5A.e). Sutures of H‐depression with pubescence. Antero‐ and posterio‐lateral margins ornamented with large rounded spines (Figure 5A.f); clear gap between the most anterior and first lateral spine. A line of eight spines defining the mid‐ and posterior lateral line (Figure 5A.f). Posterio‐dorsal margin a smooth line, curving upward on the lateral carapace and becoming beaded (Figure 3F.h). Pollex comprises 67%–70% of propodus length on the major cheliped. Pollex‐dactyl gap small (Figure 5B). Dorsal manus margin with 2–3 knob‐like tubercles and edge with profuse setae (Figure 5A.g). Outer manus face with large, widely spaced tubercles and considerable pubescence (Figure 5B). Textural junction between manus and pollex abrupt. External pollex covered with circular depressions filled with short bristles (Figure 5F.h). Ventral keel with large evenly spaced tubercles extending from the manus base to the mid pollex (Figure 5B.i); distal half of keel with small closely aligned tubercles. A second, sub‐ventral row of tubercles running parallel to the keel (Figure 5B.j). Dactyl articulation almost horizontal with a row of 2–3 large tubercles (Figure 5B.k). Evenly spaced large tubercles lining the proximal pollex gap. Distal tubercles becoming very small. Distal ends of pollex and dactyl may overlap. Terminus of pollex and dactyl with large spines (Figure 5B.l,C.l). Dorsal dactyl edge beginning with a line of evenly spaced tubercles (Figure 5B.m) diminishing in size after 25% of length. Outward surface of dactyl smooth, shiny with fine texture. Lower edge of dactyl on gap with large, jagged tubercles (Figure 5B.n). Circular mid‐dactyl depression above the gap (Figure 5B.o). Inner manus generally smooth. Smooth sulcus at the pollex‐manus junction. Oblique ridge prominent with a single larger tubercle on the edge of the carpal cavity (Figure 5C.p). Distal end ending in a patch of small tubercles becoming part of the ventral keel. Tubercles lining the carpal cavity indistinct. Upper edge of pollex with a strong, row of tubercles (Figure 5C.q) extending to the terminus. Inner articulation junction with a single dorsal spine. Large central ridge on the proximal pollex (Figure 5C.r). Inner surface of dactyl smooth; lower dactyl edge with a row of large tubercles lining the gap (Figure 5C.s). Merus of walking legs much longer than wide (Figure 5E); pubescence on the dorsal margin (Figure 5E.t); distal lower merus edge lined with a short row of pile (Figure 5E.t). Ventral merus with 1–5 spines (Figure 5E.u). Dorsal carpus and propodus with pubescence (Figure 5A.v,E.v). Type location: West coast of Central America. Range: El Salvador to northern Peru. Remarks: von Hagen (1968) described this species in Peru as *Uca pizarri*

**FIGURE 6:**
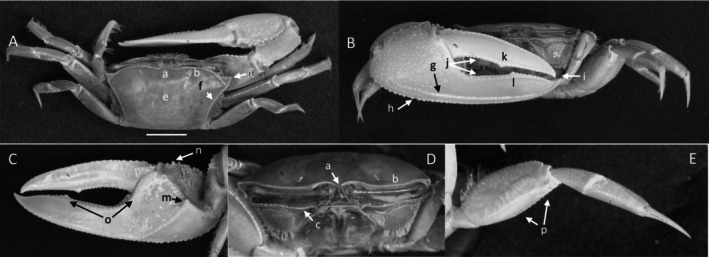
*Uca princeps* (Smith, 1870) (UNI 757) collected Puerto Sitio Nueva, SEL. (A) Dorsal view of male. Bar = 10 mm scale. (B) Front view. (C) Inner cheliped. (D) Ocular view. (E) Third ambulatory. a—interocular space, b—superior orbital margin, c—suborbital dentations, d—antero‐lateral angle, e—depression, f—posterior lateral margin, g—subpollex groove, h—keel of pollex, i—terminal tooth, j—tubercle line of gap, k—dactyl tooth, l—pollex tooth, m—proximal tooth of oblique ridge, n—spine of upper carpal carina, o—inner tubercle line of manus and pollex, and p—small vental spines on merus.

***Uca princeps*** (Smith, 1870) (Figure 6A–E). Large species. Carapace width may exceed 40 mm. Frontal region (Figure 6A.a,D.a) about 5% of carapace width. Upper orbital margin (Figure 6A.b,D.b) curving smoothly to antero‐lateral angle (Figure 6A.d). No eyebrow. Eyestalks are usually without a stylet in adults. Lower orbital with large, square dentations (Figure 6D.c). Carapace length is about 59% of its width (Figure 6A); surface beaded or granulate. Anterior and lateral carapace margins intersect at a sharp point (Figure 6A.d). H‐depression shallow (Figure 6A.e). Lateral margins are beaded, formed from a continuous row of small tubercles (Figure 6A.f). Pollex and dactyl appear long (Figure 6B). Manus is about one third of pollex and dactyl length (Figure 6B,C). Large tubercles are on the dorsal surface of manus, transitioning to a small field on the ventral surface. A submarginal row of tubercles on the pollex extends to the manus and to the end of the pollex; a groove with pubescence is present (Figure 6B.g). A row of tubercle beads extends onto the keel of the pollex (Figure 6B.h). Fingers appear very long with a smooth outer surface. Dactyl and pollex of the major cheliped form a distinct proximal gap when closed (Figure 6B,C). Gap is approximately equal to the height of the dactyl base; gap is lined with a row of tubercles following the margin on both pollex and dactyl (Figure 6B.j). Tips of both dactyl and pollex are sharp and hooked (Figure 6B.i). Dactyl has larger tubercles about one third of the distance from the terminus (Figure 6B.k). Pollex has a large, triangular tooth in a similar position (Figure 6B.l). An oblique ridge on the palm extends to the carpal cavity and ends with a large tooth (Figure 6C.m). Tubercles continue to follow the carpal edge upward, forming a marginal carina; the upper margin of the cavity (Figure 5C.n) formed by a carina of large tubercles and long pubescence. The inner surface of the manus, dactyl, and pollex is smooth. A prominent row of tubercles lines the gap from the articulation cuff to the distal end of the pollex (Figure 6C.o). Posterior ventral merus on the third ambulatory has moderate‐sized tubercles (Figure 6E.p). Type location: Corinto, Nicaragua. Range: Southern California, USA to Peru.

**FIGURE 7:**
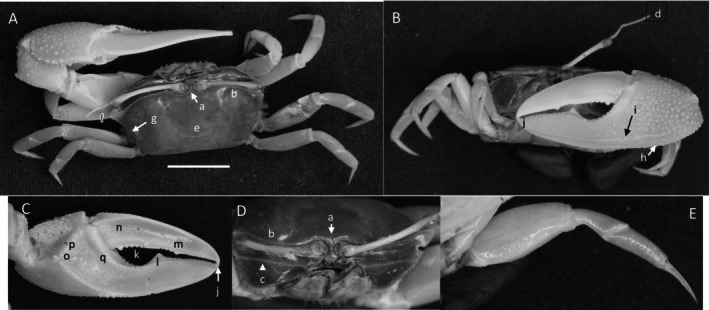
*Uca stilifera* (H. Milne Edwards, 1852) (UNI 742) collected Bahia de Caráquez, MAN. (A) Dorsal view of male. Bar = 10 mm scale. (B) Front view. (C) Inner cheliped. (D) Ocular view. (E) Third ambulatory. a—interocular space, b—supraorbital margin, c—lower orbital margin, d—stylet, e—H‐depression, f—anterolateral angle, g—lateral margin, h—keel, i—submarginal groove, j—terminal dactyl and pollex spines, k—proximal gap, l—triangular pollex tooth, m—dactyl tooth, n—inner dactyl sulcus, o—tooth of oblique ridge, p—carina margin, and q—pollex tubracle line.

***Uca stylifera*** (H. Milne Edwards, 1852) (Figure 7A–E). Large species. Carapace width up to 30 mm. Frontal region (Figure 7A.a,D.a) about 10% of carapace width. Upper orbital margin smooth, curved, and swollen (Figure 7A.b,D.b). No eyebrow. Suborbital margin with large, square dentation (Figure 7D.c). Eyestalk near major cheliped with long thread‐like stylet in males (Figure 7B.d). Carapace length about 58% width, surface smooth with no pubescence. Central H‐depression (Figure 7A.e) shallow, and cardio‐branchial regions swollen. Antero‐lateral angle (Figure 7A.f) pointing outward. Anterior lateral margin smooth and non‐distinct, posteriorly developing into a line of tubercles (Figure 7A.g). Manus slightly shorter than pollex and dactyl (Figure 7B,C); outer manus with numerous large tubercles throughout. Keel of large tubercles on lower margin of manus (Figure 7B.h). Manus submarginal line of tubercles and groove extending to pollex tip (Figure 7B.i). No pubescence in groove. Pollex and dactyl compressed and blade‐like. Dactyl and pollex terminus forming small pointed hooks (Figure 7C.j), moderate‐sized tubercles on outer surface. Base of pollex large where it merges with manus. Proximal gap (Figure 7C.k) forming “gash” under dactyl‐manus articulating junction occupying proximal one fourth of pollex, distal end of “gash” with large triangular tooth (Figure 7C.l). Distal pollex and dactyl forming a small gap. Distal pollex lined with large tubercles becoming progressively smaller toward terminus. Distal dactyl tooth (Figure 7C.m) is closer to terminus than triangular pollex tooth. Inner surface is smooth and deeply excavated (Figure 7C.n). Oblique ridge on palm of manus is well defined, large spine at intersection with carpal cavity (Figure 7C.o). Carina of large tubercles lines carpal cavity (Figure 7C.p). Line of tubercles following dactyl articulating cuff to distal terminus of gap (Figure 7C.q); area bound by ridges forming a sulcus. Third ambulatory (Figure 7E) without setae or significant pubescence. Type location: Guayaquil, Ecuador. Range: Honduras to northern Peru.

Subfamily Gelasiminae Miers, 1886

Genus *Petruca* Shih, Ng and Christy, 2015

One species in genus. Moderately sized, carapace width up to 20 mm.

**FIGURE 8:**
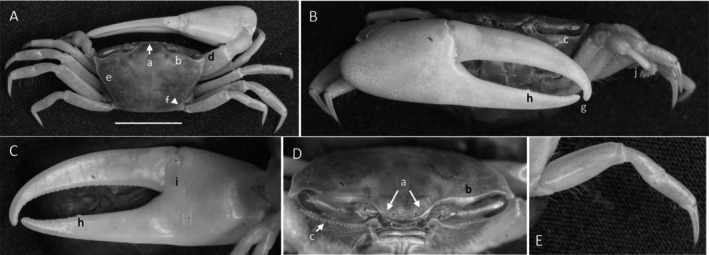
*Petruca panamensis* (Stimpson, 1859) (UNI 752) collected Playa Camerones, ESM. (A) Dorsal view of male. Bar = 10 mm scale. (B) Front view. (C) Inner cheliped. (D) Ocular view. (E) Third ambulatory. a—frontal region, b—upper margin of orbital and eyebrow, c—dentation on suborbital margin, d—anterolateral angle, e—lateral line, f—posterior stria, g—hooked tip of dactyl and pollex, h—dactyl tooth, i—articulation ridge, and j—dense setae.

***Petruca panamensis*** (Stimpson, 1859) (Figure 8A–E). Moderate‐sized species. Carapace width up to 20 mm. Frontal broad (Figure 8A.a,D.a) about 27% of carapace width. Eyebrow visible, about 30% of eyestalk width. Upper orbital margin (Figure 8A.b,D.b) angled posteriorly, with granular margin smooth and sinusoid. Suborbital margin with large conical dentations (Figure 8B.c,D.c). Antero‐lateral angle is sharp and turned outward (Figure 8A.d). Carapace length (Figure 8A) about 67% of carapace length; dorsal carapace surface almost flat and rough, appearing pitted when dry. Lateral line (Figure 8A.e) finely granular, extending about three fourth carapace length. Beyond the posterior terminus of the lateral line, two separated granular striae follow (Figure 8A.f). Manus about equal in length to pollex and dactyl (Figure 8B,C). Surface of manus, pollex and dactyl finely granulate. Manus and pollex without ventral keel. Gap wide, extending from dactyl articulation to tip (Figure 8B.g) which overhangs slightly. A few tubercles are in the gap. Pollex with single large tubercle positioned before tip (Figure 8B.h,C.h). Inner surface (Figure 8C) of manus, dactyl and pollex smooth, with no oblique ridge or carpal carina. Pre‐dactyl ridge of tubercles are poorly developed (Figure 8C.i); articulation cuff almost smooth. Inner central manus convex. Minor cheliped profuse distally with setae (Figure 8B.j). Ambulatories smooth with a few long setae (Figure 8E). Male abdominal segments (pleonites) free. Pleonal locking mechanism absent. Type location: Panamá (neotype—Culebra, Panamá). Range: Honduras to northern Peru.

Genus *Minuca* Bott, 1954

Five species, widely ranging in size. The frontal region is usually more than 25% of carapace width. The carapace is more trapezoid‐shaped (truncated hip) than rectangular; some species have a pentagon shape. In lateral view, the carapace never appears cylindrical. Eyestalks are short. Suborbital dentations are usually small. The anterolateral margin is long, curving into the dorsolateral surface; the carapace has two posterolateral striae. All pleonites are free. The major pollex may possess a ventral carina.

**FIGURE 9:**
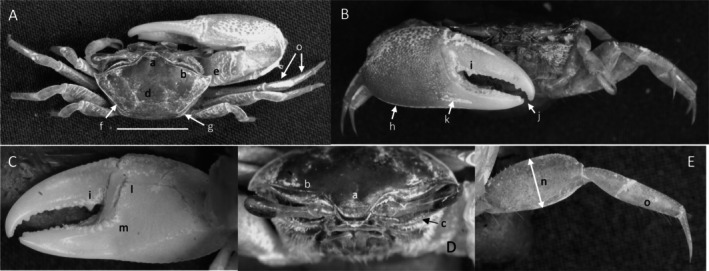
*Minuca argillicola* (Crane, 1941) (UNI 828) collected Rio Agua Clara, Daule, ESM. (A) Dorsal view of male. Bar = 10 mm scale. (B) Front view. (C) Inner cheliped. (D) Ocular view. (E) Third ambulatory. a—frontal region, b—upper orbital margin, c—lower orbital dentations, d—H‐depression, e‐ anterolateral angle, f—long lateral margin, g—inferior stria, h—manus keel, i—proximal dactyl tooth, j—dactyl and pollex terminus, k—triangular sulcus with pubescence, l—pre‐dactyl articulation, m—inner pollex tubercle line, n—width ambulatory merus, and o—pubescence.

***Minuca argillicola*** (Crane, 1941) (Figure 9A–E). Small crab. Maximum carapace width about 12 mm. Frontal region about 23% carapace width (Figure 9A.a,D.a). Orbitals strongly oblique, giving the carapace a penta‐form appearance (Figure 9A.b,D.b). Margin smooth. Eyebrows very wide, almost equal in thickness to the eyestalk (Figure 9A.b,C.b). Suborbital margin with moderate‐sized square dentations (Figure 9D.c). Carapace length about 66% width (Figure 9A); surface granular with patches of pubescence in lateral sutures of H‐depression (Figure 9A.d). Anterolateral angle pointing outward (Figure 9A.e). Two lateral margins, one connects to the antero‐lateral angle, the second more ventral; dorsal lateral line terminates about half the length of the carapace and curves inward (Figure 9A.f); the second line terminates in the posterior quarter of carapace, curving toward the midline (Figure 9A.g). Length of pollex and dactyl on major cheliped clearly short compared to the manus (Figure 9B). Dorsal manus with large tubercles and light pubescence. Outer manus with moderate‐sized tubercles. Ventral keel (Figure 9C.h) formed with prominent large tubercles extending from the posterio‐ventral manus to the base of the pollex. Dactyl with prominent tooth near articulation (Figure 9B.i,C.i); terminus curving down (Figure 9B.j). Pollex with triangular depression near manus junction, filled with pile (Figure 9B.k). Distal pollex tip is blunt (Figure 9B.j,C.j). Gap smaller than width of mid‐length pollex or dactyl. Inner surface of manus convex without oblique ridge and no carina around lower carpal cavity. Weakly developed pre‐dactyl line of tubercles covered with pile (Figure 9C.l). Line of 6–7 large tubercles on inner pollex follows gap (Figure 9C.m). Ambulatory merus segment dorsally serrated and wide, 40% length (Figure 9E.n). Pubescence on ambulatory carpus and propodus (Figure 9A.o,E.o). Type location: Golfito, Costa Rica. Range: Costa Rica to Ecuador.

**FIGURE 10:**
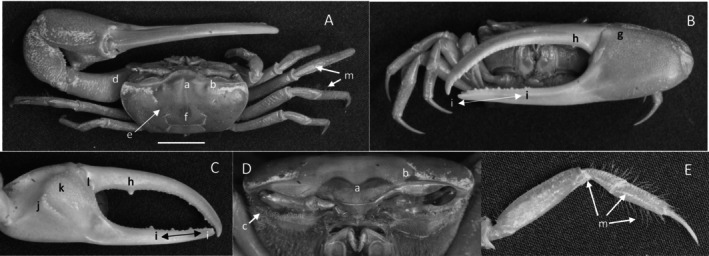
*Minuca brevifrons* (Stimpson, 1860) (UNI 809) collected Rio Agua Clara, Daule, ESM. (A) Dorsal view of male. Bar = 10 mm scale. (B) Front view. (C) Inner cheliped. (D) Ocular view. (E) Third ambulatory. a—frontal region, b—upper frontal margin, c—suborbital dentations, d—anterolateral angle, e—circular pubescent tufts, f—H‐depression, g—upper manus, h—proximal dactyl tooth, i—dactyl and pollex terminus, j—oblique ridge apex, k—tubracle field, l—predactyl articulation ridge, and m—pubescence of carpus and propodus.

***Minuca brevifrons*** (Stimpson, 1860) (Figure 10A–E). Large species. Carapace width is up to 35 mm. Frontal portion (Figure 10A.a,D.a) about 33% carapace width. Upper orbital smooth (Figure 10A.b,C.b) with some pubescence behind the margin. Eyebrows large, equal to eyestalk width. Small conical dentations on suborbital margin (Figure 10D.c). Anterolateral angle not sharp (Figure 10A.d), curving toward the midline. Lateral margin curved and converging to the midline. Carapace width about 66% length (Figure 10A), surface smooth but covered with small circular tufts of pubescence (Figure 10A.e) giving the carapace a finely “polka‐dotted” appearance. H‐depression (Figure 10A.f) very prominent in the cardiac region of the carapace. Pubescence on carapace sparse. Dactyl and pollex appear thin and tubular, longer than manus (Figure 10B). Moderate‐sized tubercles on the surface of upper manus (Figure 10B.g). Outer surface of pollex and dactyl smooth, gap much larger than width of dactyl or pollex (10B), a few tubercles in the gap. On dactyl, largest tubercle proximal (Figure 10B.h,C.h) with other prominent tubercles at the distal end. Terminus curved down. Pollex with large tubercle mid‐length and terminus trifurcated (Figure 10B.i,C.i). Inner pollex and dactyl surface smooth. Oblique ridge from pollex base to carpal cavity proximately tuberculate to a high apex (Figure 10C.j) but smooth distally, dorsal edge of carpal cavity amorphous. No carina around carpal cavity. Center of palm with a field of tubercles, ventral portion smooth (Figure 10C.k). Prominent pre‐dactyl ridge of tubercles curving away from the articulation forming a y‐shaped arrangement of large evenly spaced tubercles (Figure 10C.l). Merus much greater in length than width. Carpus and propodus with numerous long setae and significant pubescence (Figure 10A.m,E.m). Type location: Playa Todos Santos, Cabo San Lucas, Baja California Sur, Mexico. Range: Baja California Sur, Mexico to Ecuador. Remarks: During this study, *M. brevifrons* in Ecuador was corroborated by Ramos‐Veliz, Vergara, and Jorge (2022).

**FIGURE 11:**
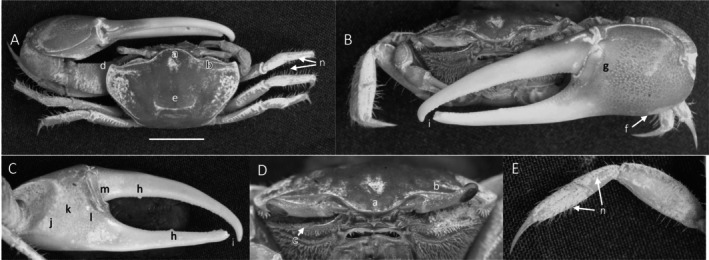
*Minuca ecuadoriensis* (Maccagno, 1928) (UNI 811) collected Cayapas‐Mataje, La Tola, ESM. (A) Dorsal view of male. Bar = 10 mm scale. (B) Front view. (C) Inner cheliped. (D) Ocular view. (E) Third ambulatory. a—frontal region, b—upper orbital margin, c—suborbital margin dentations, d—antero‐lateral angle, e—H‐depression, f—keel pubescence, g—pre‐articuation groove, h—tooth on dactyl and pollex, i—terminus of dactyl and pollex, j—apex of oblique ridge, k—tubracle field, l—predactyl ridge, m—tubercles on articulating cuff, and n—setae and pubescence on merus and propodus of ambulatory.

***Minuca ecuadoriensis*** (Maccagno, 1928) (Figure 11A–E). Large species. Carapace width is up to 25 mm. Frontal region (Figure 11A.a,D.a) is very broad, about 33% of carapace width. Upper orbital margins (Figure 11A.b,C.b) slightly angled from the frontal region. Eyebrows visible, with setae, and about the same width as the eyestalk. Suborbital margin without dentations (Figure 11D.c). Anterolateral angle curving toward the midline (Figure 11A.d). Carapace length about 65% of width (Figure 11A). Carapace surface rugose with numerous pits and large, dispersed tufts of pile (Figure 11A). H‐depression deep with pubescence (Figure 10A.e). Manus approximately equal in length to the pollex and dactyl (Figure 11B,C), upper surface of the exterior manus with moderate‐sized tubercles decreasing in diameter ventrally. Tubercle keel on the posterio‐ventral manus with pubescence (Figure 11B.f). Pubescence between the manus and articulating cuff (Figure 11B.g). Pre‐dactyl row of widely dispersed tubercles. Articulation cuff with 4–5 prominent tubercles. Dactyl and pollex smooth. Large gap between dactyl and propodus (pollex) when fingers are closed. Dactyl and pollex each with a central large tubercle but pollex slightly more distal (Figure 11C.h). Terminus of pollex trifurcated, dactyl hooked downward (Figure 11B.i,C.i). Inner surface of manus with an undeveloped oblique ridge, apex at the medial edge of the carpal cavity (Figure 11C.j) consisting of a group of 5–6 larger tubercles. Field of tubercles on the upper half of the inner manus (Figure 11C.k). Pre‐dactyl articulation curving ridge of large tubercles (Figure 11C.l). Articulation cuff with a short row of several large tubercles (Figure 11C.m). Ambulatories (Figure 11A.n,E.n) profuse with setae and pubescence. Type location: Esmeraldas, Ecuador. Range: Sonora, Mexico to northern Peru. Remarks: *Minuca* aff. *ecuadoriensis* is similar morphologically to *M. ecuadoriensis* (Maccagno, 1928) except that it has a deep red‐brown color as noted by Crane (1975, 164). Von Hagen (1968) described *U. lanigera* from Puerto Pizarro (Peru) and Guayaquil (Ecuador) as also having a “red violet to brown” colored carapace. COI sequences of specimens from Puente Sitio Nuevo, Palmar, St. Elena and those from Isla Santay, Rio Guayas, Guayaquil, Guayas belong to two different clades (Figure 29). Because the type locality of *Minuca ecuadoriensis* (Maccagno, 1928; Crane 1975) is close to Palmar, the crabs are treated as a putative distinct species which needs further confirmation.

**FIGURE 12:**
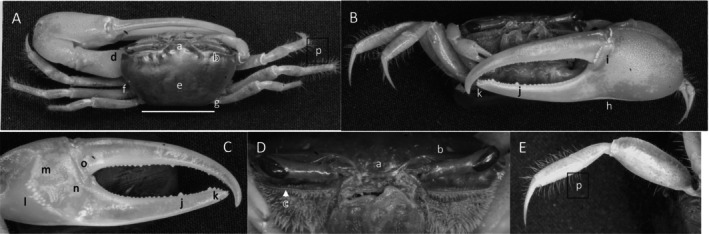
*Minuca galapagenesis* (Rathbun, 1902) (UNI 840) collected Bahia de Tortuga, Santa Cruz, GAL. (A) Dorsal view of male. Bar = 10 mm scale. (B) Front view. (C) Inner cheliped. (D) Ocular view. (E) Third ambulatory. a—frontal, b—upper orbital, c—lower orbital dentations, d—anterolatertal angle, e—H‐depression, f—lateral margin, g—posterior stria, h—keel, i—tubercle line on cuff, j—large tooth, k—dactyl and pollex terminus, l—apex of oblique ridge, m—tubercle field, n—predactyl tubercle line, o—tubercle line on inner articulation cuff, and p—carpal setae.

***Minuca galapagensis*** (Rathbun, 1902) (Figure 12A–E). Moderate‐size species. Carapace up to 22 mm. Frontal (Figure 12A.a,D.a) moderately wide, 28% of carapace width. Orbital (Figure 12A.b,D.b) not strongly angled. Eyebrows wide, equal to eyestalk width. Upper orbital margin smooth. Suborbital dentations (Figure 12D.c) moderately sized and square. Anterolateral angle (Figure 12A.d) blunt, point straight ahead or slightly toward the midline. Carapace length about 66% of width (Figure 12A); trapezoid‐shaped without pile or pubescence. Cardiac region convex, H‐depression deep (Figure 12A.e). Lateral margin (Figure 12A.f) gently curving to the posterior. Second, shorter lateral stria more posterior (Figure 12A.g). A third stria is on the lateral surface of the brachial chamber. Manus slightly shorter than the dactyl and pollex in adults (Figure 12B). External face of manus rugose. Tuberculate ventral keel (Figure 12B.h). Line of tubercles on articulation cuff (Figure 12B.i). Dactyl junction with coarse setae. Pollex and dactyl thin and tubular with very small tubercles. Gap larger than width of dactyl or pollex; moderate‐size teeth larger on pollex than dactyl (Figure 12B.j,C.j). Pollex terminus trifurcated (Figure 12B.k,C.k). Inner manus surface with oblique ridge formed of fused, oblong tubercles terminating in a high apex (Figure 12C.l) at the carpal cavity. Anterior and dorsal edge of carpal cavity lined with tubercles. Central portion of manus very convex with a field of small tubercles (Figure 12C.m). Pre‐dactyl ridge (Figure 12C.n) starting near the upper edge of articulation cuff and extending downward onto the pollex. Row of 4–5 tubercles on cuff (Figure 12C.o). Inner surface of dactyl and pollex smooth and shiny. Setae profuse on ambulatory carpus (Figure 12E.p). Type location: Santa Cruz (Indefatigable) Island, Galápagos Archipelago. Range: Gulf of Nicoya, Costa Rica to southern Peru. Remarks: This species is difficult to distinguish from *Minuca herradurensis* (Bott, 1954). Interestingly, Crane (1975) avoided a clear comparison of the two.

**FIGURE 13:**
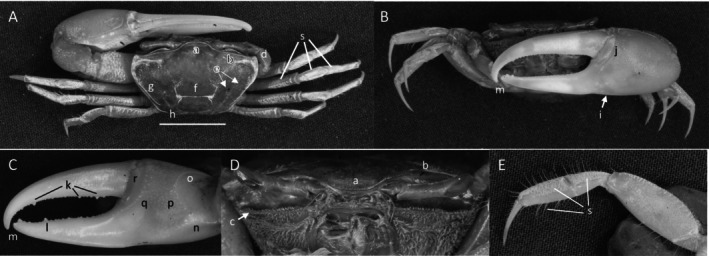
*Minuca osa* (Landstorfer and Schubart, 2010) (UNI 820) collected Cayapas Mataje, La Tola, ESM. (A) Dorsal view of male. Bar = 10 mm scale. (B) Front view. (C) Inner cheliped. (D) Ocular view. (E) Third ambulatory. a—frontal region, b—upper orbital margin, c—suborbital margin dentations, d—anterolateral angle, e—circular tufts of setae, f—H‐depression, g—lateral line, h—posterior stria, i—keel of manus, j—pre‐dactyl groove and cuff, k—dactyl teeth, l—teeth on pollex, m—dactyl and pollex terminus, n—oblique ridge apex, o—carina of upper carpal cavity, p—tubercle field, q—pre‐dactyl row of tubercles, r—tubercles on articulation cuff, s—pubescence and setae on merus, carpus and propodus of third ambulatory.

***Minuca osa*** (Landstorfer and Schubart, 2010) (Figure 13A–E). Moderate‐size species. Carapace width up to 21 mm. Frontal region (Figure 13A.a,D.a) about 35% of carapace width. Upper orbital margin (Figure 13A.b,D.b) smooth but laterally tubercles large and fused. Sulcus behind orbital with thin layer of pile. Eyebrows visible but < 50% of eyestalk. Suborbital dentations are small (Figure 13D.c). Antero‐lateral margins curving inward (Figure 13A.d). Carapace length about 63% width (Figure 13A) carapace surface granular with pile‐filled pores when dried (Figure 13A.e) giving it a “polka‐dotted” appearance. H‐depression (Figure 13A.f) with lateral patches of pubescence. Lateral margin (Figure 13A.g) curving, extending to posterior carapace. A second lateral striae (Figure 13A.h) parallel lateral margin. Outer manus surface (Figure 13B) covered with moderate to small tubercles. Ventral keel on manus (Figure 13B.i) with patches of pile along length. Six to eight tubercles on articulation cuff (Figure 13B.j). Dactyl and pollex about same length as manus (Figure 13B,C). Dactyl and pollex flat, more blade‐like than tubular. Gap wide as pollex or dactyl. Small teeth in gap. Proximal dactyl (Figure 13C.k) with 3–5 tubercles. Distal with one large tubercles. Pollex (Figure 13C.l) with tubercle slightly distal of mid‐length. Terminus trifurcated (Figure 13B.m,C.m). Inner surface of dactyl and pollex appearing rough. Inner manus with oblique ridge terminating in carpal cavity (Figure 13C.n). Distal end of oblique ridge weak, proximal with cluster of 5–6 tubercles on lower edge of carpal cavity. Dorsal cavity (Figure 13C.o) with carina extending downward to a tubercle field (Figure 13C.p) on central manus. Tubercles from lower and upper edge of carpal cavity may not meet, no setae or pubescence present. Pre‐dactyl articulation area (Figure 13C.q) with an arching line of large tubercles extending to pollex base. Articulation cuff (Figure 13C.r) with 5–7 large tubercles on lower portion. Dorsal surface of ambulatory (Figure 13A.s,E.s) merus, carpus and propodus profuse with pile and numerous long setae. Type location: Golfo Dolce, Osa peninsula, Costa Rica. Previous range: From Osa peninsula, Costa Rica to Montig Gulf, Panama, new to Ecuador.

Genus *Leptuca* Bott, 1973

Fifteen described species. Small to moderate‐sized crabs. Frontal region 25%–30% of carapace width. Carapace usually arched to semi‐cylindrical in shape when viewed laterally. Suborbital dentations are definite. Dactyl and pollex usually much longer than the manus of the cheliped. Inner surface of manus may have an oblique ridge. Carpal cavity usually with a beaded edge or carina. Anterolateral margin short; carapace with up to two posterolateral striae. Pleonites are either free or somites 4–6 are partly to completely fused. Major pollex often without a ventral carina or keel.

**FIGURE 14:**
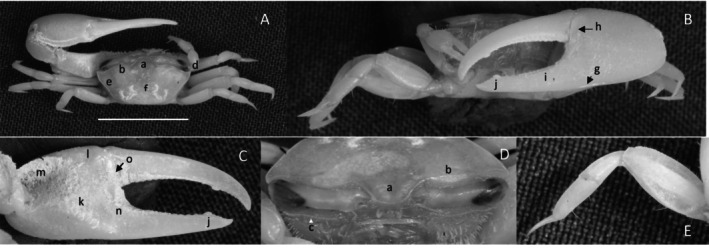
*Leptuca batuenta* (Crane, 1941) (UNI 866) collected Portete, Mompeche, ESM. (A) Dorsal view of male. Bar = 10 mm scale. (B) Front view. (C) Inner cheliped. (D) Ocular view. (E) Third ambulatory. a—frontal region, b—upper orbital margin, c—suborbital margin, d—anterolateral angle, e—lateral margin, f—H‐depression, g—submanus keel, h—dactyl articulating cuff, i—tubercle lining of pollex, j—terminal tooth of pollex, k—apex of oblique ridge, l—superior tubercle line of carpal cavity, m–pubescence on upper carpal cavity, n—tubercle line on pollex, o—tubercle line of dactyl articulation cuff.

***Leptuca batuenta*** (Crane, 1941) (Figure 14A–E) Small species. Carapace width up to 8 mm. Frontal region moderately wide (Figure 14A.a,D.a), about 26% carapace width. Upper orbital margin (Figure 14A.b,D.b) sinuous, slightly oblique. Eyebrow less than one third eyestalk width. Suborbital dentations (Figure 14D.c) weak, forming lateral crest. Anterolateral angle (Figure 14A.d) sharp and pointed outward. Anterior lateral margins straight (Figure 14A.e) curving inward in posterior. Posterior striae short. Carapace length about 51% of width (Figure 14A), surface smooth with profuse pubescence in H‐depression (Figure 14A.f), appearing semicircular from lateral view. Upper manus of cheliped with moderate‐sized tubercles (Figure 14B), ventral manus with short keel (Figure 14B.g) of distinct tubercles near base but decreasing in size toward pollex. Articulation cuff distinct (Figure 14B.h). Blade‐like dactyl and pollex longer than manus (Figure 14B,C). Pollex with broad, depressed base. Proximal pollex with pile. Gap twice width of pollex. Tubercles on exterior follow gap from articulation cuff to distal end of pollex (Figure 14B.i). Single, large, triangular tubercle on distal quarter of pollex (Figure 14B.j,C.j), tip hooked and simple. Distal end of pollex between tooth and tip is remarkable, almost “Symitar”‐shaped. Inner surface of dactyl and manus smooth. Central manus raised creating sulcus at base of pollex. Oblique ridge poorly developed distally (Figure 14C.k). High apex at carpal cavity rim with cluster of four or five large tubercles. Weak tubercle line follows anterior edge up to dorsal rim of carpal cavity; tubercle line then bends toward dactyl base (Figure 14C.l). Dorsal carpal cavity with profuse pubescence (Figure 14C.m). Inner articulation cuff weak. Predactyl line of tubercles follows edge of cuff on to pollex (Figure 14C.n). Three to four weak tubercles on articulation cuff (Figure 14C.o). Ambulatories (Figure 14E) with few setae and very little pile. Type location: La Boca, Balboa Island, Panama. Range: El Salvador to northern Peru.

**FIGURE 15:**
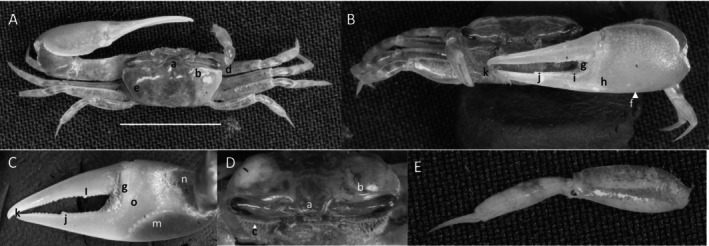
*Leptuca beebei* (Crane, 1941) (UNI 793) collected Estero Salado, Posorja, GUA. (A) Dorsal view of male. Bar = 10 mm scale. (B) Front view. (C) Inner cheliped. (D) Ocular view. (E) Third ambulatory. a—Frontal region, b—upper orbital margin, c—lower orbital dentations, d—antero‐lateral angle, e—posterior‐lateral margin, f—inferior manus keel, g—pubescence of articulation joint, h—manus‐pollex depression, i—line of tubercles following gap, j—dactyl medial tooth, k—dactyl/pollex terminus, l—proximal dactyl tooth, m—oblique ridge, n—pubescence and carina of upper carpal cavity, and o—tubercle line from manus to pollex.

***Leptuca beebei*** (Crane, 1941) (Figure 15A–E). Small species. Carapace width up to 13 mm. Frontal region (Figure 15A.a,D.a) squared, forming about 29% of carapace width. Upper orbital margin (Figure 15A.b,D,b) curving to lateral line. Eyebrow broad equal to width of eyestalk. Suborbital margin (Figure 15D.c) with small square dentations. Anterolateral angle (Figure 15A.d) sharp pointing forward. Anterior lateral margins parallel. Carapace length about 59% width (Figure 15A), surface smooth, shiny, and semi‐cylindrical (Figure 15A) in lateral view. H‐depression shallow. Posterior lateral margins long (Figure 15A.e) curving inward. Dactyl and pollex smooth and much longer than manus (Figure 15B,C). Manus with small tubercles; ventral keel (Figure 15B.f) tuberculate terminating at base of pollex. Large gap between pollex and dactyl. Dactyl articulation (Figure 15B.g) with large tuft of long pile. Base of pollex depressed forming sulcus (Figure 15B.h). Line of tubercles parallel to gap (Figure 15B.i). Proximal tubercle ridge develops into large central tubercle of pollex (Figure 15B.j,C.j). Pollex terminus hooked upward slightly (Figure 15B.k,C.k). Outer surface of dactyl smooth. Large tubercle near articulation junction (Figure 15C.l). Inner surface of dactyl and pollex smooth. Oblique ridge (Figure 15C.m) arched, building to a prominent apex at edge of carpal cavity. Dorsal margin of cavity with pubescence‐covered carina (Figure 15C.n). Dorsal and ventral carpal carinae do not meet. Area anterior to carpal cavity smooth. Row of large tubercles (Figure 15C.o) starts on articulating cuff forming right‐angle turn onto base of pollex. Long pile visible at articulation (Figure 15C.g). On ambulatories pile and setae sparse to absent (Figure 15E). Type location: La Boca, Balboa Island, Panama. Range: El Salvador to northern Peru.

**FIGURE 16:**
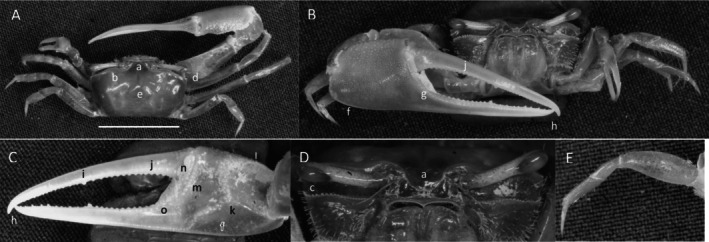
*Leptuca deichmanni* (Rathbun, 1935) (UNI 765) collected Playa Achilube, Parque Tematico Marios, ESM. (A) Dorsal view of male. Bar = 10 mm scale. (B) Front view. (C) Inner cheliped. (D) Ocular view. (E) Third ambulatory. a—frontal region, b—upper orbital margin, c—lower orbital dentations, d—antero‐lateral angle, e—H ‐depression, f—tubercle keel, g—outer pollex row, h—dactyl and pollex terminus, i—central tooth, j—tubercle line of dactyl, k—apex of oblique ridge, l—upper carpal bristles, m—first pre‐dactyl line, n—second pre‐dactyl line, and o—third pre‐dactyl tubercle row.

***Leptuca deichmanni*** (Rathbun, 1935) (Figure 16A–E). Small species. Carapace width up to 14 mm. Frontal region (Figure 16A.a,D.a) lobe‐like and 24% of carapace width. Upper orbital margin (Figure 16A.b) angled slightly. Eyebrow less than 50% of eyestalk width (Figure 16A.b). Suborbital margin with spike‐shaped dentations (Figure 16B.c,D.c). Carapace length about 64% of width (Figure 16A), surface smooth, and arched in lateral view. Anterolateral angle small (Figure 16A.d). Anterior portion of lateral line short. H‐depression (Figure 16A.e) shallow, with no pile or pubescence. Outer surface of manus with small tubercles (Figure 16B). Manus shorter than pollex or dactyl. Dactyl and pollex more blade‐like than tubular (Figure 16B,C). Gap large when fingers are closed, without pile at articulation cuff. Keel (Figure 16B.f,C.f) on manus. External surface of dactyl and pollex finely granular. Row of low teeth (Figure 16B.g) running from articulating cuff to tip of pollex. Pollex terminates with a large, hooked tubercle (Figure 16B.h,C.h). Dactyl with one large proximal tooth (Figure 16C.i), distal terminus overhanging pollex. A line of tubercles (Figure 16C.j) on proximal gap‐edge of dactyl. Inner surface of pollex and manus mostly smooth. Oblique ridge (Figure 16C.k) developing as a row of single tubercles toward carpal cavity. Upper margin of carpal cavity (Figure 16C.l) lined with setae forming short bristles. Predactyl articulation with a short row of distinct tubercles (Figure 16C.m), a second, parallel predactyl ridge (Figure 16C.n) of large tubercles extending to base of pollex. Third tubercle row on pollex following gap (Figure 16C.o). Setae sparse on ambulatories, no pubescence (Figure 16E.n). Type location: Panama. Previous range: Costa Rica to Colombia, new to Ecuador.

**FIGURE 17:**
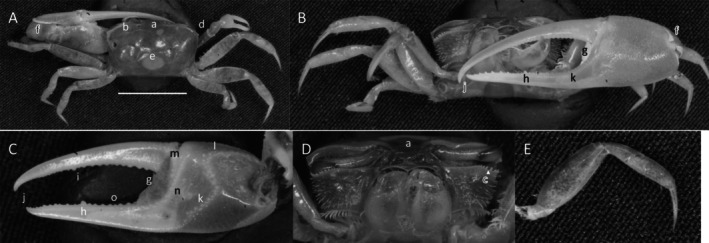
*Leptuca dorotheae* (von Hagen, 1968) (UNI 771) collected Rio Mache, San Jose de Chamanga, ESM. (A) Dorsal view of male. Bar = 10 mm scale. (B) Front view. (C) Inner cheliped. (D) Ocular view. (E) Third ambulatory. a—frontal region, b—anterior margin, c—dentations of lower orbital margin, d—antero‐lateral angle, e—H‐depression, f—posterior manus pubescence, g—dactyl junction, h—pollex tubercle, i—dactyl tooth, j—terminus, k—oblique ridge, l—dorsal carina, m—articulation tubercles, n—pre‐dactyl tubercle line, and o—tubercles on edge of pollex.

***Leptuca dorotheae*** (von Hagen, 1968) (Figure 17A–E). Small species. Carapace width up to 14 mm. Frontal region (Figure 17A.a,D.a) square to spatulate, 23% of carapace width. Anterior margin slightly angled laterally. Eyebrows (Figure 17A.b) about same width as eyestalks. Suborbital margin with dentations (Figure 17D.c), small and rectangular from midline. Laterally, dentations thin and tall, pointing outward. Antero‐lateral junction (Figure 17A.d) forming obtuse angle but sharp and pointed. No distinct posterior dorso‐lateral margin, lateral surfaces of cephalothorax concave. No posterior striae. Carapace rectangular, length 59% width (Figure 17), smooth, no pubescence, semi‐circular from lateral view. H‐depression (Figure 17A.e) obvious but no swollen regions. Dactyl and pollex (Figure 17B,C) very long compared to manus (ratio: dactyl + manus/manus = 3.3) in adult males. Outer manus covered uniformly with moderate‐sized tubercles (Figure 17B); dorsal, posterior edge of manus heavy with long pubescence (Figure 17A.f,B.f). Dactyl‐propodus junction with heavy pubescence (Figure 17B.g,C.g). Dactyl and pollex thin, tubular, gape very large. Pollex almost straight. Teeth of consistent size line gape on both pollex and dactyl. Pollex with one larger tooth slightly distal to mid‐length (Figure 17B.h). Dactyl with larger teeth proximally. One larger dactyl tooth adjacent to tooth on pollex (Figure 17B.i,C.i), distal tip curved, overlapping hooked tip of pollex (Figure 17B.j,C.j). Both dactyl and pollex smooth and glossy. Exterior of pollex with subventral crease (Figure 17B.k). Inner surface of pollex and dactyl smooth and shiny. Oblique ridge extending to apex at carpal cavity (Figure 17C.l) and then around cavity to intersect with upper carpal cavity margin; tubercles on lower oblique ridge may appear in two distinct rows. Area between carpal cavity and two pre‐dactyl ridges rugose. On articulating cuff (Figure 17C.m), short row of 5–6 tubercles. Second pre‐dactyl tubercle row (Figure 17C.n) longer, curved. Long row of tubercles (Figure 17C.o) follows edge of pollex to distal tip. Segments of ambulatories (Figure 17E) with a few setae but no pubescence; merus width of third leg about 38% length. Type locality: Puerto Pizarro, Peru. Range: Costa Rica to northern Peru. Remarks: A molecular variant was revealed that we designate as *Leptuca sp*. Possibly, Crane (1975, 327, specimen # 3—NYZS 44200 and 44,201) mentioned this as an undetermined specimens from La Salada in Guayaquil, Guayas, and Puerto Bolivar, El Oro, Ecuador.

**FIGURE 18:**
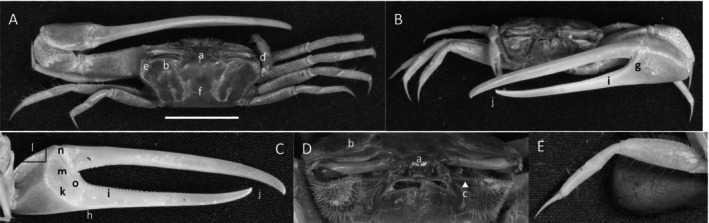
*Leptuca festae* (Nobili, 1901) (UNI 751) collected Isla Santay, Rio Guayas, GUA. (A) Dorsal view of male. Bar = 10 mm scale. (B) Front view. (C) Inner cheliped. (D) Ocular view. (E) Third ambulatory. a—frontal region, b—upper orbital margin, c—dentations of lower orbital margin, d—antero‐lateral angle, e—anterior lateral line, f—H‐depression, g—outer pre‐dactyl tubercle line, h—keel, i—pollex tubercles, j—pollex and dactyl terminus, k—oblique ridge, l—dorsal carina, m—pre‐dactyl tubercles, n—articulation cuff, and o—tubercle line of pollex.

***Leptuca festae*** (Nobili, 1901) (Figure 18A–E). Moderate‐sized. Carapace width up to 22 mm. Frontal region (Figure 18A.a,D.a) spatula‐form, 20% of carapace width. Upper orbital margins (Figure 18A.b,D.b) weakly angled. Eyebrows almost equal to eyestalk width. Suborbital ridge with distinct, small conical dentations (Figure 18D.c). Anterolateral angle (Figure 18A.d) forming a sharp right angle. Anterior portion of lateral line (Figure 18A.d) perpendicular to frontal margin. Distal portion tuberculate and curving toward the midline. Posterior striae prominent and raised. Carapace rectangular, length 60% width (Figure 18A), smooth, carapace semi‐circular in lateral view. H‐depression (Figure 18A.e) deep and accentuated with extensive pile in grooves. Dactyl and pollex (Figure 18B,C) very long compared to manus (ratio: dactyl + manus/manus = 3.3) in adult males. Both pollex and dactyl slim and slightly tubular. Outer manus (Figure 18B) with large tubercles becoming smaller toward the ventral surface. Outer articulation cuff colored yellow‐orange with two parallel rows of tubercles (Figure 18B.g). Pre‐dactyl line with only 3–4 tubercles (Figure 18B.g). Line of tubercles running from the articulation cuff along the gap to the base of the pollex (Figure 18B.i). Ventral keel weak (Figure 18B.h,C.h). Gap about twice the width of the dactyl. Outer surface of dactyl and pollex granular; large tubercles on the posterior portion of the pollex near the articulation cuff (Figure 18B.i,C.i). Distal terminus is a simple upward hook (Figure 18B.j,C.j). Proximal end of dactyl (Figure 18C.k) with 5–7 large tubercles in the gap; distal end overhangs the pollex slightly (Figure 18B.j,C.j). Inner surface of dactyl and pollex smooth, inner manus primarily smooth. Prominently raised oblique ridge (Figure 18C.m) continuous from the base of the pollex to the carpal cavity, then continues around edge and then away from the dorsal carina (Figure 18C.n) toward the dactyl. Area between the carpal cavity and pre‐dactyl ridge smooth. Articulation on inner manus with two rows of tubercles: (1) pre‐dactyl line of three tubercles (Figure 18C.o), (2) articulation cuff line of five tubercles (Figure 18C.p). A disconnected third row of tubercles (Figure 18C.o) from the lower articulation cuff onto the pollex. Ambulatories (Figure 18E) moderately setose with, little pubescence. Type locality: Rio Duale, Esmeraldas, Ecuador. Range: El Salvador to southern Ecuador.

**FIGURE 19:**
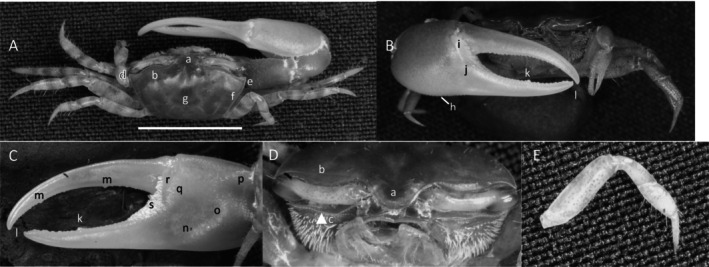
*Leptuca helleri* (Rathbun, 1902) (UNI 777) collected Playa los Alemanes, Isla Santa Cruz, GAL. (A) Dorsal view of male. Bar = 10 mm scale. (B) Front view. (C) Inner cheliped. (D) Ocular view. (E) Third ambulatory. a—frontal region, b—upper orbital margin, c—lower orbital margin dentations. d—antero‐lateral angle, e—anterior portion lateral line, f—posterior portion lateral line, g—H‐depression, h—tubercate keel, i—articulating cuff, j—tuberlce line, k—central tooh of pollex, l—dactyl pollex terminus, m—teeth of dactyl, n‐ oblique ridge, o—ridge apex, p—carina of upper carpal cavity, q—pre‐dactyl tubercle line, r—articulation cuff, and s—prominal dactyl pile.

***Leptuca helleri*** (Rathbun, 1902) (Figure 19A–E). Small species. Carapace width up to 12 mm. Frontal region (Figure 19A.a,D.a) about 30% of carapace width. Upper orbitals margin (Figure 19D.b) strongly angled with margins beaded and straight. Eyebrows about half eyestalk width. Suborbital margin with square dentations (Figure 19D.c). Anterolateral angle (Figure 19A.d) pointed outward. Anterior lateral margins (Figure 19D.e) straight. Posterior lateral margin (Figure 19A.f) almost straight but curving inward at terminus. Pair of single posterior striae. Carapace length about 63% width (Figure 19A), surface granular and shiny, no pubescence; semi‐circular in lateral view. H‐depression shallow (Figure 19A.g). Manus slightly shorter than dactyl and pollex (Figure 19B). Dorsal surface of outer manus with large tubercles becoming fine ventrally. Ventral keel short terminating at base of pollex (Figure 19B.h). Outer dactyl articulation cuff underdeveloped (Figure 19B.i). Dactyl and pollex thin and blade‐like, surface finely granular. Gap wide, approximately 1.5 X width of adjacent dactyl or pollex. Surface at base of pollex flat and smooth. Line of tubercles (Figure 19B.j) commence at lower edge of cuff and proceed on pollex to follow gap. Central large tooth (Figure 19B.k,C.k) slightly distal to mid‐length of pollex, distal end blunt (Figure 19B.l,C.l). Dactyl with two large tubercles (Figure 19C.m): one positioned approximately 1/3 distance from articulation, second pre‐terminal about 1/3 distance to end of dactyl. Distal dactyl curving downward (Figure 19B.l,C.l). Inner surface of dactyl, pollex, and manus finely granular with pits. Oblique ridge (Figure 19C.n) not reaching lower manus. High apex (Figure 19C.o) of tubercles at lower edge of carpal cavity. Tubercles follow edge of carpal cavity and blend with dorsal carina (Figure 19C.p). Area between carpal cavity and predactyl ridge finely granular with occasional tubercles. Predactyl line of tubercles (Figure 19C.q) starting at top of cuff and extending to base of dactyl. Articulation cuff (Figure 19C.r) without prominent tubercles. Dactyl articulation with large amount of pubescence (Figure 19C.s). Ambulatories (Figure 19E) with setae but lacking pubescence. Type locality: Fernandina (Narborough) Island, Galápagos Archipelago. Range: Endemic to the Galápagos Archipelago.

**FIGURE 20:**
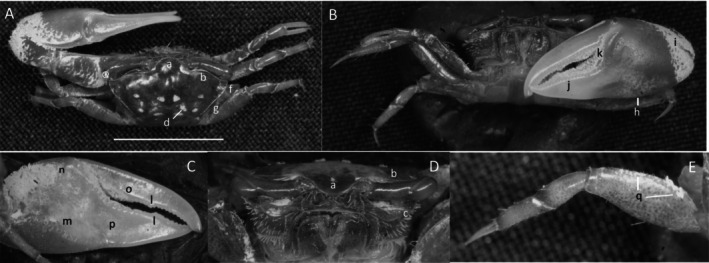
*Leptuca inaequalis* (Rathbun, 1935) (UNI 779) collected Malacón, San Lorenzo, ESM. (A) Dorsal view of male. Bar = 10 mm scale. (B) Front view. (C) Inner cheliped. (D) Ocular view. (E) Third ambulatory. a—frontal region, b—upper orbital margin, c—dentation of lower orbital margin, d—patches of pubescence, e—anterolateral angle, f—anterior lateral line, g—posterior lateral line, h—keel, i—pubescence on proximal manus, j—line of tubercles, k—pile at articulation, l—large tubercles, m—oblique ridge, n—dorsal carpal cavity carina, o—grove on inner dactyl, p—sulcus, and q—merus pubescence.

***Leptuca inaequalis*** (Rathbun, 1935) (Figure 20A–E). Small species. Carapace width up to 10 mm. Frontal region (Figure 20A.a,D.a) about 28% of carapace width. Upper orbital margin (Figure 20A.b,D.b) oblique to lateral margins. Eyebrow about 33% of eyestalk width. Lower orbital margin (Figure 20D.c) smooth except for one or more lateral teeth. Carapace trapezoid, slightly convex, length 70% of width (Figure 20A). Male carapace with eight small patches of pile (Figure 20A.d) forming two rows across the carapace. Deep central sulcus of H‐depression lies between the two rows of pile. Pile patchwork less distinct on the female. Antero‐lateral angle (Figure 20A.e) sharp. Anterior portion of lateral margin (Figure 20A.f) straight and pointing outward. Posterolateral line (Figure 20A.g) long and slightly angled toward the midline. Posterior stria very long and close to the ventral margin of the carapace. Pollex and dactyl length about equal to the manus (Figure 20B). Upper manus with large tubercles decreasing in size ventrally. Keel with tubercles (Figure 20B.h) on the lower margin of the manus and pollex. Proximal end of manus (Figure 20B.i) with thick pile/pubescence. Articulation cuff weak. Line of small tubercles (Figure 20B.j) follows the gap to near the end of the pollex. Outer pollex smooth, flat, and very broad with the distal tip strongly hooked upward. Gap small, ample pile on proximal dactyl and pollex (Figure 20B.k), central tubercle in the gap on both pollex and dactyl (Figure 20C.l). Pollex and dactyl curved inward. Surface of inner manus raised with an oblique ridge (Figure 20C.m) of separated large tubercles. Margin of carpal cavity smooth without tubercle carina. Distal dorsal carina (Figure 20C.n) angled toward the base of the dactyl. Dactyl articulation smooth without tubercles. Upper margin (Figure 20C.o) of dactyl with a groove and line of tubercles extending from the articulation toward the terminus but disappearing mid‐segment. Line of large, separated tubercles follows the gap to the central tooth on the pollex (Figure 20C.l). Pollex‐manus junction sulcate (Figure 20C.p). Ambulatories sparse in setae or pubescence except on the dorsal surface of the merus (Figure 20F.q). Type locality: Estero Salado, Guayaquil, Guayas, Ecuador. Range: El Salvador to northern Peru.

**FIGURE 21:**
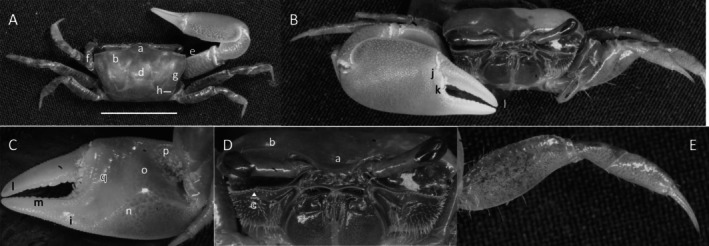
*Leptuca latimanus* (Rathbun, 1897) (UNI 784) collected Puerta las Javitas, SEL. (A) Dorsal view of male. Bar = 10 mm scale. (B) Front view. (C) Inner cheliped. (D) Ocular view. (E) Third ambulatory. a‐ frontal region, b—upper orbital margin, c—lower orbital dentations, d—H‐depression, e—antero‐lateral angle, f—anterior lateral line, g—posterior lateral line, h—posterior stria, i—submanus/pollex keel, j—articulation cuff, k—setae in articulation, l—terminus of dactyl and pollex, m—teeth of gap, n—oblique ridge, o—carpal cavity, p—setae, and q—line of tubercles.

***Leptuca latimanus*** (Rathbun, 1897) (Figure 21A–E). Moderate‐sized species. Carapace width up to 15 mm. Frontal region (Figure 21A.a,D.a) about 25% carapace width. Upper orbital margin (Figure 21A.b,D.b) not strongly angled. Eyestalk twice eyebrow width. Suborbital dentations (Figure 21D.c) strong. Carapace length about 62% width, surface finely granular, appearing smooth and dull, semi‐cylindrical in lateral view. H‐depression (Figure 21A.d) moderately deep, no pubescence. Anterolateral junction (Figure 21.e) forming a right angle. Anterior portion of lateral lines (Figure 21A.f) straight and parallel. Posterior lateral margins (Figure 21A.g) undulating in and out, terminal section curved slightly toward the mid‐line. Second stria more posterior (Figure 21A.h). Pollex and dactyl shorter than manus (Figure 21B); manus broad, covered with moderate to large tubercles extending onto the pollex and dactyl. Ventral keel with a fine tubercle line (Figure 21C.i). Articulation cuff indistinct (Figure 21B.j). Long, thick setae in articulations (Figure 21B.k). Gap narrow with edges of pollex and dactyl almost parallel, terminus of the two may not touch (Figure 21B.l,C.l), tip blunt. Tubercles in gap evenly sized (Figure 21C.m). Inner manus swollen with an obscure oblique ridge (Figure 21C.n), a few small tubercles forming a low, inconspicuous line. Inner palm glossy. Margins of lower carpal cavity (Figure 21C.o) smooth, no carina. Upper carpal cavity with evenly spaced large setae (Figure 21C.p). Lower inner manus with moderately large tubercles. Row of distinct tubercles (Figure 21C.q) following the articulation cuff and gap onto the pollex. Ambulatories (Figure 21E) moderately setose, little pubescence. Type location: La Paz, Baha California Sur, Mexico. Range: Northwestern Mexico to southern Ecuador.

**FIGURE 22:**
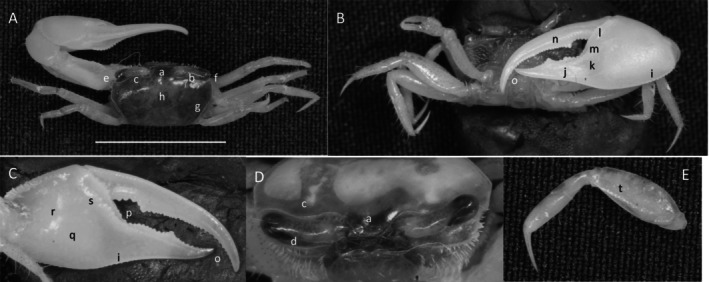
*Leptuca saltitanta* (Crane, 1941) (UNI 795) collected El Rompido, ESM. (A) Dorsal view of male. Bar = 10 mm scale. (B) Front view. (C) Inner cheliped. (D) Ocular view. (E) Third ambulatory. a—frontal region, b—preorbital sulcus, c—upper orbital margin, d—lower orbital dentations, e—antero‐lateral angle, f—anterior lateral line, g—posterior lateral line, h—H‐depression, i—keel, j—ventral pollex groove, k—manus‐pollex sulcus, l—articulation ridge, m—line of pollex tubercles, n—dactyl groove, o—dactyl and pollex terminus, p—V‐shaped tubercle formation, q—oblique ridge, r—carpal cavity, s—predactyl line of tubercles, and t—line of tubercles and pubescence on merus.

***Leptuca saltitanta*** (Crane, 1941) (Figure 22A–E). Very small species. Carapace width up to 10 mm. Front region (Figure 22A.a,D.a) moderately broad, 25% carapace width. Postorbital sulcus (Figure 22A.b) small and shallow. Upper orbital margin (Figure 22A.c,B.c) not angled, straight. Eyebrows short, less than one quarter eyestalk width. Lower orbital dentations (Figure 22D.d) undeveloped medially, becoming conical and progressively larger laterally. Anterolateral angle (Figure 22A.e) forming a right angle pointing forward. Anterior lateral margin (Figure 22A.f) straight and parallel. Posterior lateral margins (Figure 22A.g) strongly curved toward the midline. Posterior striae distal. Carapace length 64% width (Figure 22A), smooth and shiny, semi‐circular when viewed laterally. H‐depression and hepatic regions distinct (Figure 22A.h). Cheliped with an unusual teardrop shape (Figure 22B,C). Pollex and dactyl slightly longer than manus. Outer manus with dorsal tubercles fine, becoming courser ventrally. Keel (Figure 22B.i,C.i) starting as a line of tubercles on the ventral manus, extending as a sharp ridge to the pollex terminus. Ventral outer face of pollex with a line of fine tubercles (Figure 22B.j) extending from the base onto the lateral surface of the pollex. Dactyl and pollex initially flexed outward, curving inward distally (Figure 22A). Gap narrow. Outer pollex with a large triangular sulcus (Figure 22B.k) at the base narrowing toward terminus; base of pollex about 2–3 times the width of the dactyl. Dactyl articulation (Figure 22B.l) unusual, cuff horizontal rather than vertical and no tubercles. Ridge of tubercles lining the gap (Figure 22B.m) from articulation to near the terminus of the pollex. Line of fine tubercles on dactyl lining the gap (Figure 22B.n). Pollex thick at the articulation become thinner distally. Pollex with inner and outer rows of tubercles in the gap forming a v‐shaped depression. Pollex tip a simple hook (Figure 22B.o,C.o). Likewise, two rows of tubercles on the dactyl lining the gap. Inner row with two, evenly spaced, large tubercles (Figure 22C.p). Dactyl terminus long and strongly hooked over the pollex. Inner surface of manus raised. Oblique ridge (Figure 22C.q) weak, terminating in an apex at the edge of the carpal cavity with 8–10 large, distinct, conical tubercles. From the apex, a row of low tubercles progresses to the dorsal rim of the cavity (Figure 22C.r). Field of small tubercles covers the space between the cavity edge and the articulation cuff. Pre‐dactyl line of tubercles (Figure 22C.s) beginning with larger tubercles proximal to the cuff; line extending the entire length of the inner pollex to near the tip. Merus of ambulatories (Figure 22E.t) with ventral pubescence on tubercles. Type location: La Boca, Balboa Island, Panama. Previous range: El Salvador to Colombia, new to Ecuador.

**FIGURE 23:**
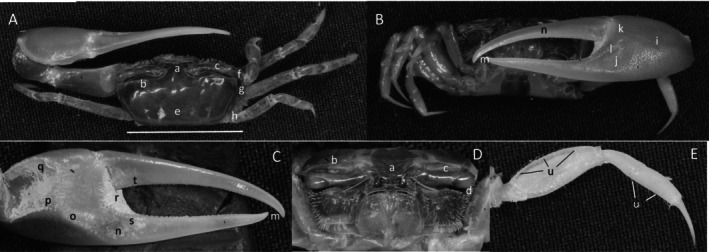
*Leptuca stenodactylus* (H. Milne Edwards and Lucas, 1843) (UNI 749) collected Estero Salado, Posorja, GUA. (A) Dorsal view of male. Bar = 10 mm scale. (B) Front view. (C) Inner cheliped. (D) Ocular view. (E) Third ambulatory. a—frontal region, b—preorbital sulcus, c—upper orbital margin, d—lower orbital dentations, e—H‐depression, f—anterior lateral angle, g—antero‐lateral junction, h—posterior stria, i—manus, j—pollex‐manus sulcus, k—articulation ridge, l—line of pollex tubercles, m—dactyl and pollex terminus, n—inner manus‐pollex sulcus, o—oblique ridge, p—apex and carina of carpal cavity, q—upper carina, r—setae at articulation junction, s—line of tubercles on pollex, and t—line of tubercles on dactyl.

***Leptuca stenodactylus*** (H. Milne Edwards and Lucas, 1843) (Figure 23A–E). Small species. Carapace width up to 13 mm. Frontal region (Figure 23A.a,D.a) 27% carapace width. Anterior margin smooth and curving (Figure 23A.b,D.b). Eyebrow about the same width as the adjacent eyestalk (Figure 23A.c,D.c). Lower orbital margin with small tubercles in the mid‐region, becoming larger laterally (Figure 23D.d). Carapace surface smooth and glossy, length 62% of width (Figure 23A). Center of H‐depression shallow and raised (Figure 23A.e). Sparse pubescence in posterior sutures. Lateral sutures deep, forming a W‐shape toward the posterior (Figure 23A.f). Antero‐lateral junction forming a right angle (Figure 23A.g). Junction between anterior and posterior lateral margin smooth (Figure 23A.h). Posterior lateral margin weak. Posterior stria short and weakly developed (Figure 23A.i). Dactyl and pollex more tubular, not blade‐like. Dactyl arched and slightly longer than pollex (Figure 23B,C). Upper manus with fine tubercles larger than those of the ventral surface. Manus‐pollex junction forming a large, smooth, triangular sulcus (Figure 23B.j). Ventral keel row of fine tubercles extending toward the pollex tip. Surface of pollex smooth. Dactyl articulation smooth and unornate (Figure 23B.k), row of tubercles starting below articulation follows gap to distal end of pollex (Figure 23B.l). Tubercles becoming minute on distal pollex, and end portion hooked upward (Figure 23B.m,C.m). Outer surface of dactyl smooth, tubercle teeth small in gap (Figure 23B,C). Inner manus surface smooth with sulcate manus‐pollex junction (Figure 23.n). Oblique ridge, single row of tubercles extending upward to carpal cavity (Figure 23C.o). Larger tubercle at mid‐length. Proximal end of ridge following anterior margin of cavity and integrating into a field of small tubercles (Figure 23C.p). Dorsal margin of carpal cavity lined with carina and long setae (Figure 23C.q). Dactyl articulating junction profuse with setae (Figure 23C.r). A row of tubercles follows edge onto base of pollex then dissipates (Figure 23C.s). Inner, dorsal pollex smooth, appearing tubular. Surface of inner dactyl smooth with small tubercles near articulation (Figure 23C.t). Ambulatory, very sparse but numerous setae (Figure 23E.u). Type location: “Orbigny,” Valparaiso, Chile. Range: Gulf de Fonseca, El Salvador to Algarroba, Chile.

**FIGURE 24:**
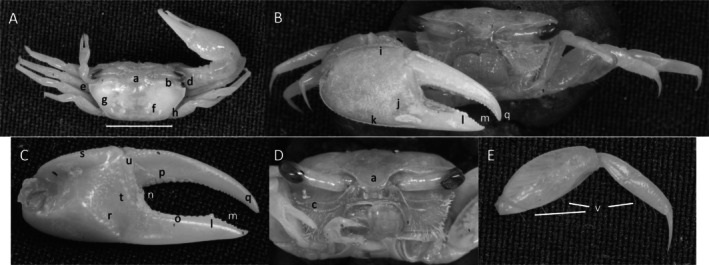
*Leptuca tallanica* (von Hagen, 1968) (USNM 138838) collected Rio Guayas, Puerto Bolivar, ELO. (A) Dorsal view of male. Bar = 10 mm scale. (B) Front view. (C) Inner cheliped. (D) Ocular view. (E) Third ambulatory. a—frontal region, b—anterior margin, c—lower orbital dentations, d—antero‐lateral angle, e—anterior lateral margin, f—pubescent commas, g—posterior lateral line, h—lateral stria, i—upper manus pubescence‐ filled groove, j—pubescence in triangle, k—keel, l—subterminal tooth, m—line of tubercles, n—pubescence at dactyl junction, o‐ tubercle row on proximal pollex, p—line of large tubercles on dactyl, q—dactyl terminus, r—oblique ridge, s—carina lining upper edge of carpal cavity, t—predactyl line of tubercles, u—articulation tubercles, and v—setae on walking legs.

***Leptuca tallanica*** (von Hagen, 1968) (Figure 24A–E) (USNM 138838). Small species. Carapace width up to 12 mm. Frontal region about 30% of carapace width (Figure 24A.a,D.a). Anterior margin finely beaded, moderately angled (Figure 24A.b). Eyebrow ⅓ to ½ eyestalk width. Lower orbital margin evenly dentate (Figure 24D.c). Anterolateral angle curved anteriorly (Figure 24A.d). Anterior lateral margin short (Figure 24A.e). Carapace length about 55% of width (Figure 24A), surface smooth. H‐depression shallow with comma‐shaped patches of pile on either end of the mesogastric‐cardiac line (Figure 24A.f), four small patches across branchial and cardiac regions of the male. Posterior lateral line strongly curved toward the mid‐line (Figure 24A.g). Single, beaded stria in posterior(Figure 24A.h). On the large cheliped, short dorsal manus groove filled with pubescence (Figure 24B.i). Moderate‐sized tubercles of the upper manus becoming small ventrally; lower outer manus with distinct, smooth triangular area with pubescence (Figure 24B.j) at base of pollex underlined with a row of tubercles. Propodus keel a short line of tubercles (Figure 24.k) extending to the base of pollex. Pollex and dactyl are equal or longer than manus (Figure 24B,C), blade‐like, and bases broad. Surface shiny but finely granular with numerous pits. Pollex with large subterminal tubercle (Figure 24B.l,C.l) followed by an obtuse‐angled terminal portion with a row of teeth (Figure 24B.m,C.m). Gap at base of pollex and dactyl profuse with pubescence (Figure 24C.n). Row of tubercles follows outer edge of pollex gap up to the large tooth (Figure 24C.o). Larger tubercles proximal on dactyl in the gap (Figure 24C.p). Dactyl terminus sharp and pointed downward (Figure 24B.q,C.q). Inner surface of dactyl, pollex, and posterior‐ventral manus smooth. Inner manus with oblique ridge (Figure 24C.r) composed of two distinct, unaligned tubercle rows terminating in the apex at the carpal cavity. Anterior edge of carpal cavity with a field of tubercles, no carina. Carina lining the upper edge of the cavity (Figure 24C.s). Strong pre‐dactyl line of tubercles (Figure 24C.t) extending to the base of pollex. Dactyl articulation with three large tubercles (Figure 24C.u), lower margin (Figure 24C.p) with two rows of large tubercles decreasing in size toward the terminus. Walking legs slender, setae and pubescence almost absent (Figure 24E.v). Type location: Puerto Pizarro, Peru. Range: Southern Ecuador to northern Peru.

**FIGURE 25:**
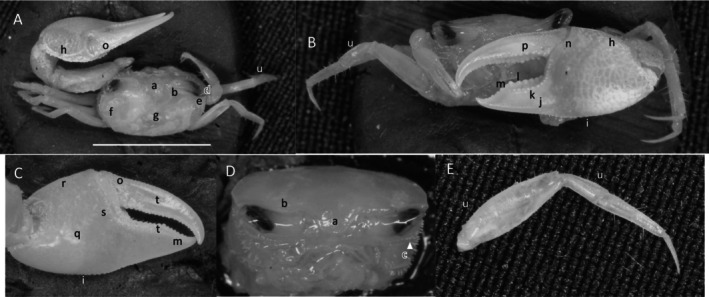
*Leptuca tenuipedis* (Crane, 1941) (USNM 79404) collected Costa Rica, Puntarenas, Nicoya Peninsula, Ballena Bay. (A) Dorsal view of male. Bar = 10 mm scale. (B) Front view. (C) Inner cheliped. (D) Ocular view. (E) Third ambulatory. a—frontal region, b—anterior margin, c—lower orbital dentations, d—antero‐lateral angle, e—anterior lateral margin, f—posterior lateral margin, g—H‐depression, h—tubercles on manus, i—keel, j—submarginal line, k—tubercle line, l—tubercle/tooth, m—obtuse edge, n—dactyl junction, o‐ tubercle line on dorsal dactyl, p—line of large tubercles on ventral dactyl, q—swollen inner manus, r—tubercles, s—row of tubercles on pollex, t—distal tubercle ridge, and u—pubescnce on merus and carpus of walking legs.

***Leptuca tenuipedis*** (Crane, 1941) (Figure 25A–E) (USNM 79404). A very small species. Carapace width up to 7.1 mm. Frontal region (Figure 25A.a,D.a) about 25% of carapace width. Anterior margin smooth, upper orbital margin smooth. Eyebrow about one‐fourth the width of eyestalk (Figure 25A.b,D.b). Lower orbital margin with small medial tubercles becoming large, rectangular dentations laterally (Figure 25D.c). Anterolateral angle about 90° (Figure 25A.d), pointing inward. Carapace width 69% of length (Figure 25A) surface rugose and slightly swollen (Figure 25A). Anterior lateral margin short (Figure 25A.e), posterior margin long, smooth and curving toward midline (Figure 25A.f). A single, short posterior stria is present on either lateral surface. H‐depression and lateral sutures (Figure 25A.g) not distinct and lack pubescence. Blade‐like dactyl and pollex slightly longer than manus (Figure 25B,C). Gap thin. Line of tubercles across dorsal manus (Figure 25A.h,B.h) diverging from carpal carina. Outer manus surface covered with small tubercles. Ventral keel (Figure 25B.i,C.i) of tubercles ending at base of pollex; submarginal line of fine tubules converging with keel. Pollex rugose with submarginal line protruding near pollex‐manus junction (Figure 25B.j). Dorsal edge of pollex with three tubercle ridges (Figure 25B); outer surface (Figure 25B.k) with a line of tubercles following gap, converging with a row of larger tubercles originating on inner pollex (Figure 25B.l). Third tubercle row on pollex center with mid‐length large tooth where pollex forms obtuse angle (Figure 25B.m). Pollex terminus sharp. Dactyl articulation smooth (Figure 25B.n). Dorsal surface of dactyl with cluster of fine tubercles (Figure 25C.o) near base extending toward dactyl tip. Exterior surface smooth; row of larger tubercles lining gape dissipating just beyond dactyl tooth (Figure 25B.p). Dactyl tip sharp and curving downward. Inner surface of pollex and dactyl smooth (Figure 25C). No oblique ridge is present, but center of palm swollen (Figure 25C.q). Carpal cavity shallow; no carina. Tubercles on dorsal interior manus (Figure 25C.r) ending sharply, making upper manus smooth. Row of large tubercles beginning at dactyl junction (Figure 25C.s) and following gape to terminus; ventral large teeth (Figure 25C.t) on dactyl extending to terminus. Walking legs (Figure 25E) very slender, tube‐like, and sparse in setae. A small patch of pubescence is present on dorsal merus (Figure 25A.u,B.u,E.u) near coxa junction and dorsal carpus. Type location: Ballena Bay, Nicoya Peninsula, Puntarenas Province, Costa Rica. Range: El Salvador to northern Peru.

**FIGURE 26:**
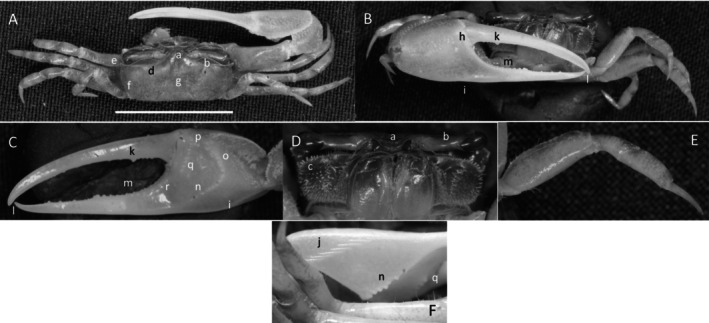
*Leptuca terpsichores* (Crane, 1941) (UNI 798) collected Malácon, San Lorenzo, ESM. (A) Dorsal view of male. Bar = 10 mm scale. (B) Front view. (C) Inner cheliped. (D) Ocular view. (E) Third ambulatory, (F) Ventral carpal stridulating ridges. a—frontal region, b—upper orbital margin, c—lower orbital dentations, d—pre‐orbital sulcus, e—antero‐lateral angle, f—posterior lateral line, g—H‐depression, h—articulation cuff, i—keel, j—stridulating ridges, k—dactyl tooth, l—terminus of dactyl and pollex, m—proximal row of pollex teeth, n—oblique ridge, o—upper carpal carina, p—tubercle line, q – predactyl cuff tubercles, and r – pollex tubercle row.

***Leptuca terpsichores*** (Crane, 1941) (Figure 26A–F). Small species. Carapace width up to 12 mm. Frontal region (Figure 26A.a,D.a) truncated, about 29% of carapace width. Angle of upper orbitals (Figure 26A.b,D.b) slightly oblique from the midline. Eyebrows are about the same width as the eyestalk. Lower orbital margin (Figure 26A.c) has distinct conical dentations, becoming square laterally. Curved sulcus posterior to the eyebrows (Figure 26A.d). Anterolateral angle (Figure 26A.e) sharp, pointing outward. Anterior lateral line (Figure 26A.e) short, posterior line long and gently curving (Figure 26A.f). Terminus is straight. No posterior stria. Carapace length is 61% of width (Figure 26A), smooth with no pile or pubescence, semi‐cylindrical from a lateral view. H‐depression (Figure 26A.g) distinct and the brachial region is swollen. Pollex and dactyl are much longer than the manus (Figure 26B,C). Outer manus surface has minute tubercles. Articulation cuff is poorly developed (Figure 26B.h). Keel (Figure 26B.i,C.i) sharp peak on the ventral manus and base of the pollex. Posterior ventral manus has 6–8 elongate, parallel stridulating ridges (Figure 26F.j) progressively staggered toward the base of the pollex. (Also, stridulating tubercles present on anterior surface of carpus and merus of first ambulatory leg behind cheliped). Pollex and dactyl are tubular, long, and covered with minute tubercles. Gap is three to four times wider than the dactyl. Dactyl has a single, large proximal tubercle on the first quarter (Figure 26B.k). Distal dactyl overhangs the tip of the pollex (Figure 26B.l,C.l). Pollex has a line of large tubercles to mid‐length (Figure 26B.m). A few larger tubercles are spaced on the distal pollex. Terminus is a simple, upturned hook (Figure 26B.l,C.l). Inner manus is smooth/shiny, with an oblique ridge (Figure 26C.n,F.n) extending from the base of the pollex to the edge of the carpal cavity then curving upward along its edge (Figure 26C.o). At the most dorsal point, a line of tubercles curves forward (Figure 26C.p), extending to the dactyl base. Articulation cuff is undeveloped. Predactyl cuff (Figure 26C.q,F.q) composed of 6–8 large tubercles. A second row of tubercles (Figure 26C.r) follows the edge of the pollex gap. Ambulatories (Figure 26E) sparse with setae and pubescence. Type location: La Boca, Balboa, Panama. Range: Southern Guatemala to northern Peru.

**FIGURE 27:**
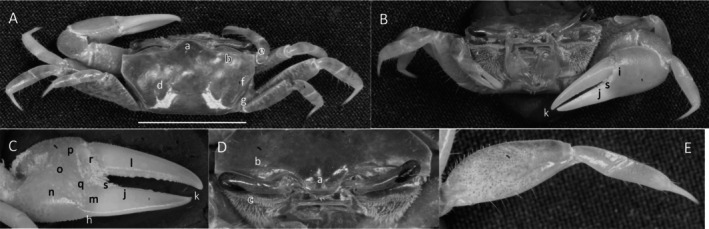
*Leptuca tomentosa* (Crane, 1941) (UNI 804) collected Simón Bolivar, SEL. (A) Dorsal view of male. Bar = 10 mm scale. (B) Front view. (C) Inner cheliped. (D) Ocular view. (E) Third ambulatory. a—frontal region, b—upper orbital margin, c—lower orbital dentations, d—pubescence on posterior carapace, e—anterolateral angle, f—posterior lateral line, g—postereior stria, h—keel, i—articulating cuff, j—row of pollex teeth, k ‐terminus of dactyl and pollex, l—dactyl teeth, m—sulcus, n—oblique ridge, o—carpal carina, p‐ tubercle patch, q—pre‐dactyl tubercles, r—articulation cuff, s—pile.

***Leptuca tomentosa*** (Crane, 1941) (Figure 27A–E). Moderate‐sized species. Carapace width up to 18 mm. Frontal region (Figure 27A.a,D.a) square‐shaped, 24% of carapace width. Upper orbital margin (Figure 27A.b,D.b) weakly angled to lateral margins. Eyebrow width equal to eyestalk diameter. Suborbital margin with large, square dentations (Figure 27D.c) decreasing in size laterally. Carapace length 62% width (Figure 27A), surface rugose with small pits (Figure 27A.d). Wooly pile forming J‐ or U‐shapes in groove between brachial, hepatic and mesogastic regions but absent from horizontal cardiac‐mesogastric groove. A larger area is covered in females than males. Anterolateral angle (Figure 27A.e) sharp with patches of pile and setae. Anterior end of lateral line beaded and short, margins parallel bilaterally. Posterior lateral margin (Figure 27A.f) long and distal end curving sharply toward mid‐line. Two pairs of posterior beaded stria (Figure 27A.g): First forming a sharp right‐angle just below the terminus of lateral line. Second, lower, straight‐line on lateral branchial wall. Dactyl and pollex longer than manus (Figure 27B,C). Upper half of outer manus with moderate‐sized tubercles, lower face with smaller tubercles. Ventral keel (Figure 27C.h) is weak, terminating at the base of pollex. Outer surface of dactyl and pollex smoothly rugose. A smooth, flat, circular area at pollex base, no pubescence or pile. Articulation cuff smooth with no line of tubercles. A line of tubercles on pollex follows the lower margin of gap (Figure 27B.i). Gap narrow to moderately wide, smaller than the width of pollex. Pollex tubercles generally small except a ridge of larger ones on proximal end (Figure 27B.j,C.j). Ridge ends with a larger fused tubercle. Terminus of pollex simple (Figure 27B.k,C.k). Dactyl with proximal row of large tubercles running about one fourth length (Figure 27B.l,C.l). Two distinct tubercles distal to mid‐length. Distal end of dactyl simple, hooked downward and hangs over pollex (Figure 27B.k,C.k). Inner surface of dactyl and pollex sulcate and cupped inward. Inner manus with center strongly elevated (Figure 27C.m) making inner surface of pollex sulcate. Elevated oblique ridge (Figure 27C.n) of single large tubercles extending from the base of pollex to carpal cavity. Line of tubercles from apex along anterior edge of carpal cavity (Figure 27C.o) weakly developed. Dorsal edge of carpal cavity with line of large tubercles (Figure 27C.p) projecting toward dactyl base. Tubercle field between carpal cavity and predactyl ridge low with scattered patches of pile. A line of large tubercles forming predactyl ridge (Figure 27C.q) extending to the base of pollex. Articulation cuff with row of 5 or 6 large tubercles. A line of pile between two ridges (Figure 27C.r). Articulation with large setae and pile extending distally from pollex‐dactyl junction (Figure 27B.s,C.s). In males, ambulatories with little pile or pubescence. Numerous large, long setae on merus, carpus, propodus, and dactyl (Figure 27E). A larger amount of wooly pubescence is on ambulatory legs of females. Type location: Gulf of Nicoya, Puntarenas, Costa Rica. Range: El Salvador to northern Peru. Remarks: Crane (1975) reclassified the *L. mertensi* of von Hagen (1968) from Peru as *L. tomentosa* .

**FIGURE 28:**
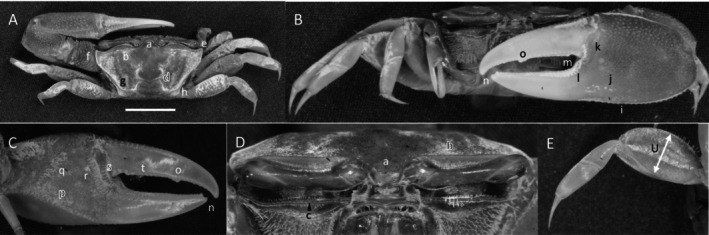
*Leptuca umbratila* (Crane, 1941) (UNI 805) collected El Rompido, ESM. (A) Dorsal view of male. Bar = 10 mm scale. (B) Front view. (C) Inner cheliped. (D) Ocular view. (E) Third ambulatory. a—frontal region, b—upper orbital margin, c—lower orbital dentations, d—H‐depression with pubescence, e—anterolateral angle, f—anterior lateral margin, g—posterior lateral margin, h—posterior stria, i—keel, j—pollex manus junction, k—articulation cuff, l—line of tubercles, m‐ pile in gap, n—pollex dactyl terminus, o—dactyl tooth, p ‐obliques ridge, q—carpal cavity carina, r‐ first row of tubercles, s—tubercles on articulation cuff, t—row of tubecles on dactyl, and u—width of ambulatory merus.

***Leptuca umbratila*** (Crane, 1941) (Figure 28A–E). Large species. Carapace width up to 30 mm. Frontal region (Figure 28A.a,D.a) narrow about 19% of carapace width. Upper orbital margin (Figure 28A.b,D.b) beaded, sinusoidal in shape, slightly angled toward lateral margin. Eyebrows visible from above (Figure 26A), about same width as eyestalks. Lower margin of eyebrow beaded. Lower orbital margin with large square dentations (Figure 28D.c). Carapace length 59% width (Figure 28A) surface rugose with moderate‐sized tubercles, abundance of pile or pubescence. In lateral view, carapace strongly arched not cylindrical. H‐depression (Figure 28A.d) shallow but filled with pile. Pile in lower sutures of H‐depression connected with lateral margins. Antero‐lateral angle sharp (Figure 28A.e). Anterior lateral margin (Figure 28A.f) curving outward slightly. Posterior portion of lateral margin (Figure 28A.g) strongly curving inward toward mid‐line giving carapace a shield shape. Poster stria (Figure 28A.h) beaded and very long. Pollex and dactyl slightly shorter than manus (Figure 28B,C). Dorsal half of outer manus with course tubercles becoming moderately sized on ventral face; ventral manus with keel (Figure 28C.i) of large tubercles terminating at base of pollex. Shallow sulcus at manus‐pollex junction (Figure 28B.j) may have patches of pubescence. Articulation cuff weak (Figure 28B.k), upper half smooth and 3–4 large tubercles on lower portions. Line of tubercles from cuff to pollex following gap (Figure 28B.l). Gape about half width of dactyl or pollex. Proximally, gap with ample pile (Figure 28B.m). Both dactyl and pollex smooth and moderately thick. Larger tubercles only at trifurcated tip of pollex (Figure 28B.n,C.n). Outer and inner edge of dactyl with lines of tubercles near gap. Proximal edge of dactyl with large tubercles lining gap. Single large tubercle (Figure 28C.o) about one third distance from dactyl terminus. Dactyl tip overhangs pollex terminus (Figure 28B.n,C.n). Inner surface of dactyl and pollex strongly sulcate. Surface of inner manus rugose to smooth in texture, raised with strongly tuberculated oblique ridge (Figure 28C.p) from base of pollex to carpal cavity. At cavity edge (Figure 28C.q), ridge makes a right angle extending toward upper margin of cavity. Dorsal cavity carina merges with ventral carina. Field of moderate‐sized, evenly space tubercles covering area proximal to articulation cuff. A second row of large tubercles (Figure 28C.r) on pre‐dactyl ridge extending from articulation on to pollex. A third row of large tubercles on dactyl articulation cuff (Figure 28C.s). Upper edge of dactyl (Figure 28C.t) with prominent row of large tubercles. Carpus behind cheliped with single, large central spine on mesial surface. Width of third ambulatory merus (Figure 28E.u) 60% length. Type location: Ballena Bay, Nicoya Peninsula, Costa Rica. Previous range: El Salvador to Colombia, new to Ecuador.

### 3.2 DNA Barcoding

A segment of COI, 658‐bp (base pairs) in length, from 73 Ecuador specimens or GenBank, along with sequences from other locations, resulted in 89 different haplotypes (Table 3). By including other related haplotypes, the COI segment was AT‐rich (63.5%) (T, 35.7%; A, 27.8%; G, 18.0%; C, 18.5%). Within this gene fragment, 250 positions were variable and 239 were parsimoniously informative.

**FIGURE 29:**
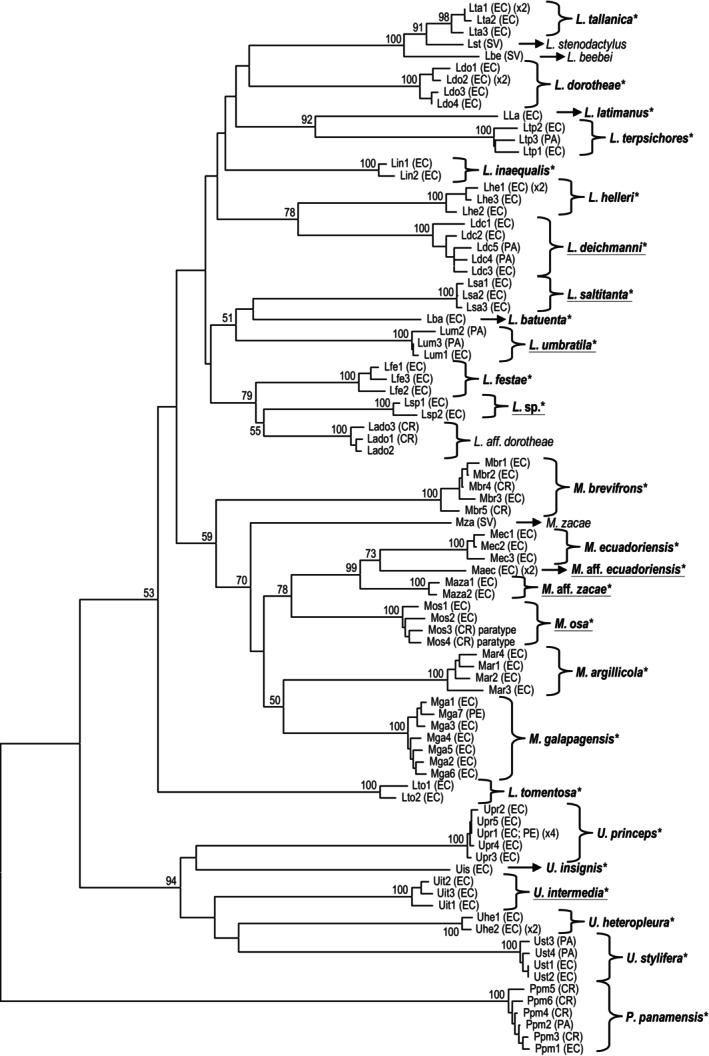
A neighbor‐joining COI tree for 30 operational taxonomic units (OTU) of fiddler crabs from Ecuador and other related taxa. Species names marked with “*” indicate specimens from Ecuador, and underlined species names are unidentified species or newly recorded species. EC—Ecuador, SV—El Salvador, CR—Costa Rica, PA—Panama, and PE—Peru. Only bootstrap values > 50% are shown on the nodes. For haplotype names, refer to Table 3.

The NJ tree, based on COI fragments (Figure 29), reveals that there are 27 clades from Ecuador, each corresponding to a specific species. The Ecuadorian species were identified through morphologic examination (see “Taxonomy”). All clades are highly supported. In the tree (Figure 29), three larger clades appear to be sister species: (1) *L. tallanica*, *L. stenodactylus*, and *L. beebei* ; (2) *L. latimanus* and *L. terpsichores*; and (3) *M. ecuadoriensis* , *M*. aff. *ecuadoriensis*, and *M*. aff. *zacae*.

The pairwise nucleotide divergences for COI with K2P distance are shown in Table S3. Most species are well separated from each other by a minimum divergence of 3.5% (between *L. tallanica* and *L. beebei* ), except for the smallest distance of 1.85% between *L. tallanica* and *L. stenodactylus*.

## 4 Discussion

The aim of this project is to identify species of fiddler crabs inhabiting the Tropical Eastern Pacific (TEP) shores and estuaries of Ecuador and the Galápagos Archipelago. As a corollary, we wish to supply information that will promote the conservation of coastal habitats and species diversity in the region. Primarily based on our fieldwork as well as records in the literature, we recovered 27 morphologically distinct species and identified three additional cryptic or pseudocryptic species using DNA molecular analysis. All taxa are associated with four genera, the largest number of species are in the *Leptuca*. On the other hand, only one species is associated with the *Petruca*. We also recovered six species in the *Uca* and five species in *Minuca*. For a global comparison to the total described species in each genus, Ecuador is home to 15 of the 30 *Leptuca* species, six of the nine *Uca* species, five of the 18 *Minuca*, as well as the sole species in the genus *Petruca*. In addition, we found two cryptic or pseudocryptic species related to the *Minuca* and one related to the *Leptuca*. Altogether, the coasts of Ecuador and the Galápagos Archipelago appear to support 30 possible species of fiddler crabs.

In recent years, several geographic studies have described the distribution of fiddler crabs around the Indo‐West Pacific Oceans (Silva, Mesquita, and Paula 2010; Aoki and Wada 2013; Shih, Ng, and Christy 2015; Fratini et al. 2016; Nehemia and Kochzius 2017; Tokuyama et al. 2020; Hardianto et al. 2022; Shih, Prema et al. 2022; Shih, Wong et al. 2022) and around the Atlantic Ocean (Barnwell and Thurman 1984; Thurman 2002, 2003a, 2003b; Thurman, Hanna, and Bennett 2010; Laurenzano, Farías, and Schubart 2012; Laurenzano, Mantelatto, and Schubart 2013; Laurenzano, Costa, and Schubart 2016; Wieman et al. 2014; Thurman, Faria, and McNamara 2013; Staton et al. 2014; Thurman et al. 2018, 2021; Marochi et al. 2022). Few studies have described the geographic distribution of fiddler crabs along the shores of the TEP province, which extends from El Salvador to northern Peru in Central and South America (Rosenberg 2020).

Since Crane published her tome in 1975, several surveys of fiddler crab species have appeared that include the Pacific coast of Mexico, as well as Central and South America. Hendrickx (1979, 1995) augmented the list of fiddler crabs from Mexico and reported 38 species for the eastern Pacific Ocean between Baja California, Mexico, and Chile. In 1985, Barnwell and Szelistowski found 21 fiddler crab species along the Rio Lagartos near Punta Morales on the Gulf of Nicoya, Costa Rica. Around the same time, Lemaitre and Alvarez León (1992) reported 26 species from the Pacific coast of Colombia. Twenty‐four species are thought to have equivalent ranges from El Salvador to Panamá (Rosenberg 2020). Many of those species have ranges extending south to the Gulf of Guayaquil or northern Perú. Furthermore, the TEP province can be subdivided into three distinct areas or subprovinces: (1) a northern area from El Salvador to Nicaragua, (2) a central area from Costa Rica to Colombia, and (3) a southern portion from Colombia to San Pedro/Sechura in northern Perú. The central region, Costa Rica to Colombia, has 30 to 31 known fiddler crab species. The northern and southern subprovinces are thought to contain about 24 species each, with different configurations of missing taxa. Missing from the north are *L. dorotheae*, *L. pygmaea* , *L. tallanica*, *M. argillicola* , *M. galapagensis* , *M. osa* , and *U. intermedia* . Absent in the south are *L. limicola* , *L. oerstedi* , *M. herradurensis*, and *M. zacae* . In terms of physiography, the TEP province lacks the chains of large offshore islands like those in the Indian Ocean, western Atlantic Ocean, as well as the central and western parts of the Pacific Ocean (Cortés 1997; Fiedler and Talley 2006; Neall and Trewick 2008). The Galápagos Archipelago (GAL) Province is a notable exception (Rosenberg 2020). The GAL Province is home to two fiddler crab species: *L. helleri* and *M. galapagensis* . Previously, Capparelli et al. (2021) characterized the local ecology and physiology of the two species on Isla Santa Cruz, GAL.

### 4.1 New Geographic Records

Based on morphology, we collected five species previously unreported in Ecuador (Table S2). In addition, molecular analysis revealed three cryptic or pseudocryptic species (Figure 29). The five taxa new to Ecuador are *U. intermedia* , *M. osa* , *L. deichmanni, L. saltitanta*, and *L. umbratila*. Three cryptic or pseudocryptic species identified using molecular techniques are also new for Ecuador: *L*. sp., *M*. aff. *ecuadoriensis*, and *M*. aff. *zacae*. We recovered *U. intermedia* from two locations: one in ESM and the other in MAN. *Minuca argillicola* was found at 10 locations in ESM while *M. brevifrons* at seven sites in ESM, SEL, and GUA. One specimen of *M. brevifrons* from Salado, GUA was discovered at the Smithsonian (USNM 70867). During the writing of this study, Ramos‐Veliz, Vergara, and Jorge (2022) corroborated the presence of *M. brevifrons* with specimens from Eloy Alfaro, MAN. With our study, it is clear the species is widely distributed in Ecuador. *Minuca osa* is known from Costa Rica and western Panama (Landstorfer and Schubart, 2010; Lombardo‐González 2024). Here, we report it from five locations in ESM and SEL. For the three species of *Leptuca*, *L. deichmanni* was taken at six locations in ESM and SEL while *L. umbratila* was collected at three locations and *L. saltitanta* from two sites in ESM. Almost all the new distributional records were from the northern coast of Ecuador, north of Puntilla de Salinas, SEL (−2.1838° S; −80.9940° W). Based on species distributions, it appears that the area between Cabo San Lorenzo (MAN) and P. d. Salinas (SEL) serves as a weak barrier separating some “northern” from “southern” taxa.

The three cryptic or pseudocryptic species will require further investigation before species descriptions appear. Crane and von Hagen both mentioned what appears to be the putative species *L*. sp. (Crane 1975, 327) and *M*. aff. *ecuadoriensis* (i.e., *Uca lanigera* von Hagen, 1968). To aid in the identification of the fiddler crabs from Ecuador, we have included a morphologic key. It is designed to be used in conjunction with the photographs and species definitions. Since the cryptic or pseudocryptic species were not morphologically distinct, they are not included in the morphology key.

### 4.2 Molecular Analysis

The sequence of the COI is suggested as the barcoding marker (Hebert et al. 2003; Hebert, Ratnasingham, and De Waard 2003), serving as an important reference for molecular thresholds among related species in certain crustacean groups (Lefébure et al. 2006; Costa et al. 2007; Chu et al. 2015). Several studies have successfully utilized DNA barcoding to distinguish closely related species of fiddler crabs (e.g., Shih, Prema et al. 2022 for taxa around the Arabian Sea; Shih, Wong et al. (2022) for Vietnamese species). In the molecular analysis based on COI, 30 operational taxonomic units (OTUs) were identified (Figure 29), with 27 originating from specimens and sequences in Ecuador; yet there is still a lack of DNA sampling for *U. ornata* , *L. beebei* , and *L. stenodactylus*, previously recorded in Ecuador (Crane 1975). Among the 27 OTUs, five new records for Ecuador, including *L. deichmanni*, *L. saltitanta*, *L. umbratila*, *M. osa,* and *U. intermedia* , as well as three unidentified species, including *L*. sp., *M*. aff. *ecuadoriensis*, and. *M*. aff. *zacae*. In time, these unidentified species may be further demonstrated as cryptic or pseudocryptic and even new species, for example, *Austruca occidentalis* (Naderloo, Schubart, and Shih 2016), *Tubuca alcocki* (Shih, Chan, and Ng 2018; Shih, Chan, and Ng 2018), *M. virens* (Salmon and Atsaides 1968; Thurman et al. 2018), and *M. panema* (Coelho 1972) (Thurman et al. 2021; Thurman, Shih, and McNamara 2023).

The NJ tree (Figure 29) indicates three larger clades composed of closely related species. One large clade includes *L. beebei* , *L. stenodactylus*, and *L. tallanica*, with at least the first two being very similar in morphology (Crane 1975, fig. 101). Another large clade consists of *L. latimanus* and *L. terpsichores*, which also share similar characters in a key (Crane 1975, 626). The last large clade is composed of *M. ecuadoriensis* , *M*. aff. *ecuadoriensis*, and *M*. aff. *zacae*. Two clades with large distances (4.26% in Table S1, Figure 29) are identified as *M. ecuadoriensis* . The type locality of *M. ecuadoriensis* (Maccagno, 1928) is “Esmeraldas, Ecuador” and the clade of “Mec1, Mec2 and Mec3” includes specimens from Esmeraldas and are, hence, considered *M. ecuadoriensis* . Another clade with specimens from Guayas, Ecuador, is tentatively treated as “*M*. aff. *ecuadoriensis*.” However, additional specimens of *U. lanigera* von Hagen, 1968 from this area need to be examined to resolve this conundrum. Similarly, the type locality of *M. zacae* (Crane, 1941) is Costa Rica, which is geographically closer to El Salvador (FN430710); thus, the genetically distinct specimen from Ecuador is tentatively treated as *M*. aff. *zacae*. Further evidence is necessary to confirm the identities of these species.

Based on the COI barcoding distances (Table S1), the smallest distance between *L. tallanica* and *L. stenodactylus* is 1.54%, whereas most interspecific distances exceed 3.6% (between *L. stenodactylus* and *L. beebei* ). The distance of 3.6% approximates the minimum distances of most fiddler crabs, for example, 3.84% between *Gelasimus hesperiae* and “Clade U” (Shih, Naruse, and Ng 2010); 3.78% between *Tubuca urvillei* and *T. alcocki* (Shih, Chan, and Ng 2018); and 3.78% between *Austruca perplexa* and *A. citrus* (Shih and Poupin 2020). Nonetheless, studies on coastal land crabs, including the Sesarmidae (e.g., 1.49% between *Leptarma liho* and *L. paucitorum*, Koller et al. 2010; 1.6% between *Neosarmatium africanum* and *N. meinerti*, Chu et al. 2015; and 0.92%–1.7% among *Parasesarma bidens*, *P. chiahsiang*, *P. continental*, *P. insulare*, and *P. sanguimanus*, Shih et al. 2023), and the Varunidae (e.g., 1.54% between *Pseudohelice subquadrata* and *P. annamalai*, Prema et al. 2022), have shown some interspecific distances as low as 1.54%, similar to those observed in this study. Beyond the COI distances, the significance of molecular evidence in supporting clade distinctions within tree topology is also essential to consider (Luo et al. 2018).

In the NJ tree (Figure 29), the support value for the genus *Leptuca* is quite low (below 50%) and *L. tomentosa* is positioned in its own clade, indicating that the COI marker is best suited for species‐level identification rather than higher taxonomic organization. After incorporation of more conservative mitochondrial (16S) and nuclear (28S) markers (Shih, Ng, and Christy 2015), the *Leptuca* clade, including *L. tomentosa*, receives strong support (unpublished data). COI barcoding alone may face challenges for species delimitation and identification due to issues like asymmetrical introgression and incomplete lineage sorting. Thus, it is recommended to integrate additional evidence from nuclear markers to enhance the taxonomy based on morphology (Chu et al. 2015; Eberle et al. 2019, 2020; Ahrens et al. 2021). Including nuclear 28S in studies of fiddler crabs, for instance, has proven valuable for clarifying the identities of *Xeruca formosensis* (Shih 2015), *Petruca panamensis* (Shih, Ng and Christy 2015), *Austruca variegata* (Shih et al. 2019), and for elucidating the phylogeny of *Minuca* (Thurman, Shih, and McNamara 2023).

In summary, using morphology and molecular data, we identified 30 species from the Pacific coast of Ecuador and the Galápagos Archipelago. Our investigation provides an accurate, contemporary checklist of species, employing DNA barcoding to verify collecting records. To recognize species morphologically, we have developed an identification key. In addition, we have included COI haplotype GenBank access numbers (Table 3). This study also establishes a convenient backdrop for ecological, morphologic, physiologic, and genetic investigations, as well as providing a platform for conservation efforts in Ecuador and the Galápagos Archipelago.

### 4.3 Conservation Implications

Fiddler crabs are dominant species in the ecologically complex mangroves, sandy beaches, and estuaries of tropical Ecuador. Through ecosystem “engineering,” soils are aerated and otherwise‐inaccessible food becomes available for other marine organisms (Kristensen 2008). By long‐distance dispersal of larvae, genetic diversity can be maintained effectively among remote populations (Grantham, Eckert, and Shanks 2003). Despite their ecological and recreational importance, coastal habitats are threatened constantly by human activities (Morocho et al. 2022). Clearing the mangroves for salt water and mariculture (Twilley et al. 1999; Mereci‐Guamán et al. 2021) and selective logging of mangrove trees (Jarmillo et al. 2023) continuously jeopardize the integrity of this native ecosystem. This devastation reduces significantly genetic diversity in fiddler crabs as well as in other species populations (Nehemis and Kochzius 2017). In the face of rapid habitat modification and a dearth of comprehensive scientific surveys, it has been difficult to take an accurate census of coastal species. Likewise, measurements of genetic diversity have been limited (Cruz et al. 2003). Our work reports an extremely diverse assemblage of 30 fiddler crab taxa along the coast of Ecuador and the Galápagos Archipelago. Since high genetic diversity contributes to population stability, restoring the cleared habitats is believed to be the most effective measure for the conservation of species and maintenance of high genetic diversity (Rodríguez 2018). The coast of Ecuador is at the convergence of ocean currents from both the north and south (Fielder and Talley 2006; Neall and Trewick 2008), thus larval dispersal from adjacent regions can ensure continued species and gene diversity. Conservation activity along the coast of Ecuador holds the hope for sustaining the health of marine species as well as communities (Jaramillo et al. 2016).

## Author Contributions

**Carl L. Thurman:** data curation (equal), investigation (equal), methodology (equal), writing – original draft (equal), writing – review and editing (equal). **John C. McNamara:** conceptualization (equal), data curation (equal), writing – review and editing (equal). **Hsi‐Te Shih:** data curation (equal), formal analysis (equal), funding acquisition (equal), writing – review and editing (equal). **Mariana V. Capparelli:** conceptualization (equal), data curation (equal), funding acquisition (equal), project administration (equal), resources (equal), writing – review and editing (equal).

## Ethics Statement

Fiddler crabs received no unethical treatment. They were collected by hand from their habitat and transported in habitat water to a holding laboratory. Before being killed by low temperature exposure, leg tissue samples were collected by autonomy and preserved in 95% ethanol. After a few hours in refrigeration (5°C), whole crabs were preserved in 80% ethanol. During collection and holding, live crabs were treated with care.

## Conflicts of Interest

The authors declare no conflicts of interest.

## Identification Key for 27 Species of Male Fiddler Crabs From Ecuador

Character:

1. A. Frontal region > 15% of total carapace width. Go to 7. (Genera *Petruca*, *Leptuca*, or *Minuca*)
  B. Frontal region < 15% of total carapace width. Go to 2. (Genus *Uca*)
2. A. Dactyl/pollex > 60% of propodus length. Go to 3.
  B. Dactyl/pollex < 60% of propodus length. Go to 4.
3. A. Large‐spiked tubercle on ventral edge of walking leg merus (Figure 5E.u). Carapace dorso‐lateral margin with 4–6 large tubercle spikes (Figure 5A.f). Keel on ventral edge of major cheliped with a broken line of tubercle spikes (Figure 5B.i)—*Uca ornata* (Smith, 1870).
  B. Small‐spiked tubercles on ventral edge of walking leg merus (Figure 3E.x). Carapace dorso‐lateral margin with 3–4 large tubercle spikes (Figure 3A.f). Tubercle keel on ventral major cheliped continuous from manus to pollex. (Figure 3B.k)—*Uca insignis* (H. Milne Edwards, 1852).
4. A. Gape between dactyl and pollex of major cheliped with pre‐terminal tooth at the distal end of pollex (Figures 2B.h and 6B.l). Go to 5.
  B. Gape between dactyl and pollex is small. Largest space is near dactyl articulation. (Figures 4B and 7b). Go to 6.
5. A. Distal large tooth on pollex of major cheliped about one third length from terminus (Figure 6B.l)—*Uca princeps* (Smith, 1870).
  B. Distal large tooth on pollex of major cheliped about one fifth length from terminus (Figure 2B.h)—*Uca heteropleura* (Smith, 1870).
6. A. In adults, the eyestalk on the side with the major cheliped has a long filamentous stylet (Figure 7B.d). The submarginal groove on the major cheliped is sparse in pubescence (Figure 7B.l)—*Uca stilifera* (H. Milne Edwards, 1852).
  B. Adult males lack a long stylet on the eyestalk (Figure 4A). The submarginal groove on the pollex of the major cheliped has abundant pubescence; the distal patch near the terminus oval‐shaped (Figure 4B.h)—*Uca intermedia* von Prahl and Toro, 1985.
7. A. Frontal region larger than 31%. Go to 8.
  B. Frontal region between 19% and 30% carapace width. Go to 9.
8. A. Frontal 33%. Carapace dorsal surface over lateral branchial regions profuse with pubescence (Figure 11A). Dactyl on major cheliped without large tubercle teeth in gap (Figure 11B,C)–*Minuca ecuadoriensis* (Maccagno, 1928).
  B. Frontal 33%. Carapace dorsal surface smooth with a small amount of pubescence in pre‐orbital groove. Dactyl of major cheliped with very large tubercle about one third length from articulation (Figure 10C.h). Dactyl and pollex nearly tubular with a large gape. Pollex nearly straight with numerous large tubercles (Figure 10C.i)—*Minuca brevifrons* (Stimpson, 1860).
  C. Frontal 35%. Dry carapace surface looks “polka‐dotted,” with microscopic pile‐filled pores. On major cheliped, blade‐like dactyl/pollex approximately equal to manus length (Figure 13B). Base of dactyl at articulation equal to or larger than gape (Figure 13C). Crest of oblique ridge on inner manus with a cluster of 5–6 tubercles at carpal cavity (Figure 13C.n)—*Minuca osa* (Landstorfer and Schubart, 2010).
9. A. Frontal between 19% and 30%; dried carapace with a large amount of pubescence or pile. Go to 10.
  B. Frontal between 19% and 30%; dried carapace with small portions or patches of pubescence. Go to 11.
  C. Frontal between 20% and 30%. Carapace without pubescence when dried. Go to 12.
10. A. Frontal width 19%. Lateral and posterior carapace thick, with pile. Dactyl and pollex of major cheliped about the same length as manus (Figure 28B,C). Both are flat, blade‐like. Carapace surface profuse with pubescence (Figure 28A)—*Leptuca umbratila* (Crane, 1941).
  B. Frontal width 23%. Pubescence evenly distributed over carapace, but most concentrated on lateral edges of anterior hepatic region (Figure 9A). Antero‐lateral angle pointed outward. A large tubercle on proximal dactyl near articulation, gape edge of dactyl with large tubercles (Figure 9B.i,C.i)—*Minuca argillicola* (Crane, 1941).
  C. Frontal width 24%. Pubescence concentrated in posterior of H‐depression and posterior edge of branchial region (Figure 27A.d). Gape between dactyl and pollex narrow. Low, evenly spaced tubercles are in the gape on dactyl (Figure 27C)—*Leptuca tomentosa* (Crane, 1941).
11. A. Frontal 26%. Pubescence pattern J‐shaped, limited to the posterior branchial region behind the H‐depression (Figure 14A.f). Dactyl and pollex blade‐like. Terminus of dactyl broad, with numerous small tubercles (Figure 14B.j,C.j)—*Leptuca batuenta* (Crane, 1941).
  B. Frontal 28%. Eight small patches or tufts of pubescence on the dorsal carapace, arranged in a rectangle around the H‐depression (Figure 20A.d)—*Leptuca inaequalis* (Rathbun, 1935).
  C. Frontal 30%. Pubescence in the distal ends of the H‐depression, forming 2–4 small, coma‐shaped patches (Figure 24A.f). Triangular area at the base of the pollex filled with pubescence. Large distal tubercle about two third the length of the pollex (Figure 24C.l)—*Leptuca tallanic*a (von Hagen, 1968).
12. A. Dactyl and pollex of large claw much shorter than manus. Go to 13.
  B. Dactyl and pollex of large claw much longer than manus. Go to 14.
13. A. Dactyl and pollex of large claw much shorter than manus (Figure 21B, C)—*Leptuca latimanus* (Rathbun, 1983).
  B. Dactyl and pollex about equal in length to manus. Inner edge of dactyl with large tooth and cleft near articulation (Figure 22B.n). Pollex with two mid‐length, large tubercles, end of pollex strongly scalloped to pointed terminus (Figure 22C.o)—*Leptuca saltitanta* (Crane, 1941).
  C. Dactyl and pollex about equal in length to manus. No large tooth or gap near articulation (Figure 25B.p). Pollex with one mid‐length large tubercle (Figure 25B.m). Terminus of pollex scalloped with distinct moderate‐sized teeth—*Leptuca tenuipedis* (Crane, 1941).
14. A. Dactyl and pollex of large cheliped blade‐like. Go to 15.
  B. Dactyl and pollex of large cheliped tubular. Go to 16.
15. A. Outer manus smooth, without tubercles. Inner manus smooth (Figure 8C). Terminus of dactyl and propodus form simple hooks (Figure 8B.g,C)—*Petruca panamensis* (Stimpson, 1859).
  B. Dactyl arched over pollex (Figure 16C). No pubescence at the dactyl‐manus junction (Figure 16C)—*Leptuca deichmanni* (Rathbun, 1935).
  C. Tip of pollex broad, with about 6–7 tubercles (Figure 12B.k,C.k)—*Minuca galapagensis* (Rathbun, 1902).
  D. Tip of pollex curved upward, small tubercles on terminus (Figure 19B)—*Leptuca helleri* (Rathbun, 1902).
16. A. Dactyl/pollex very long. Gape wide, with dactyl strongly arched. Go to 17.
  B. Frontal region 29% width. Dactyl/pollex long. Gape smaller than the width of the dactyl or pollex. Gap small, pollex with a large central tooth (Figure 15B.j). Pubescence at dactyl articulation (Figure 15B.g)—*Leptuca beebei* (Crane, 1941).
17. A. Carapace, frontal region 20% width. Dactyl/pollex very long. Gap between pollex and dactyl very wide. Dactyl plus manus 3.3 times longer than manus alone. Dactyl and pollex almost tubular in shape (Figure 18C). Carapace smooth and shiny (Figure 18A)—*Leptuca festae* (Nobili, 1901).
  B. Carapace frontal region 23% width. Dactyl strongly arched over pollex (Figure 17B). Pollex essentially straight, with pubescence at dactyl‐manus junction (Figure 17B.g)—*Leptuca dorotheae* (von Hagen, 1968).
  C. Carapace frontal region 27% width. Dactyl strongly arched over pollex (Figure 23B). Teeth on inner edges of dactyl and propodus very small. Triangular sulcus at pollex‐manus junction (Figure 23B.j)—*Leptuca stenodactylus* (H. Milne Edwards and Lucas, 1843).
  D. Carapace frontal region 29% width. Dactyl strongly arched over pollex (Figure 26B). Teeth on inner edges of dactyl and propodus large. Posterior ventral surface of manus has 6–7 stridulating ridges (Figure 26F.j)—*Leptuca terpsichores* (Crane, 1941).

## Supporting information


**Table S1.** Locations of fiddler crab collecting sites in Ecuador and the Galapagos Archipelago. ELO, El Oro; ESM, Esmeraldas; GAL, Galapagos; GUA, Guayas; MAN, Manabí; SEL, Santa Elena.
**Table S2.** Specimens examined. AMNH—F.H. Barnwell collection, American Museum of Natural History, NYC; RMNH—Naturalis Biodiversity Center, Leiden, the Netherlands; UNI—University of Northern Iowa collection, Cedar Falls, IA; USNM, U.S. Museum of Natural History (Smithsonian), Washington, DC; ZMA, Zoological Museum of Amsterdam (now in RMNH).

**Table S3.** Matrix of percentage of pairwise nucleotide divergences with Kimura 2‐parameter (K2P) distances based on the cytochrome c oxidase subunit I (COI) gene within and between 30 species of fiddler crabs from Ecuador and adjacent areas. Range of values are given in parentheses.

## Data Availability

The data that support the findings of this study are openly available in the Supporting Information.
