# Supplementary material for: Fiddler Crabs (Crustacea: Decapoda: Ocypodidae) From Coastal Ecuador and the Galápagos Islands: Species Descriptions and DNA Barcodes
Source: Ecol Evol. 2025 Jan 27;15(1):e70646. doi: 10.1002/ece3.70646 (PMC11770328; doi:10.1002/ece3.70646)
Supplement: Supplementary file 1 — Table S1. Locations of fiddler crab collecting sites in Ecuador and the Galapagos Archipelago. ELO, El Oro; ESM, Esmeraldas; GAL, Galapagos; GUA, Guayas; MAN, Manabí; SEL, Santa Elena. Table S2. Specimens examined. AMNH—F.H. Barnwell collection, American Museum of Natural History, NYC; RMNH—Naturalis Biodiversity Center, Leiden, the Netherlands; UNI—University of Northern Iowa collection, Cedar Falls, IA; USNM, U.S. Museum of Natural History (Smithsonian), Washington, DC; ZMA, Zoological Museum of Amsterdam (now in RMNH). [file ECE3-15-e70646-s001.pdf]

Table S1. Locations of fiddler crab collecting sites in Ecuador and the Galapagos Archipelago. Province abbreviations: ELO - El Oro, ESM – Esmeraldas, GAL- Galapagos, GUA - Guayas, MAN - Manabí, SEL - Santa Elena.

Name	Province	Latitude	Longitude
Mainland			
San Lorenzo Malecón	ESM	1.293132	-78.836180
Majagual (La Tola)	ESM	1.172266	-79.078686
El Rompido	ESM	1.115851	-79.134752
Puento Montalvo	ESM	1.050369	-79.315240
Rio Verde (Cevicangre)	ESM	1.049837	-79.416731
Playa Camarones	ESM	0.995565	-79.561478
Playa Achilube	ESM	0.985155	-79.610010
Santa Marta	ESM	0.975745	-79.650610
La Propicia	ESM	0.921122	-79.658765
Atacanes Lagoon	ESM	0.870170	-79.581677
Muisne	ESM	0.633350	-80.035866
Portete	ESM	0.485414	-80.043750
Agua Clara, Daul	ESM	0.418619	-79.999531
S.J. de Chamanga (beach)	ESM	0.272491	-79.963023
S.J. de Chamanga(mangel)	ESM	0.270103	-79.957946
So. Pedernales	MAN	-0.144840	-80.239100
Canoa	MAN	-0.460830	-80.457529
Bahia de Caraquez	MAN	-0.609714	-80.423802
Bahia de Caraquez	MAN	-0.642017	-80.369500
San Clemente	MAN	-0.767950	-80.513091
Manta	MAN	-0.948694	-80.697090
Manta	MAN	-0.950310	-80.716624
Punta Cayo	MAN	-1.353302	-80.739223
Puerto Lopez	MAN	-1.554870	-80.811660
Manglaraltos	SEL	-1.845219	-80.748104
Rio Chico	SEL	-1.862195	-80.742649
San Antonio, Rio Cadente	SEL	-1.869338	-80.737026
Puente Atravezando	SEL	-1.878911	-80.728566
Simon Bolivar	SEL	-1.879418	-80.735480
Puente Valdiva	SEL	-1.937757	-80.718031
Puenta Sitio Nuevo	SEL	-2.001266	-80.721997
Puenta Las Javitas	SEL	-2.011276	-80.721306
Los Lojas	GUA	-2.012311	-79.957793
Isla Santay	GUA	-2.218169	-79.877790
Puento Carnero	SEL	-2.289010	-80.911768
El Real	SEL	-2.389893	-80.722320

Chandy	SEL	-2.406500	-80.691838
Puerto del Morro(mangel)	GUA	-2.609247	-80.303350
Puerto del Morro (dock)	GUA	-2.609984	-80.302150
Posorja (Boat yard)	GUA	-2.704980	-80.243030
Posorja (mangel)	GUA	-2.705939	-80.243208
Rio Villamil (Data)	GUA	-2.712594	-80.310690
Puerto Bolivar	ELO	-3.266135	-80.014533
Galapagos			
Isla San Cristóbal	GAL	-0.903160	-89.612997
Laguna de la Ninfor	GAL	-0.747401	-90.316190
Academic Bay lagoon	GAL	-0.751650	-90.310361
Playa Alemana	GAL	-0.752584	-90.310920
Punta Salida	GAL	-0.754628	-90.312979
Grais	GAL	-0.755988	-90.314705
Bahia Tortuga	GAL	-0.761403	-90.341072
Isla Isabella	GAL	-0.955922	-90.97145

Table S2. Specimens examined. Abbreviations: AMNH – F.H. Barnwell collection, American Museum of Natural History, NYC; RMNH – Naturalis Biodiversity Center, Leiden, the Netherlands; UNI – University of Northern Iowa collection, Cedar Falls, IA; USNM – U.S. Museum of Natural History (Smithsonian), Washington, D.C.; ZMA – Zoological Museum of Amsterdam (now in RMNH).

Species	Museum number	Origin	N	Remark
Uca heteropleura	AMNH_IZC 376846	Costa Rica, Puntarenas	127	
	RMNH.CRUS.D.23059	Peru, Puerto Pizarro	4	
	UNI 743	Ecuador, GUA, Posorja	6	
	UNI 744	Ecuador, MAN, San Clemente	2	
	UNI 753	Ecuador, ESM, S. Jose Chamanga	8	
	UNI 755	Ecuador, ESM, S. Jose Chamanga	3	
	USNM 138550	Ecuador, ELO, Puerto Bolivar	8	
Uca insignis	RMNH.CRUS.D.23063	Peru, Puerto Pizarro	5	
	USNM 138577	Ecuador, ELO, Puerto Bolivar	6	
	USNM 1521395	Peru, Lima, Pte Pizarro	2	
Uca intermedia	RMNH.CRUS.D.35788	Colombia, Buenavista	3	paratypes
	UNI 754	Ecuador ESM, El Rompido	8	
	UNI 881	Ecuador, MAN, San Clemente	1	
Uca ornata	AMNH_IZC 376865	Costa Rica, Puntarenas	12	
	RMNH.CRUS.D.23061	Peru, Puerto Pizarro	1	holotype *U. pizarri*
	RMNH.CRUS.D.23062	Ecuador, GUA, Gulf of GUA	1	paratype *U. pizarri*
	USNM 138614	Panama, Rio Abajo	5	
	USNM 138615	Panama, Old city	25	
	USNM 138616	Panama, Old city	31	
Uca princeps	AMNH_IZC 376866	Mexico, Baja California	7	
	AMNH_IZC 376867	Costa Rica, Puntarenas	26	
	AMNH_IZC 376877	Mexico, Colima	37	
	AMNH_IZC 376879	Mexico, Nayarit	47	
	AMNH_IZC 376881	Honduras, San Lorenzo	9	
	RMNH D250	Mexico, Gulf of California	2	
	RMNH.CRUS.D.7575	Mexico, Sonora, Guaymas	1	
	RMNH.CRUS.D.23060	Peru, Puerto Pizarro	11	
	UNI 741	Ecuador, GUA, Posorja	4	
	UNI 745	Ecuador, MAN, San Clemente	1	
	UNI 756	Ecuador, SEL, Punta Carnero	2	
	UNI 757	Ecuador, SEL, P. Sitio Nueva	1	
	UNI 758	Ecuador, ESM, S.J. Chamanga	1	
	UNI 854	Ecuador, Man, B. d, Caraquez	1	
	USNM 138633	Ecuador, ELO, Puerto Bolivar	33	
	USNM138634	Ecuador, ELO, Puerto Bolivar	3	
Uca stylifera	AMNH_IZC 376889	Costa Rica, Puntarenas	47	
	AMNH_IZC 376895	Costa Rica, Guanacaste	4	
	RMNH.CRUS.D.23058	Peru, Puerto Pizarro	4	
	UNI 742	Ecuador, MAN, B.d. Caráquez	5	
	UNI 759	Ecuador, ESM, El Rompido	1	
	UNI 760	Ecuador, ESM, S.J. Chamanga	2	

	UNI 761	Ecuador, ESM, S.J. Chamanga	5	
	USNM 32325	Costa Rica, Puntarenas	2	
	USNM 138836	Panama, Balboa	2	poor
	USNM 138837	Ecuador, ELO, Puerto Bolivar	1	
Petruca panamensis	AMNH_IZC 376770	Costa Rica, Puntarenas	18	
	AMNH_IZC 376772	Costa Rica, Guanacaste	5	
	UNI 752	Ecuador, ESM, Ply. Camerones	14	
	USNM 70989	Ecuador, SEL, Salinas	1	
	USNM 138625	Costa Rica, Golfito	12	
	USNM 138629	Colombia, Gorgonilla Is.	22	
	USNM 1294205	Panama, Culebra Is.	1	neotype
Minuca argillicola	AMNH_IZC 376418	Costa Rica, Puntarenas	34	
	AMNH_IZC 376428	Costa Rica, Guanacaste	16	
	UNI 786	Ecuador, ESM, La Tola	1	
	UNI 808	Ecuador, ESM, Mompiche	17	
	UNI 823	Ecuador, ESM Rio Agua Clara	25	
	UNI 824	Ecuador, ESM La Tola	16	
	UNI 825	Ecuador, ESM, Muisne	13	
	UNI 826	Ecuador, ESM, Rio Verde	14	
	UNI 827	Ecuador, ESM, El Rompido	4	
	UNI 828	Ecuador, ESM, Rio Clara	2	
	UNI 829	Ecuador, ESM, Rio Esmeraldas	1	
	UNI 855	Ecuador, ESM, Rio Verde	1	
	USNM 19441	Costa Rica, Santa Domingo	5	
	USNM 137400	Costa Rica, Golfito	1	holotype
	USNM 137401	Costa Rica, Golfito	2	paratypes
Minuca brevifrons	AMNH_IZC 376431	Costa Rica, Puntarenas	35	
	AMNH_IZC 376435	Costa Rica, Guanacaste	17	
	AMNH_IZC 376437	Mexico, Guerrero	5	
	AMNH_IZC 376438	Mexico, Oaxaca	1	
	AMNH_IZC 376439	Mexico, Jalisco	41	
	RMNH.CRUS.D7573	Mexico, Baja California	3	
	RMNH.CRUS.D9500	El Salvador, Rio Coyol	1	
	UNI 809	Ecuador, ESM, Rio Agua Clara	4	
	UNI 832	Ecuador, ESM, La Propicia	25	
	UNI 842	Ecuador, SEL, Puente Valdivia	1	
	UNI 856	Ecuador, ESM, La Tola	2	
	UNI 857	Ecuador, ESM, Muisne	1	
	UNI 858	Ecuador, ESM, Muisne	9	
	UNI 859	Ecuador, ESM, Rio Agua Clara	1	
	USNM 70867	Ecuador, GUA, Salado	1	
	USNM 138484	Costa Rica, Negritos	26	
Minuca ecuadoriensis	AMNH_IZC 376461	Mexico, Nayarit	136	
	AMNH_IZC 376462	Costa Rica, Guanacaste	57	
	AMNH_IZC 376466	Mexico, Baja California	2	
	AMNH_IZC 376469	Mexico, Oaxaca	13	
	AMNH_IZC 376477	Mexico, Colima	3	
	AMNH_IZC 376478	Mexico, Guerrero	3	

	AMNH_IZC 376486	Honduras, San Lorenzo	4	
	AMNH_IZC 376487	Costa Rica, Puntarenas	30	
	RMNH.CRUS.D23049	Peru, Puerto Pizarro	1	holotype *U. lanigra*
	RMNH.CRUS.D23050	Peru, Puerto Pizarro	15	paratypes *U. lanigra*
`	UNI 747	Ecuador, Man, Plya Don Juan	2	
	UNI 810	Ecuador, ESM, Rio Verde	13	
	UNI 811	Ecuador, ESM, La Tola	11	
	UNI 812	Ecuador, ESM, Rio Agua Clara	2	
	UNI 813	Ecuador, ESM, Mompiche	1	
	UNI 814	Ecuador, ESM, Sta Marta	2	
	UNI 815	Ecuador, ESM, Muisne	3	
	UNI 816	Ecuador, SEL, Pte Atravezando	1	
	UNI 817	Ecuador, SEL, Pto Silio Nuevo	2	
	USNM 70867	Ecuador, GUA, Salado	1	
	USNM 97756	Mexico, Nayarit	1	
Minuca aff. *ecuadoriensis*	UNI 746	Ecuador, GUA, Is. Santay	5	
Minuca galapagensis	AMNH_IZC 376489	Costa Rica, Puntarenas	8	
	RMNH.CRUS.D18797	Peru, Puerto Pizarro	1	
	RMNH.CRUS.D23048	Peru, Puerto Pizarro	15	
	RMNH.CRUS.D45382	Peru, Puerto Pizarro	2	
	UNI 748	Ecuador, MAN, Ply Don Juan	14	
	UNI 830	Ecuador, ESM, Muisne	1	
	UNI 831	Ecuador, GAL, Isla Sta Cruz	46	
	UNI 833	Ecuador, SEL, Simon Bolivar	8	
	UNI 834	Ecuador, ESM, Muisne	1	
	UNI 835	Ecuador, ESM, Rio Verde	5	
	UNI 836	Ecuador, ESM, Rio Agua Clara	6	
	UNI 837	Ecuador, SEL, Pto las Javitas	7	
	UNI 838	Ecuador, SEL, Pto Sitio Nuevo	2	
	UNI 839	Ecuador, GUA, Data Villamil	1	
	UNI 840	Ecuador, GAL, B. Tortuga	7	
	UNI 841	Ecuador, ESM, El Rompido	1	
	UNI 843	Ecuador, ESM, La Tola	13	
	UNI 844	Ecuador, SEL, Pte Atravezando	1	
	UNI 845	Ecuador, SEL, Manglaralto	2	
	UNI 846	Ecuador, SEL, Simon Bolivar	1	
	USNM 22319	Galapagos Islands, I Sta Cruz	6	syntypes
	USNM 98046	Ecuador, GUA, Guayas	1	
	USNM 138536	Ecuador, ELO, Puerto Bolivar	35	
Minuca herradurensis	AMNH_IZC 376500	Costa Rica, Puntarenas	86	
	AMNH_IZC 376506	Mexico, Oaxaca	5	
	AMNH_IZC 376584	Honduras, Rio Coyol	53	
	AMNH_IZC 376615	Mexico, Salina Cruz	3	
	USNM 123790	Costa Rica, Puntarenas	14	
	USNM 138542	Panama, Old city	89	
Minuca osa	RMNH.CRUS.D53098	Costa Rica, Finca Saladero	2	paratypes
	UNI 818	Ecuador, ESM, Rio Agua Clara	4	

	UNI 819	Ecuador, ESM, Pta Atravezando	3	
	UNI 820	Ecuador, ESM, La Tola	5	
	UNI 821	Ecuador, SEL, Rio Caldeate	1	
	UNI 822	Ecuador, ESM, La Tola	6	
	UNI 860	Ecuador, ESM, Rio Verde	3	
Minuca zacae	AMNH_IZC 376370	Mexico, Colima	9	
	AMNH_IZC 376715	Mexico, Oaxaca	80	
	AMNH_IZC 376716	Mexico, Nayarit	10	
	AMNH_IZC 376718	Costa Rica, Puntarenas	32	
	AMNH_IZC 376723	Mexico, Guerrero	15	
	AMNH_IZC 376728	Costa Rica, Guanacaste	93	
	USNM 79408	Nicaragua, Corinto	4	
	USNM 137426	Costa Rica, Golfito	1	holotype
	USNM 137427	Costa Rica, Golfito	27	paratypes
	ZMA.CRUS.D.100-365	El Salvador, Los Blancós	8	
Leptuca batuenta	AMNH_IZC 376053	Costa Rica, Puntarenas	8	
	AMNH_ZC 376056	Honduras, San Lorenzo	1	
	RMNH.CRUS.D23045	Peru, Puerto Pizzaro	10	
	UNI 861	Ecuador, ESM, El Rompido	1	
	USNM 79399	Costa Rica, Puntarenas	3	paratypes
	USNM 137404	Costa Rica, Ballenas Bay	6	paratypes
	USNM 137405	Panama, Balboa	1	holotype
	USNM 137406	Panama, Balboa	3	paratypes
	USNM 138473	Ecuador, ELO, Puerto Bolivar	3	
Leptuca beebei	AMNH_IZC 376059	Mexico, Manzanilla Bay	1	
	AMNH_IZC 376060	Mexico, Guerrero	7	
	AMNH_IZC 376061	Costa Rica, Puntarenas	5	
	AMNH_IZC 376070	Honduras, San Lorenzo	11	
	AMNH_IZC 376086	Costa Rica, Guanacaste	3	
	RMNH.CRUS.D9499	El Salvador, Gulf de Fonseca	1	
	RMNH.CRUS.D23056	Peru, Puerto Pizarro	15	
	UNI 762	Ecuador, ESM San Lorenzo	1	
	UNI 763	Ecuador, GUA, Posjora	14	
	UNI 764	Ecuador, MAN, B. d. Caraquez	3	
	UNI 791	Ecuador, SEL, Simon Bolivar	1	
	UNI 793	Ecuador, GUA, Porsorja	1	
	UNI 794	Ecuador, ELO, Puerto Bolivar	6	
	UNI 865	Ecuador, ESM, S.J. Chamanga	42	
	USNM 79400	Panama, Balboa	4	topotypes
	USNM 137413	Panama, Balboa	1	holotype
	USNM 137414	Panama, Balboa	16	paratypes
	USNM 138480	Ecuador, ELO, Puerto Bolivar	8	
Leptuca deichmanni	AMNH_IZC 376104	Costa Rica, Puntarenas	1	
	AMNH_IZC 376105	Costa Rica, Puntarenas	36	
	AMNH_IZC 376106	Costa Rica, Guanacaste	6	
	AMNH_IZC 376108	Honduras, San Lorenzo	15	
	UNI 765	Ecuador, ESM, Ply Achilube	90	
	UNI 766	Ecuador, ESM, S.J. Chamanga	26	

	UNI 767	Ecuador, ESM, San Lorenzo	3	
	UNI 772	Ecuador, SEL, Simon Bolivar	2	
	UNI 792	Ecuador, ESM, Mompiche	1	
	UNI 868	Ecuador, ESM, S. Jose Chamanga	1	
	UNI 890	Ecuador, MAN, Bahia de Caraquez	1	
	USNM 70832	Panama, Canal entrance	1	type
	USNM 138521	Costa Rica, Port Parker	16	
	USNM 138524	Costa Rica, Golfito	10	
	USNM 138526	Panama, Panama City	13	
	USNM 138525	Panama, Honda Bay	1	
Leptuca dorotheae	AMNH_IZC 376113	Costa Rica, Puntarenas	20	
	AMNH_IZC 376114	Costa Rica, Puntarenas	3	
	RMNH.CRUS.D23054	Peru, Puerto Pizarro	1	holotype
	RMNH.CRUS.D23055	Peru, Puerto Pizarro	2	paratypes
	UNI 771	Ecuador, ESM, S.J. Chamanga	42	
	UNI 773	Ecuador, SEL, Pta. Carnero	3	
	USNM 138534	Ecuador, ELO, Puerto Bolivar	12	
	USNM 138535	Panama, Balboa	2	
Leptuca festae	AMNH_ IZC 376115	Costa Rica, Puntarenas	120	
	AMNH_IZC 376118	Costa Rica, Guanacaste	9	
	UNI 751	Ecuador, GUA, Rio Guayas	6	
	UNI 770	Ecuador, ESM, El Rompido	1	
	UNI 774	Ecuador, ESM, Rio Esmeraldas	4	
	UNI 775	Ecuador, ESM, Pte. Montalvo	27	
	UNI 776	Ecuador, ESM, Rio Agua Clara	1	
	USNM 70870	Ecuador, GUA, Guayaquil	171	
	USNM 138533	Ecuador, GUA, Guayaquil	90	
Leptuca helleri	UNI 777	Ecuador, GAL, Is. Sta Cruz	25	
	UNI 778	Ecuador, GAL, B. Tortuga	6	
	USNM 24829	Galapagos, Narborough I.	4	lectotypes, syntypes
	USNM 25666	Galapagos, Narborough I.	1	
	USNM 63154	Galapagos, Black Bight	2	
Leptuca inaequalis	AMNH_IZC 376132	Costa Rica, Puntarenas	12	
	AMNH_IZC 376138	Honduras, San Lorenzo	13	
	AMNH_IZC 376140	Costa Rica, Guanacaste	6	
	RMNH.CRUS.D23043	Peru, Puerto Pizarro	17	
	UNI 779	Ecuador, ESM, San Lorenzo	4	
	UNI 780	Ecuador, ESM, El Rompido	1	
	UNI 781	Ecuador, ESM, Rio Verde	1	
	UNI 782	Ecuador, SEL Pto. Sitio Nuevo	2	
	UNI 871	Ecuador, ESM, S.J. Chamanga	1	
	USNM 70833	Ecuador, GUA, Salado	10	types
	USNM 138551	Ecuador, ELO, Puerto Bolivar	9	
	USNM 138552	Ecuador, GUA, Salado	32	
Leptuca latimanus	AMNH_IZC 376143	Mexico, Nayarit	93	
	AMNH_IZC 376146	Costa Rica, Guanacaste	17	
	AMNH_IZC 376148	Mexico, Oaxaca	4	
	AMNH_IZC 376149	Costa Rica, Puntarenas	1	

	AMNH_IZC 376154	Mexico, Oaxaca	1	
	RMNH.CRUS.D23052	Peru, Puerto Pizarro	15	
	UNI 783	Ecuador, SEL, Simon Bolivar	10	
	UNI 784	Ecuador, SEL, Pta. Las Javitas	2	
	UNI 785	Ecuador, SEL, Rio Cadeate	1	
	UNI 787	Ecuador, ESM, Ply Camerones	1	
	UNI 788	Ecuador, SEL, Manglaralto	1	
	UNI 789	Ecuador, GUA, Posorja	5	
	USNM 17500	Mexico, La Paz	1	type - *Gelasimus*
	USNM 138565	Panama, Balboa	32	
	USNM 138566	Ecuador, ELO, Puerto Bolivar	2	
Leptuca pygmaea	AMNH_IZC 376256	Costa Rica, Puntarenas	38	
	AMNH_IZC 376258	Honduras, San Lorenzo	4	
	USNM 139419	Costa Rica, Golfito	1	holotype
	USNM 137420	Costa Rica, Golfito	10	paratypes
	USNM 138646	Colombia, Buenaventura	1	
	USNM 1521269	Panama, Canal spillway	1	
Leptuca saltitanta	AMNH_IZC 376260	Costa Rica, Puntarenas	21	
	UNI 795	Ecuador, ESM, El Rompido	9	
	UNI 869	Ecuador, ESM, San Lorenzo	1	
	USNM 79403	Panama, Balboa	8	topotypes
	USNM 137407	Panama, Balboa	1	type
	USNM 137408	Panama, Balboa	14	
	USNM 138822	Panama, Balboa	130	
Leptuca stenodactylus	AMNH_IZC 376311	Honduras, San Lorenzo	3	
	AMNH_IZC 376317	Costa Rica, Puntarenas	15	
	AMNH_IZC 376319	Costa Rica, Guanacaste	3	
	RMNH.CRUS.D23057	Peru, Puerto Pizarro	8	
	UNI 749	Ecuador, GUA, Posorja	1	
	UNI 768	Ecuador, ESM, S. Jose Chamanga	2	
	UNI 769	Ecuador, ESM, Rio Verde	1	
	USNM 138826	Nicaragua, Corinto	31	
	USNM 138830	Panama, Bella Vista	13	
	USNM 138833	Ecuador, ELO, Puerto Bolivar	4	
Leptuca tallanica	RMNH.CRUS.D23046	Peru, Puerto Pizarro	1	holotype
	RMNH.CRUS.D23047	Peru, Puerto Pizarro	8	paratypes
	UNI 862	Ecuador, ESM, El Rompido	1	
	UNI 863	Ecuador, ESM, Musine	1	
	UNI 864	Ecuador, ESM, Simon Bolivar	1	
	UNI 867	Ecuador, ESM, Rio Verde	5	
	USNM 138838	Ecuador, ELO, Puerto Bolivar	7	
Leptuca tenuipedis	AMNH_IZC 376330	Costa Rica, Guanacaste	3	
	AMNH_IZC 376331	Costa Rica, Puntarenas	3	
	RMNH.CRUS.D23044	Peru, Puerto Pizarro	4	not good
	UNI 866	Ecuador, ESM, Mompiche	23	
	UNI 891	Ecuador, MN, Bahia de Caraquez	1	
	USNM 79404	Costa Rica, Ballenas Bay	2	paratypes
	USNM 137409	Costa Rica, Ballenas Bay	1	holotype

	USNM 137410	Costa Rica, Ballenas Bay	9	paratypes
Leptuca terpsichores	AMNH_IZC 376335	Costa Rica, Puntarenas	6	
	AMNH_IZC 376372	Costa Rica, Guanacaste	1	
	RMNH.CRUS.D23053	Peru, Puerto Pizarro	5	
	UNI 796	Ecuador, ESM, El Rompido	12	
	UNI 797	Ecuador, ESM Mompiche	23	
	UNI 798	Ecuador, ESM, San Lorenzo	16	
	UNI 799	Ecuador, SEL, Pta Carnero	8	
	UNI 800	Ecuador, ESM, Mompiche	2	
	USNM 137417	Panama	1	holotype
	USNM 137418	Panama	2	paratypes
	USNM 138608	Ecuador, ELO, Jambeli Is.	3	
Leptuca tomentosa	AMNH_IZC 376360	Costa Rica, Puntarenas	75	
	AMNH_IZC 376398	Costa Rica, Guanacaste	34	
	RMNH.CRUS.D23051	Peru, Puerto Pizarro	6	
	UNI 750	Ecuador, GUA, Data Villamil	6	
	UNI 801	Ecuador, ESM, Muisne	6	
	UNI 802	Ecuador, ESM, Rio Verde	11	
	UNI 803	Ecuador, ESM, El Rompido	10	
	UNI 804	Ecuador, SEL, Simon Bolivar	4	
	USNM 79406	Costa Rica, Puntarenas	1	not good
	USMN 137411	Panama, Puntarenas	1	holotype
	USNM 137412	Panama, Puntarenas	3	paratypes
	USNM 138839	Costa Rica, Golfito	1	
	USNM 138840	Panama , Panama City	6	
	USNM 1521275	Panama, Canal spillway	5	
Leptuca umbratila	AMNH_IZC 376400	Costa Rica, Puntarenas	8	
	AMNH_IZC 376402	Honduras, San Lorenzo	8	
	AMNH_IZC 376407	Costa Rica, Puntarenas	15	
	UNI 805	Ecuador, ESM La Rompido	8	
	UNI 806	Ecuador, ESM, Rio Verde	5	
	UNI 807	Ecuador, ESM, La Tola	14	
	USNM 79407	Canal Zone , Balboa	1	paratype
	USNM 138132	Costa Rica, Nicoya	1	holotype
	USNM 138133	Costa Rica, Nicoya	1	paratype
