# Supplementary material for: Fiddler Crabs (Crustacea: Decapoda: Ocypodidae) From Coastal Ecuador and the Galápagos Islands: Species Descriptions and DNA Barcodes
Source: Ecol Evol. 2025 Jan 27;15(1):e70646. doi: 10.1002/ece3.70646 (PMC11770328; doi:10.1002/ece3.70646)
Supplement: Supplementary file 2 — Table S3. Matrix of percentage of pairwise nucleotide divergences with Kimura 2‐parameter (K2P) distances based on the cytochrome c oxidase subunit I (COI) gene within and between 30 species of fiddler crabs from Ecuador and adjacent areas. Range of values are given in parentheses. [file ECE3-15-e70646-s002.docx]

**Table S3**. Matrix of percentage of pairwise nucleotide divergences with Kimura 2-parameter (K2P) distances based on the cytochrome c oxidase subunit I (COI) gene within and between 30 species of fiddler crabs from Ecuador and adjacent areas. Range of values are given in parentheses.

|  | Intraspecific | Interspecific |  |  |  |  |  |  |  |  |  |  |  |  |  |
| --- | --- | --- | --- | --- | --- | --- | --- | --- | --- | --- | --- | --- | --- | --- | --- |
|  | Nucleotide  divergence | *L.*  *tomentosa* | *L.*  *tallanica* | *L.*  *stenodactylus* | *L.*  *beebei* | *L.*  *dorotheae* | *L.*  *latimanus* | *L.*  *terpsichores* | *L.*  *inaequalis* | *L.*  *festae* | *L.*  sp. | *L.* aff.  *dorotheae* | *L.*  *saltitana* | *L.*  *batuenta* | *L.*  *umbratila* |
| *L. tomentosa* | 1.23  (1.23-1.23) |  |  |  |  |  |  |  |  |  |  |  |  |  |  |
| *L. tallanica* | 0.4  (0-0.76) | 15.61  (15.41-15.82) |  |  |  |  |  |  |  |  |  |  |  |  |  |
| *L. stenodactylus* | 0 | 14.68  (14.57-14.78) | 1.89 (1.85-2.01) |  |  |  |  |  |  |  |  |  |  |  |  |
| *L. beebei* | 0 | 15.53  (15.42-15.64) | 3.58 (3.46-3.78) | 3.6  (3.6-3.6) |  |  |  |  |  |  |  |  |  |  |  |
| *L. dorotheae* | 0.83  (0-1.23) | 15.07  (14.47-15.64) | 10.99 (10.36-11.44) | 10.53  (9.99-10.88) | 11.59  (11.26-11.8) |  |  |  |  |  |  |  |  |  |  |
| *L. latimanus* | 0 | 16.48  (16.27-16.69) | 13.16 (12.83-13.38) | 12.08  (12.08-12.08) | 13.76  (13.76-13.76) | 13.47  (13.05-13.81) |  |  |  |  |  |  |  |  |  |
| *L. terpsichores* | 1.23  (1.07-1.38) | 16.09  (15.66-16.46) | 12.98 (12.66-13.42) | 13.04  (13.03-13.04) | 14.83  (14.56-15.36) | 12.58  (12.13-13.24) | 10.63  (10.39-10.75) |  |  |  |  |  |  |  |  |
| *L. inaequalis* | 0.61  (0.61-0.61) | 12.09  (11.44-12.74) | 11.32 (11.01-11.74) | 10.99  (10.81-11.17) | 11.75  (11.56-11.93) | 9.72  (9.43-9.96) | 11.89  (11.89-11.89) | 13.28  (12.96-13.72) |  |  |  |  |  |  |  |
| *L. festae* | 1.02  (0.61-1.23) | 11.33  (10.85-11.79) | 10.34 (9.78-11.02) | 10.23  (9.76-10.47) | 10.5  (10.5-10.5) | 11.11  (10.88-11.43) | 13.17  (12.8-13.55) | 13.66  (13.16-14.11) | 8.93  (8.92-8.93) |  |  |  |  |  |  |
| *L.* sp. | 0.77  (0.77-0.77) | 11.1  (10.64-11.56) | 12.17 (11.94-12.5) | 12.37  (12.28-12.47) | 12.23  (12.14-12.32) | 12.47  (12.32-12.7) | 13.57  (13.19-13.94) | 14.78  (14.45-15.23) | 10.08  (9.81-10.35) | 7.58  (7.31-7.85) |  |  |  |  |  |
| *L.* aff. *dorotheae* | 0.71  (0.46-0.92) | 11.91  (11.54-12.28) | 11.04 (10.66-11.57) | 10.88  (10.64-11.18) | 12.01  (11.77-12.32) | 10.77  (10.13-11.4) | 11.43  (11.19-11.74) | 14.04  (13.53-14.68) | 8.15  (8.04-8.21) | 6.38  (5.75-6.95) | 6.76  (6.1-7.31) |  |  |  |  |
| *L. saltitana* | 0.41  (0.3-0.46) | 14.49  (13.89-15.03) | 13.88 (13.66-14.22) | 13.78  (13.66-14.03) | 14.47  (14.22-14.59) | 12.56  (12.03-13.14) | 15.05  (14.98-15.17) | 15.4  (15.14-15.53) | 11.84  (11.48-12.2) | 12.83  (12.4-13.32) | 12.19  (11.86-12.4) | 11.56  (11.14-12.04) |  |  |  |
| *L. batuenta* | 0 | 12.02  (11.75-12.29) | 12.51 (12.47-12.64) | 11.54  (11.54-11.54) | 12.29  (12.29-12.29) | 13.4  (13.21-13.59) | 14.48  (14.48-14.48) | 15.35  (15.22-15.6) | 10.37  (10.28-10.46) | 9.78  (9.43-10.14) | 9.96  (9.96-9.96) | 9.2  (9.08-9.25) | 9.91  (9.74-10.09) |  |  |
| *L. umbratila* | 0.54  (0.16-0.82) | 15.29  (14.99-15.55) | 12.53 (12.19-13.16) | 12.95  (12.76-13.32) | 13.37  (13.19-13.73) | 13.24  (12.39-14.11) | 13.15  (12.97-13.52) | 15.03  (14.59-16) | 10.43  (10.09-10.82) | 10.16  (9.93-10.52) | 10.82  (10.51-11.09) | 9.22  (8.99-9.79) | 11.79  (11.38-12.14) | 9.57  (9.56-9.6) |  |
| *L. helleri* | 0.72  (0-1.23) | 13.17  (12.37-13.69) | 14.42 (14.16-14.73) | 14.27  (14.12-14.32) | 14.68  (14.53-14.73) | 12.2  (11.03-13.08) | 14.98  (14.55-15.13) | 15.47  (14.87-16.04) | 11.94  (11.43-12.36) | 11.62  (10.89-12) | 12.74  (12.17-13.12) | 12.59  (12.15-13.09) | 12.99  (12.45-13.2) | 13.46  (13.18-13.55) | 14.34  (13.93-14.79) |
| *L. deichmanni* | 1.2  (0.61-1.86) | 14.12  (13.28-14.99) | 13.55 (13-13.92) | 13.12  (12.96-13.34) | 13.85  (13.55-14.12) | 12.47  (11.73-13.23) | 12.01  (11.54-12.26) | 15.04  (14.25-15.78) | 11.68  (11.2-11.94) | 12.03  (11.77-12.32) | 12.06  (11.78-12.53) | 11.57  (11.02-11.95) | 12.72  (12.45-13) | 13.3  (12.99-13.56) | 13.71  (13.17-14.33) |
| *M. brevifrons* | 0.86  (0.46-1.4) | 15.35  (14.67-16.04) | 14.68 (14.25-15.22) | 14.17  (13.68-14.44) | 14.62  (14.07-15.05) | 15.96  (15.23-16.63) | 16.72  (16.36-17.16) | 17.3  (16.53-18.14) | 13.82  (13.31-14.25) | 14.24  (13.48-14.65) | 14.79  (14.1-15.45) | 13.78  (12.94-14.48) | 14.39  (14.19-14.77) | 13.05  (12.61-13.36) | 12.53  (11.83-13.4) |
| *M. zacae* | 0 | 12.92  (12.34-13.51) | 16.41 (16.05-16.66) | 16.45  (16.45-16.45) | 16.89  (16.89-16.89) | 16.79  (16.74-16.95) | 15.13  (15.13-15.13) | 15.06  (14.73-15.55) | 13.49  (13.49-13.49) | 12.92  (12.72-13.12) | 12.76  (12.37-13.15) | 13.46  (13.33-13.52) | 14.6  (14.26-14.86) | 12.75  (12.75-12.75) | 14.34  (14.06-14.7) |
| *M. ecuadoriensis* | 0.61  (0.3-0.77) | 16.93  (16.77-17.16) | 16.29 (15.95-16.73) | 15.79  (15.73-15.92) | 17.14  (16.94-17.34) | 15.42  (14.63-16.37) | 15.93  (15.6-16.19) | 15.76  (15.4-16.17) | 13.96  (13.52-14.27) | 14.65  (14.08-15.03) | 13.85  (13.53-14.1) | 13.71  (13.14-14.08) | 15.44  (14.8-15.96) | 13.78  (13.71-13.9) | 14.69  (14.3-14.99) |
| *M.* aff. *ecuadoriensis* | 0 | 15.5  (15.41-15.59) | 16.16 (16.11-16.3) | 16.28  (16.28-16.28) | 16.51  (16.51-16.51) | 15.28  (14.81-15.77) | 16.18  (16.18-16.18) | 15.84  (15.77-15.96) | 14.09  (13.9-14.28) | 13.77  (13.53-14.08) | 13.73  (13.54-13.92) | 13.15  (13.15-13.15) | 13.99  (13.67-14.24) | 12.6  (12.6-12.6) | 14.62  (14.1-14.98) |
| *M.* aff. *zacae* | 0.46  (0.46-0.46) | 15.6  (15.41-15.8) | 15.39 (15.34-15.53) | 14.96  (14.77-15.14) | 15.55  (15.36-15.74) | 14.69  (14.44-15.03) | 14.55  (14.46-14.65) | 14.04  (13.7-14.44) | 12.6  (12.41-12.79) | 13.12  (12.79-13.52) | 12.8  (12.61-12.99) | 13.12  (12.79-13.34) | 13.7  (13.3-14.04) | 11.61  (11.34-11.88) | 13.26  (12.98-13.56) |
| *M. osa* | 0.84  (0.61-1.08) | 15.25  (14.86-15.83) | 14.44 (14.03-14.81) | 14.5  (14.21-14.77) | 15.28  (14.98-15.55) | 15.36  (14.83-15.99) | 14.7  (14.64-14.84) | 16.24  (15.99-16.57) | 12.41  (12.04-12.59) | 11.64  (11.32-11.87) | 13.61  (13.19-14.12) | 12.61  (12.24-12.79) | 12.88  (12.38-13.47) | 11.75  (11.52-12.06) | 12.04  (11.52-12.58) |
| *M. argillicola* | 1.13  (0.77-1.7) | 15.51  (15.3-15.73) | 17.34 (17.11-17.7) | 17.88  (17.67-18.07) | 17.13  (16.92-17.32) | 16.1  (15.59-16.77) | 17.32  (17.16-17.39) | 16.82  (16.43-17.19) | 14.95  (14.66-15.26) | 12.77  (12.08-13.21) | 15.32  (14.53-15.93) | 13.72  (13.36-14.15) | 14.32  (14.04-14.61) | 15.72  (15.22-16.03) | 14.46  (13.92-15.19) |
| *M. galapagensis* | 0.73  (0.3-1.23) | 14.06  (13.53-14.47) | 16.13 (15.34-17.09) | 16.25  (15.91-16.89) | 16.77  (16.51-17.11) | 15.23  (14.62-15.77) | 14.71  (14.44-15.01) | 16.27  (15.99-16.57) | 12.17  (11.86-12.59) | 11.53  (11.17-11.9) | 13.29  (12.64-13.97) | 11.04  (10.62-11.34) | 13.69  (13.29-14.04) | 12.14  (12.06-12.25) | 13.34  (13.18-13.59) |
| *U. princeps* | 0.12  (0-0.3) | 21.27  (20.91-22.38) | 19.61 (19.25-20.77) | 20.05  (19.88-21.03) | 20.39  (20.09-21.25) | 18.82  (18.6-19.43) | 21.81  (21.52-22.58) | 22.81  (22.08-23.91) | 19.72  (19.4-20.52) | 18.81  (18.3-19.78) | 17.16  (17.08-17.58) | 17.26  (16.86-17.77) | 19.19  (18.99-20.07) | 17.41  (17.25-18.17) | 19.82  (19.44-20.55) |
| *U. insignis* | 0 | 20.68  (20.27-21.09) | 18.46 (18.26-18.66) | 18.28  (18.28-18.28) | 19.09  (19.09-19.09) | 20.59  (20.25-20.88) | 21.32  (21.32-21.32) | 21.63  (21.47-21.92) | 19.78  (19.78-19.78) | 19.52  (19.26-19.66) | 17.33  (17.03-17.62) | 19.01  (18.81-19.42) | 20.37  (20.16-20.58) | 18.23  (18.23-18.23) | 19.79  (19.67-19.89) |
| *U. intermedia* | 1.03  (0.46-1.39) | 20.09  (19.54-20.36) | 18.43 (17.47-19.08) | 19.01  (18.67-19.28) | 17.82  (17.48-18.09) | 18.5  (18.11-18.93) | 20.34  (19.92-20.77) | 22.5  (22.13-23.02) | 18.18  (17.87-18.48) | 17.76  (17.09-18.1) | 17.97  (17.87-18.07) | 17.19  (16.86-17.45) | 18.13  (18.04-18.24) | 19.24  (19.11-19.52) | 19.1  (18.67-19.51) |
| *U. heteropleura* | 0.31  (0-0.46) | 20.74  (20.33-21.37) | 19.7 (19.49-19.89) | 19.89  (19.89-19.89) | 20.2  (19.92-20.34) | 21.1  (20.52-21.59) | 22.07  (22-22.22) | 24.78  (24.31-25.24) | 20.09  (19.85-20.27) | 19.2  (18.89-19.51) | 18.84  (18.67-19.08) | 18.44  (18.04-18.85) | 19.47  (19.4-19.61) | 19.55  (19.48-19.68) | 20.01  (19.67-20.25) |
| *U. stylifera* | 0.33  (0-0.65) | 23.58  (22.66-24.06) | 19.66 (18.87-20.14) | 19.65  (19.07-19.92) | 19.29  (18.89-19.51) | 22.29  (21.8-22.68) | 20.79  (20.57-20.87) | 23.38  (22.58-23.79) | 21.9  (21.29-22.35) | 20.27  (19.49-20.85) | 21.09  (20.51-21.5) | 19.75  (19.44-19.91) | 22.54  (21.89-22.99) | 21.72  (21.12-21.92) | 19.85  (19.44-20.56) |
| *P. panamensis* | 0.52  (0.3-0.92) | 22.15  (21.8-22.44) | 25.14 (24.78-25.71) | 25.72  (25.45-25.92) | 25.7  (25.5-25.98) | 26.11  (25.39-26.82) | 26.22  (25.71-26.66) | 24.28  (23.19-24.98) | 26.36  (26.01-26.72) | 25.41  (25.11-25.79) | 24.26  (23.8-24.73) | 23.26  (22.65-23.8) | 27.43  (26.99-28.2) | 25.76  (25.33-26.04) | 27.87  (27-28.58) |

|  |  |  |  |  |  |  |  |  |  |  |  |  |  |  |  |  |
| --- | --- | --- | --- | --- | --- | --- | --- | --- | --- | --- | --- | --- | --- | --- | --- | --- |
|  | *L.*  *helleri* | *L.*  *deichmanni* | *M.*  *brevifrons* | *M.*  *zacae* | *M.*  *ecuadoriensis* | *M.* aff.  *ecuadoriensis* | *M.* aff.  *zacae* | *M.*  *osa* | *M.*  *argillicola* | *M.*  *galapagensis* | *U.*  *princeps* | *U.*  *insignis* | *U.*  *intermedia* | *U.*  *heteropleura* | *U.*  *stylifera* | *P.*  *panamensis* |
| *L. tomentosa* |  |  |  |  |  |  |  |  |  |  |  |  |  |  |  |  |
| *L. tallanica* |  |  |  |  |  |  |  |  |  |  |  |  |  |  |  |  |
| *L. stenodactylus* |  |  |  |  |  |  |  |  |  |  |  |  |  |  |  |  |
| *L. beebei* |  |  |  |  |  |  |  |  |  |  |  |  |  |  |  |  |
| *L. dorotheae* |  |  |  |  |  |  |  |  |  |  |  |  |  |  |  |  |
| *L. latimanus* |  |  |  |  |  |  |  |  |  |  |  |  |  |  |  |  |
| *L. terpsichores* |  |  |  |  |  |  |  |  |  |  |  |  |  |  |  |  |
| *L. inaequalis* |  |  |  |  |  |  |  |  |  |  |  |  |  |  |  |  |
| *L. festae* |  |  |  |  |  |  |  |  |  |  |  |  |  |  |  |  |
| *L.* sp. |  |  |  |  |  |  |  |  |  |  |  |  |  |  |  |  |
| *L.* aff. *dorotheae* |  |  |  |  |  |  |  |  |  |  |  |  |  |  |  |  |
| *L. saltitana* |  |  |  |  |  |  |  |  |  |  |  |  |  |  |  |  |
| *L. batuenta* |  |  |  |  |  |  |  |  |  |  |  |  |  |  |  |  |
| *L. umbratila* |  |  |  |  |  |  |  |  |  |  |  |  |  |  |  |  |
| *L. helleri* |  |  |  |  |  |  |  |  |  |  |  |  |  |  |  |  |
| *L. deichmanni* | 8.62  (7.61-9.22) |  |  |  |  |  |  |  |  |  |  |  |  |  |  |  |
| *M. brevifrons* | 16.58  (15.95-17.34) | 16.09  (15.4-16.96) |  |  |  |  |  |  |  |  |  |  |  |  |  |  |
| *M. zacae* | 15.7  (15.49-15.91) | 15.67  (15.46-15.88) | 13.31  (12.98-13.59) |  |  |  |  |  |  |  |  |  |  |  |  |  |
| *M. ecuadoriensis* | 18.15  (17.36-18.58) | 16.67  (15.96-17.16) | 13.23  (12.79-13.59) | 10.52  (10.2-10.78) |  |  |  |  |  |  |  |  |  |  |  |  |
| *M.* aff. *ecuadoriensis* | 16.92  (16.37-17.37) | 14.99  (14.79-15.18) | 13.83  (13.35-14.15) | 11.18  (11.18-11.18) | 4.54  (4.26-4.76) |  |  |  |  |  |  |  |  |  |  |  |
| *M.* aff. *zacae* | 15.31  (14.83-15.98) | 14.78  (14.42-14.99) | 13.52  (12.98-13.96) | 10.54  (10.25-10.82) | 4.84  (4.43-5.25) | 4.85  (4.6-5.09) |  |  |  |  |  |  |  |  |  |  |
| *M. osa* | 15.67  (15.03-16) | 14.73  (14.42-15.2) | 10.41  (9.87-10.83) | 10.54  (10.27-10.83) | 7.71  (7.45-7.96) | 7.58  (7.28-7.97) | 7.6  (7.31-7.81) |  |  |  |  |  |  |  |  |  |
| *M. argillicola* | 15.76  (15.06-16.04) | 14.71  (14.06-15.04) | 14.8  (13.92-15.69) | 11.55  (11.5-11.69) | 12.86  (12.22-13.37) | 11.4  (11.12-11.86) | 10.12  (9.85-10.56) | 9.8  (9.33-10.59) |  |  |  |  |  |  |  |  |
| *M. galapagensis* | 15.17  (14.47-15.83) | 14.76  (14.05-15.4) | 12.86  (11.66-13.57) | 9.13  (8.68-9.42) | 9.93  (9.68-10.22) | 9.46  (9.33-9.69) | 9.02  (8.62-9.52) | 9.28  (8.95-9.86) | 8.83  (8.27-9.7) |  |  |  |  |  |  |  |
| *U. princeps* | 21.44  (20.7-22.19) | 20.46  (19.99-21.16) | 19.48  (19.06-20.56) | 19.47  (19.35-19.86) | 19.19  (18.83-20.11) | 18.36  (18.21-19.01) | 19.44  (19.02-20.34) | 18.8  (18.24-19.72) | 18.53  (17.91-19.59) | 18.08  (17.27-19.1) |  |  |  |  |  |  |
| *U. insignis* | 20.95  (20.63-21.05) | 20.75  (20.16-21.41) | 19.58  (19.2-20.02) | 17.87  (17.87-17.87) | 17.45  (17.25-17.65) | 19.03  (19.03-19.03) | 18.13  (18.03-18.22) | 17.88  (17.63-18.22) | 17.06  (16.7-17.33) | 17.91  (17.48-18.49) | 14.61  (14.4-14.8) |  |  |  |  |  |
| *U. intermedia* | 18.38  (18.07-18.89) | 19.33  (18.65-20.09) | 18.78  (18.08-19.28) | 18.82  (18.75-18.96) | 18.42  (17.88-18.9) | 18.79  (18.66-18.86) | 18.8  (18.47-19.07) | 18.1  (17.67-18.49) | 18.3  (18.14-18.55) | 17.13  (16.49-18.07) | 15.52  (14.86-16.48) | 15.78  (15.44-16.04) |  |  |  |  |
| *U. heteropleura* | 20.57  (20.12-20.75) | 20.67  (19.89-20.96) | 19.11  (18.61-19.43) | 18.39  (18.25-18.67) | 18.53  (18.19-19) | 20.33  (20.19-20.61) | 20.65  (20.41-21.03) | 18.02  (17.65-18.62) | 18.65  (18.28-19.27) | 18.36  (17.99-18.79) | 13.9  (13.51-14.76) | 16.06  (16.06-16.06) | 12.55  (12.24-12.81) |  |  |  |
| *U. stylifera* | 22.84  (22.15-23.3) | 23.1  (22.1-23.71) | 21.08  (20.46-21.45) | 22.97  (22.69-23.07) | 21.72  (20.49-24.28) | 22.59  (22.15-22.81) | 22.51  (21.95-22.81) | 21.73  (21.11-22.19) | 20.87  (19.92-21.57) | 20.66  (20.09-21.29) | 14.82  (13.97-15.46) | 16.03  (15.74-16.4) | 14.52  (13.65-15.59) | 14.09  (13.44-14.48) |  |  |
| *P. panamensis* | 21.97  (21.38-22.26) | 26.71  (26.04-27.24) | 27.78  (26.93-28.54) | 22.21  (21.86-22.56) | 24.4  (24.05-24.74) | 25.05  (24.78-25.25) | 24.87  (24.34-25.48) | 24.77  (24.14-25.55) | 26.15  (25.56-26.76) | 26.15  (25.77-26.72) | 24.4  (23.77-25.05) | 28.53  (28.2-29.18) | 26.56  (25.96-27.38) | 24  (23.93-24.38) | 26.81  (26.35-27.33) |  |
